# Maternal insulin resistance multigenerationally impairs synaptic plasticity and memory via gametic mechanisms

**DOI:** 10.1038/s41467-019-12793-3

**Published:** 2019-10-22

**Authors:** Salvatore Fusco, Matteo Spinelli, Sara Cocco, Cristian Ripoli, Alessia Mastrodonato, Francesca Natale, Marco Rinaudo, Giulia Livrizzi, Claudio Grassi

**Affiliations:** 1 Fondazione Policlinico Universitario A. Gemelli IRCCS, 00168 Rome, Italy; 2 Institute of Human Physiology, Università Cattolica del Sacro Cuore, 00168 Rome, Italy

**Keywords:** Epigenetics in the nervous system, Neurotrophic factors, Synaptic plasticity, Pre-diabetes

## Abstract

Metabolic diseases harm brain health and cognitive functions, but whether maternal metabolic unbalance may affect brain plasticity of next generations is still unclear. Here, we demonstrate that maternal high fat diet (HFD)-dependent insulin resistance multigenerationally impairs synaptic plasticity, learning and memory. HFD downregulates BDNF and insulin signaling in maternal tissues and epigenetically inhibits BDNF expression in both germline and hippocampus of progeny. Notably, exposure of the HFD offspring to novel enriched environment restores *Bdnf* epigenetic activation in the male germline and counteracts the transmission of cognitive impairment to the next generations. BDNF administration to HFD-fed mothers or preserved insulin sensitivity in HFD-fed p66Shc KO mice also prevents the intergenerational transmission of brain damage to the progeny. Collectively, our data suggest that maternal diet multigenerationally impacts on descendants’ brain health via gametic mechanisms susceptible to lifestyle.

## Abstract (web summary)

It’s well known that hippocampal synaptic plasticity and memory are impaired in experimental models of metabolic diseases, however, it is unclear if maternal diet or metabolic alterations around the gestational age may multigenerationally affect learning and memory. In this study, authors demonstrate that maternal high fat diet-dependent insulin resistance affects synaptic plasticity and memory of descendants until the third generation via reduced exon specific brain-derived neurotrophic factor expression in the hippocampus of descendants

## Introduction

In the past years, growing attention has been devoted to the impact of overnutrition and metabolic diseases on brain health and function^1^. Epidemiological evidence indicate a higher risk of cognitive decline and neurodegenerative diseases in patients affected by obesity and type 2 diabetes^2,3^. Moreover, maternal obesity and consumption of high-fat diet (HFD) are associated with anxiety-like behavior and neurodevelopmental disorders in the offspring^4,5^. Strikingly, in experimental models HFD has been reported to transgenerationally predispose to obesity and metabolic syndrome until the third generation via an epigenetic inheritance^6^. This probably occurs because genes can retain memory of the early-life metabolic stress via epigenetic changes that include posttranslational modifications of histone proteins and DNA methylation^7^. In this regard, early-life stress may induce long-term neurobiological modifications affecting synaptic function and structural plasticity^8,9^.

Hippocampus is a brain area playing a critical role in learning and memory via changes in synaptic plasticity^10^ that is targeted by nutrient- and metabolic disease-related signals^11–13^. We recently reported that increased GluA1 S-palmitoylation underlies hippocampal synaptic plasticity impairment and cognitive decline observed in experimental models of metabolic diseases^14^. However, whether maternal diet or metabolic alterations around the gestational age may multigenerationally affect learning and memory is not yet known. Here we demonstrate that maternal HFD affects synaptic plasticity and memory of descendants until the third generation and reduces exon-specific brain-derived neurotrophic factor (*Bdnf*) expression. Accordingly, the early-life metabolic stress alters epigenetic markers on the promoters of *Bdnf* gene in both germline and hippocampus of HFD progeny. Exposure to novel enriched environment (NEE), a paradigm of physical and mental training, counteracts the multigenerational transmission of HFD detrimental effects by restoring both epigenetic modifications and BDNF levels in the hippocampus of progeny. Finally, our findings suggest that the intergenerational inhibition of neurotrophic factor expression and memory is triggered by alteration of both BDNF and insulin signaling in insulin-resistant mothers. Accordingly, BDNF administration or lack of pro-insulin resistance gene *p66Shc* in mothers abolishes the HFD-dependent transmission of cognitive impairment to the offspring.

## Results

### Maternal HFD impairs learning and memory in the offspring

**Fig. 1:**
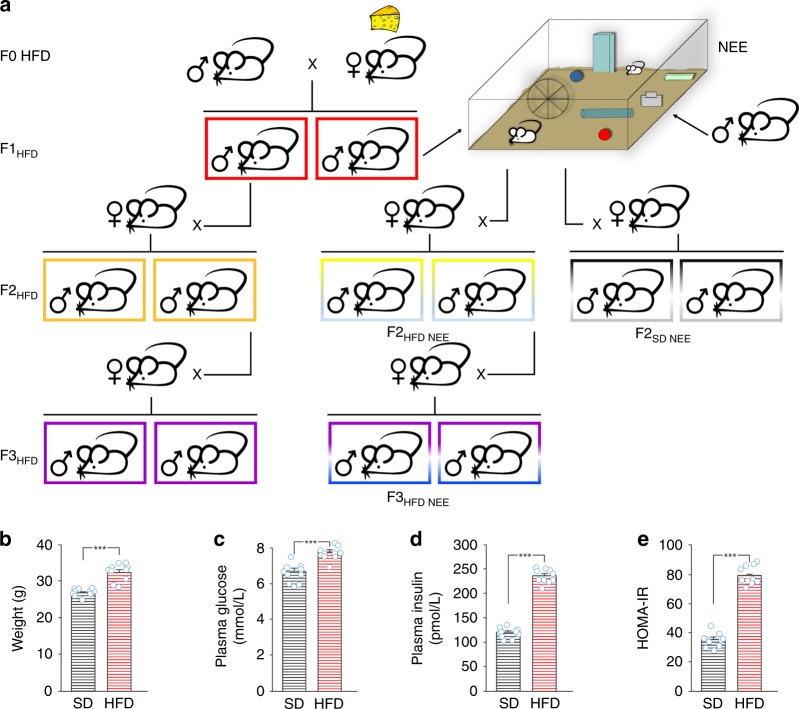
Experimental model. **a** Female mice (F0) were fed with either standard or high-fat diet (F0 SD and F0 HFD, respectively) for 4 weeks before mating with control males fed with SD. Unless otherwise specified, HFD was maintained during the pregnancy and until the second week of lactation. The offspring (F1_HFD_) and descendants (F2_HFD_ and F3_HFD_) were always fed with SD. F1_HFD_ and F2_HFD_ male mice were mated with control females to generate F2_HFD_ and F3_HFD_ mice, respectively. A subgroup of F1_HFD_ mice was exposed to novel enriched environment (NEE) for 4 weeks after the weaning. Subsequently, they were mated with control females to generate F2_HFD_ NEE mice. F2_HFD_ NEE male mice were mated with control females to generate F3_HFD_ NEE mice. **b** Weight, **c** fasting glucose plasma levels, **d** fasting insulin plasma levels, and **e** HOMA-IR score of SD and HFD female mice after 4 weeks of dietary regimen (*n* = 10 for each group; statistics by unpaired Student’s *t* test). Data are expressed as mean ± standard error of the mean (SEM). ***p < 0.001

Previous studies reported that maternal HFD affected hippocampal plasticity of the offspring by impairing adult neurogenesis, dendritic spine formation, and cognitive functions^15–17^. However, it is unknown whether brain function of the next generations may be impaired and/or epigenetically influenced by the dysmetabolic environment of the ancestor. To test this hypothesis, we fed female mice (named F0) with HFD for 4 weeks before mating, during the pregnancy, and until the second week of lactation (hereinafter referred as F0 HFD mother) and evaluated hippocampal-dependent synaptic plasticity and memory of the male descendants, hereinafter named F1_HFD_, F2_HFD_, and F3_HFD_ indicating the first, the second, and the third generations, respectively (Fig. 1a). Remarkably, in our experimental model the offspring of HFD mothers were consistently fed with standard diet (SD) and they were never exposed to HFD.

**Fig. 2:**
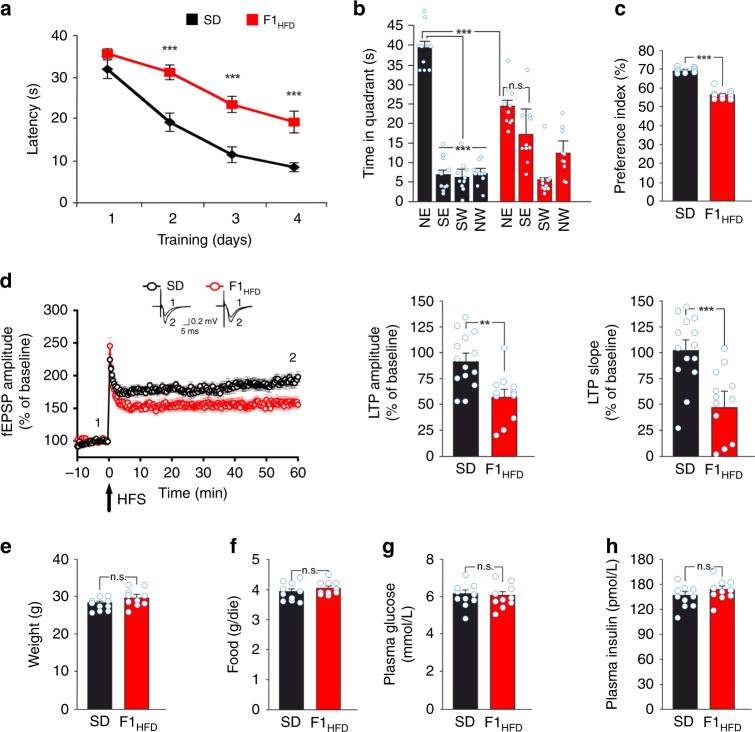
Maternal HFD impairs hippocampal synaptic plasticity, learning, and memory. **a** Latency to reach the hidden platform in the MWM test for SD and F1_HFD_ mice (*n* = 10 mice derived from 6 to 7 litters for each group; statistics by unpaired Student’s *t* test). **b** Time spent in the four quadrants during the probe test performed on day 5 of MWM. North–East (NE) is the quadrant where the platform was placed during the training (target quadrant) (*n* = 10 mice derived from 6 to 7 litters for each group; statistics by unpaired Student’s *t* test for time in the target quadrant and one-way ANOVA and Bonferroni post hoc for time in all quadrants). **c** Preference for the novel object in the NOR paradigm (*n* = 10 mice derived from 6 to 7 litters for each group; statistics by unpaired Student’s *t* test). **d** On the left, time course of LTP at CA3–CA1 synapses induced by HFS delivered at time 0 (arrow) in hippocampal slices obtained from SD mice (*n* = 14 slices from 5 mice of different litters) or F1_HFD_ (*n* = 12 slices from 4 mice of different litters). Results are expressed as percentages of baseline fEPSP amplitudes (=100%). Insets show representative fEPSPs at baseline (1) and during the last 5 min of LTP recordings (2). Traces are averages of 5 consecutive responses at the time points indicated with 1 and 2. On the right, bar graphs showing LTP assessed by measuring fEPSP amplitudes and slopes during the last 5 min in SD and F1_HFD_ mice (statistics by unpaired Student’s *t* test). **e** Weight, **f** average food consumption, **g** fasting glucose plasma levels, and **h** fasting insulin plasma levels of SD and F1_HFD_ mice at the time point of behavioral tests (*n* = 10 mice derived from 6 to 7 litters for each group; statistics by unpaired Student’s *t* test) after 2 fasting hours. Data are expressed as mean ± SEM. **p < 0.01; ***p < 0.001; n.s. not significant

We started studying the diet-dependent metabolic alterations in overfed female mice before mating. After 4 weeks of HFD, mice showed a moderate increase in both weight (32.5 ± 0.7 vs. 26.8 ± 0.3 g; *n* = 10, unpaired Student’s *t* test *p* = 8.9 × 10^−7^) and fasting plasma glucose levels (7.7 ± 0.2 vs. 6.6 ± 0.5 mmol L^−1^; *p* = 4.3 × 10^−5^) compared to controls (Fig. 1b, c). More importantly, they had higher levels of fasting plasma insulin (234.0 ± 4.9 vs. 118.7 ± 3.3 pmol L^−1^; unpaired Student’s *t* test *p* = 6.1 × 10^−14^) causing a significant increase of homeostatic model assessment of insulin resistance (HOMA-IR) score (80.0 ± 2.1 vs. 34.9 ± 1.6; *p* = 8.4 × 10^−13^) resembling a model of peripheral insulin resistance (Fig. 1d, e). Next, we investigated hippocampus-dependent learning and memory in the offspring of insulin-resistant mothers. In the Morris water maze (MWM), F1_HFD_ mice showed higher latency to reach the hidden platform starting from the second day of training (30.9 ± 1.8 vs. 19.0 ± 2.2 s for day 2; 23.3 ± 2.0 vs. 11.3 ± 1.9 s for day 3; 19.1 ± 2.6 vs. 8.3 ± 1.1 s for day 4; *n* = 10, unpaired Student’s *t* test *p* < 0.001 for each day; Fig. 2a).

During the probe test, they also spent less time than controls (i.e., mice born to SD-fed mice) in the target quadrant (time in the target quadrant: 24.6 ± 1.7 vs. 39.6 ± 1.8 s, unpaired Student’s *t* test *p* = 7.27 × 10^−6^; time in the four quadrants: North–East (NE) vs. South–East (SE), one-way analysis of variance (ANOVA) *p* = 0.078 for F1_HFD_; Fig. 2b). The F1_HFD_ mice also showed less preference than controls for the novel object in the novel object recognition (NOR) test (69.2 ± 0.4% vs. 56.5 ± 0.8%; *n* = 10, unpaired Student’s *t* test *p* = 3.55 × 10^−11^; Fig. 2c). Accordingly, we found a significant reduction of long-term potentiation (LTP) at the CA3–CA1 hippocampal synapses in slices from F1_HFD_ mice (field excitatory postsynaptic potential (fEPSP) amplitude potentiation: 57.9 ± 6.7% vs. 91.9 ± 7.2%, *p* = 0.0016; fEPSP slope: 52.6 ± 9.8% vs. 102.2 ± 9.2%, *p* = 0.0008; *n* = 14 for SD and *n* = 12 for HFD; unpaired Student’s *t* test Fig. 2d). Notably, weight, food consumption, glucose and insulin plasma levels, and insulin sensitivity were not significantly different between F1_HFD_ and SD mice (Fig. 2e–h and Supplementary Fig. 1a).

### HFD alters the cognitive functions of descendants

Male offspring of HFD mothers was crossed with control, SD-fed, females and their descendants were studied until the third generation that had no contacts with the dysmetabolic environment of the ancestor^18^. We chose to investigate the paternal inheritance because recent evidence pointed out the critical role of male germline in the multigenerational transmission of HFD effects^6,19^.

**Fig. 3:**
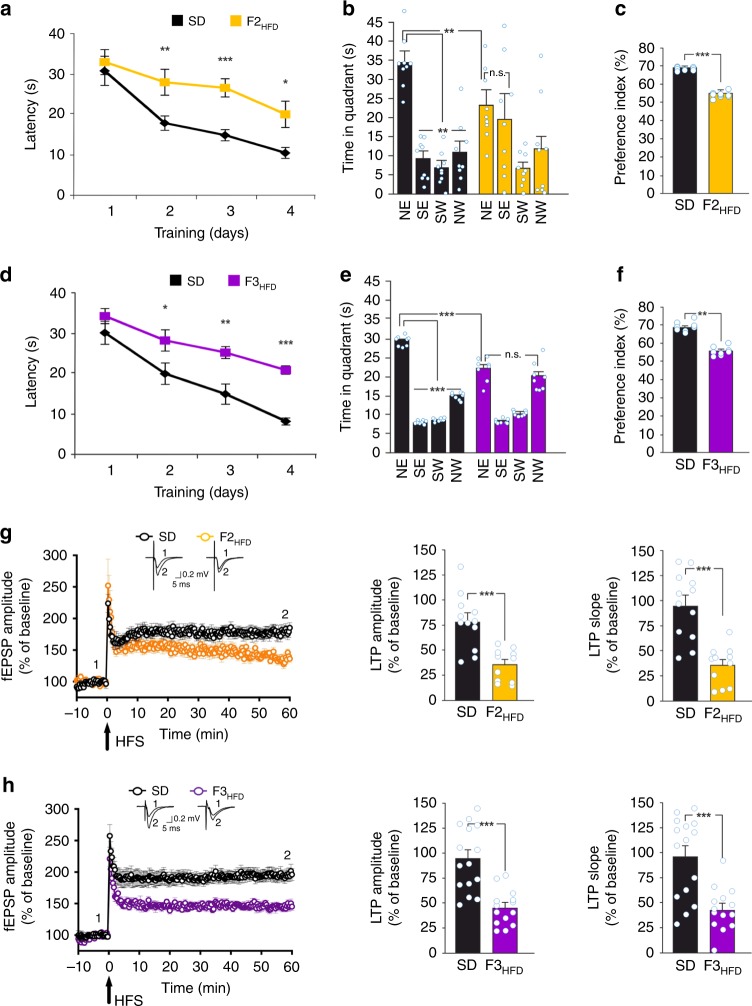
Progenitor’s HFD affects cognitive functions until the third generation. **a** Latency to reach the hidden platform in the MWM test for SD and F2_HFD_ mice (*n* = 9 mice from 6 litters for each group; statistics by unpaired Student’s *t* test). **b** Time spent in the four quadrants during the probe test by SD and F2_HFD_ mice. NE is the target quadrant (*n* = 9 mice from 6 litters for each group; statistics by unpaired Student’s *t* test for time in the target quadrant and one-way ANOVA and Bonferroni post hoc for time in all quadrants). **c** Preference index in the NOR paradigm for SD and F2_HFD_ mice (*n* = 9 mice from 6 litters for each group; statistics by unpaired Student’s *t* test). **d** Escape latency in MWM test for SD and F3_HFD_ mice (*n* = 8 for each group; statistics by unpaired Student’s *t* est). **e** Probe test of SD and F3_HFD_ mice (*n* = 8 mice from 6 litters for each group; statistics by unpaired Student’s *t* test for time in the target quadrant and one-way ANOVA and Bonferroni post hoc for time in all quadrants). **f** Preference index for SD and F3_HFD_ mice (*n* = 8 mice from 6 litters for each group; statistics by unpaired Student’s *t* test). **g** Time course of LTP at CA3–CA1 synapses and bar graphs showing changes in fEPSP amplitudes and slopes in SD (*n* = 12 slices from 4 mice of different litters) and F2_HFD_ (*n* = 11 slices from 4 mice of different litters) mice, as described in Fig. 2d (statistics by unpaired Student’s *t* test). **h** LTP at CA3–CA1 synapses in SD (*n* = 15 slices from 5 mice of different litters) and F3_HFD_ (*n* = 14 slices from 5 mice of different litters) mice (statistics by unpaired Student’s *t* test). Data are expressed as mean ± SEM. *p < 0.05; **p < 0.01; ***p < 0.001; n.s. not significant

F2_HFD_ and F3_HFD_ mice exhibited a cognitive impairment comparable to F1_HFD_ mice, as assessed by LTP and hippocampus-dependent learning and memory tasks. Specifically, in the MWM test both F2_HFD_ and F3_HFD_ mice took longer times to find the platform (F2_HFD_ and F3_HFD_: unpaired Student’s *t* test *p* < 0.05 from the day 2; *n* = 9 for F2 and *n* = 8 for F3; Fig. 3a, d) and explored the target quadrant less than controls (F2_HFD_: 22.9 ± 3.2 vs. 34.1 ± 2.4 s, *p* = 0.0097; F3_HFD_: 21.9 ± 1.1 vs. 29.3 ± 0.5 s, unpaired Student’s *t* test *p* *=* 1.6 × 10^−5^; Fig. 3b, e).

Similarly, in the NOR test both F2_HFD_ and F3_HFD_ mice showed lower preference index than controls (F2_HFD_: 54.6 ± 0.6% vs. 68.8 ± 0.3%, *p* = 1.6 × 10^−11^; F3_HFD_: 55.8 ± 0.9% vs. 68.7 ± 1.0%, unpaired Student’s *t* test *p* = 1.6 × 10^−8^; *n* = 9 for F2 and *n* = 8 for F3; Fig. 3c, f). The progeny of HFD mothers also exhibited a decrease in LTP similar to that observed in F1_HFD_ mice (F2_HFD_: fEPSP amplitude, 35.8 ± 5.1% vs. 78.3 ± 8.3%, *p* = 0.0002; fEPSP slope, 35.9 ± 6.0% vs. 94.6 ± 10.1%, *p* = 0.00005; *n* = 12 for SD and *n* = 11 for F2_HFD_; F3_HFD_: fEPSP amplitude, 45.7 ± 4.8% vs. 94.7 ± 8.6%, *p* = 2.9 × 10^−5^; fEPSP slope, 44 ± 6% vs. 96 ± 10.7%, *p* = 0.0002; *n* = 15 for SD and *n* = 14 for F3_HFD_; unpaired Student’s *t* test; Fig. 3g, h).

To investigate whether the multigenerational effects of HFD were sex specific, we also assessed the performance of F2_HFD_ and F3_HFD_ female mice in the NOR test and we found no significant differences with impairment of hippocampus-dependent memory observed in the male progeny (Supplementary Fig. 2a). Finally, to test whether the mothers’ overnutrition during lactation played a critical role in the intergenerational transmission of HFD detrimental effects, we set up another experimental model in which mothers were fed with HFD until the birth of pups, then they were switched to SD during lactation. The F1_HFD_ male mice were then mated with control females and their descendants, named F2_HFD NL_ (i.e., no lactation) and F3_HFD NL_ mice, were subjected to the NOR test. The cognitive impairment exhibited by the descendants of HFD mothers undergoing this shortened overnutrition protocol was not significantly different from what observed in our typical experimental model, thus suggesting that the detrimental effects of HFD are primarily exerted during pregnancy with no significant contribution of the lactation period (Supplementary Fig. 2b). Collectively, our findings suggest that maternal insulin resistance occurring in the critical phase of embryo development multigenerationally impairs brain functions in the adulthood.

### HFD multigenerationally reduces *Bdnf* expression

To gain insight into the mechanisms underlying the altered hippocampal plasticity in HFD mothers’ descendants, we analyzed the expression of a large number of plasticity-related genes in hippocampal extracts of F1_HFD_, F2_HFD_, and F3_HFD_ mice. Real-time PCR (RT-PCR) array revealed either upregulation or downregulation of several genes in all generations of F0 HFD descendants (Supplementary Table 1). Remarkably, the transcription of genes coding for the neurotrophic factors BDNF and nerve growth factor was reduced in F0 HFD progeny (Supplementary Fig. 3a). However, *Bdnf* was the only gene whose inhibition was statistically significant in all the three generations (F1_HFD_: −479%, F2_HFD_: −285%, F3_HFD_: −402%; *n* = 4; Supplementary Fig. 3a).

**Fig. 4:**
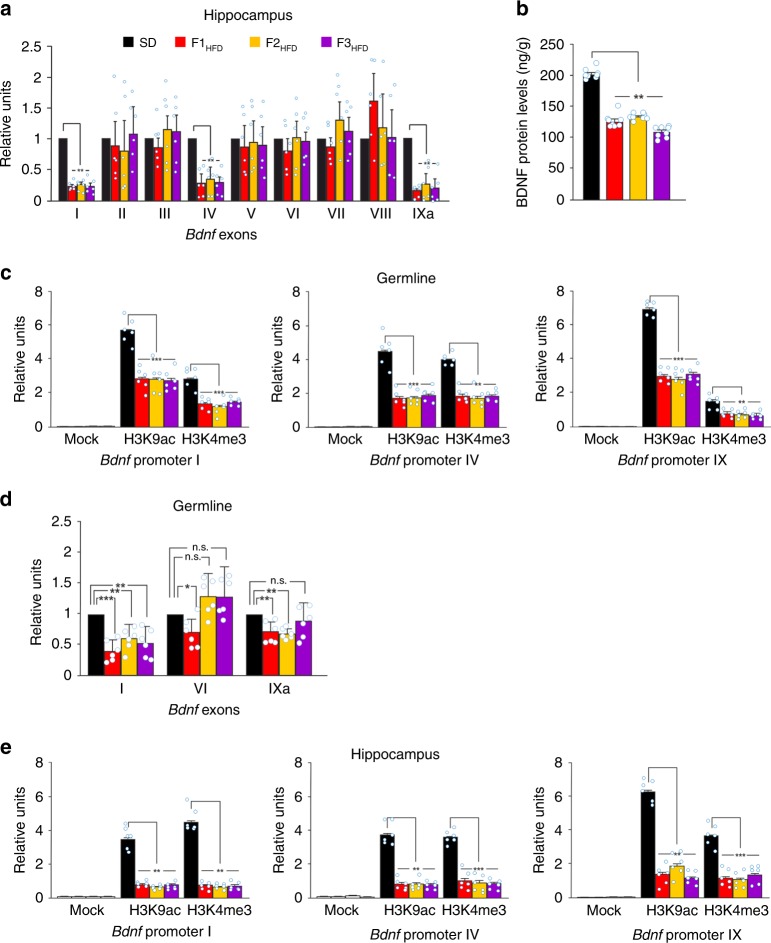
Progenitor’s HFD multigenerationally decreases *Bdnf* expression via epigenetic mechanisms. **a** Expression of *Bdnf* exons in the hippocampus of SD mice and F0 HFD descendants. Gene expression was normalized to actin. Data represent mean values obtained from six mice derived from five litters for each group; experiments were performed in triplicate (statistics by one-way ANOVA and Bonferroni post hoc). **b** BDNF levels in the hippocampus of SD mice and F0 HFD descendants. ELISA assay was performed in duplicate (*n* = 8 mice derived from 5 litters per group; statistics by one-way ANOVA and Bonferroni post hoc). **c** Chromatin immunoprecipitation (ChIP) assays of histone 3 lysine 9 acetylation (H3K9ac) and histone 3 lysine 4 trimethylation (H3K4me3) on the promoters I, IV, and IX of *Bdnf* gene in the germline of SD and F0 HFD descendant male mice. qPCR experiments were performed in triplicate (*n* = 6 mice derived from 5 litters for each group; statistics by one-way ANOVA and Bonferroni post hoc). **d** Bdnf exon expression in the germline of SD and F0 HFD descendant male mice. Exons II, III, IV, V, VII, and VIII were not detectable. Experiments were performed in triplicate (*n* = 6 mice derived from 5 litters for each group; statistics by one-way ANOVA and Bonferroni post hoc). **e** ChIP assays of H3K9ac and H3K4me3 on the promoters I, IV, and IX of *Bdnf* gene in the hippocampus of SD and F0 HFD descendant male mice. Experiments were performed in triplicate (*n* = 6 mice derived from 5 litters for each group; statistics by one-way ANOVA and Bonferroni post hoc). Data are expressed as mean ± SEM. *p < 0.05; **p < 0.01; ***p < 0.001; n.s. not significant

*Bdnf* gene has a complex structure in both humans and mice^20–22^ with multiple exons whose expression is regulated by different stimuli, including neuronal activity and stressful conditions^23–25^. We analyzed the expression of *Bdnf* transcripts in all generations and found a significant reduction of exons I, IV, and IXa in the hippocampus of all F0 HFD descendants (*F*_3.09_ = 30.22 for exon I, F1_HFD_ vs. SD *p* = 0.00028, F2_HFD_ vs. SD *p* = 0.00026, F3_HFD_ vs. SD *p* = 0.00012; *F*_3.09_ = 5.57 for exon IV, F1_HFD_ vs. SD *p* = 0.021, F2_HFD_ vs. SD *p* = 0.038, F3_HFD_ vs. SD *p* = 0.021; *F*_3.09_ = 21.29 for exon IXa, F1_HFD_ vs. SD *p* = 6.62 × 10^−8^, F2_HFD_ vs. SD *p* = 0.00038, F3_HFD_ vs. SD *p* = 0.0014; *n* = 6; one-way ANOVA; Fig. 4a). *Bdnf* expression was significantly downregulated in neurons, as indicated by single-cell analysis performed on mRNAs extracted from hippocampal CA1 neurons of SD and F1_HFD_ mice (−77%, unpaired Student’s *t* test *p* = 0.0016; *n* = 3; Supplementary Fig. 3b, c). Accordingly, BDNF protein levels were also decreased in hippocampi of F0 HFD progeny (*F*_2.94_ = 157.59, F1_HFD_ vs. SD *p* = 5.15 × 10^−10^, F2_HFD_ vs. SD *p* = 8.28 × 10^−10^, F3_HFD_ vs. SD *p* = 3.27 × 10^−11^; *n* = 8; one-way ANOVA; Fig. 4b) and independently of the dietary regimen of F1 pups during lactation (Supplementary Fig. 2c). The multigenerational effect of maternal overnutrition on *Bdnf* expression could be due to the recurrence of metabolism-related humoral alterations in each generation. To address this issue, we first analyzed weight, food consumption, plasma glucose and insulin levels, and insulin sensitivity in F2_HFD_ and F3_HFD_ mice, and we did not find significant changes compared to SD mice (Supplementary Fig. 2d–h). All F0 HFD descendants also showed locomotor activity comparable with control mice (Supplementary Fig. 2i).

Later we tested the intriguing hypothesis that the multigenerational reduction of *Bdnf* expression depended on epigenetic inhibition occurring in both germline and hippocampus of all descendants. Expression of *Bdnf* exons is finely regulated by epigenetic changes on multiple regulatory sequences^26,27^. Specifically, lysine 9 acetylation (H3K9ac) and lysine 4 trimethylation (H3K4me3) on histone 3 tail promote exon transcription. Therefore, we studied these epigenetic markers on the promoter of exons I, IV, and IXa in the male gonads of F0 HFD progeny. H3K9ac and H3K4me3 on the *Bdnf* promoters were critically reduced in the germline of F0 HFD descendants (promoter I: *F*_3.09_ = 31.83 for H3K9ac and *F*_3.09_ = 22.73 for H3K4me3, *p* < 0.001 for SD vs. all HFD generations; promoter IV: *F*_3.09_ = 31.64 for H3K9ac, *p* < 0.001 for SD vs. all HFD generations and *F*_3.09_ = 15.85 for H3K4me3, *p* < 0.01 for SD vs. all HFD generations; promoter IX: *F*_3.09_ = 23.52 for H3K9ac, *p* < 0.001 for SD vs. all HFD generations and *F*_3.09_ = 13.19 for H3K4me3, *p* < 0.01 for SD vs. all HFD generations; *n* = 6; one-way ANOVA; Fig. 4c). Accordingly, the expression of *Bdnf* exon I and XIa were significantly inhibited in the male germline of HFD progeny (exon I: F1_HFD_ = −61%, F2_HFD_ = −40%, F3_HFD_ = −48%; exon XIa: F1_HFD_ = −39%, F2_HFD_ = −33%; *p* < 0.01 for all groups vs. SD; one-way ANOVA; Fig. 4d). Moreover, mRNA expression of BDNF coding exon IX appeared reduced in the germline as well as in gastrocnemius muscle and heart of F1_HFD_ mice (−30; −57 and −33%, respectively; Supplementary Fig. 3d). More importantly, plasma BDNF protein levels were also significantly reduced in all HFD progeny (Supplementary Fig. 3e).

The *Bdnf* regulatory sequences were also epigenetically inhibited in the hippocampi of these mice (promoter I: *F*_3.09_ = 27.75 for H3K9ac and *F*_3.09_ = 29.53 for H3K4me3, *p* < 0.01 for SD vs. all HFD generations; promoter IV: *F*_3.09_ = 15.69 for H3K9ac, *p* < 0.01 for SD vs. all HFD generations and *F*_3.09_ = 35.69 for H3K4me3, *p* < 0.001 for SD vs. all HFD generations; promoter IX: *F*_3.09_ = 20.65 for H3K9ac, *p* < 0.01 for SD vs. all HFD generations and *F*_3.09_ = 37.84 for H3K4me3, *p* < 0.001 for SD vs. all HFD generations; *n* = 6; one-way ANOVA Fig. 4e), whereas both H3K9ac and H3K4me3 were not significantly modified on the promoters III and VI of *Bdnf* gene (Supplementary Fig. 3f).

Moreover, to investigate whether the intergenerational effects of HFD were mediated by changes in maternal behavior and to differentiate between gametic and somatic transmission of the phenotype, we performed cross-fostering (CF) and in vitro fertilization (IVF) experiments. Both F1_HFD_ mice fostered by control females (hereinafter named F1_HFD_ CF) and mice generated by fertilizing control oocytes with sperm of F1_HFD_ mice (hereinafter named F2_HFD_ IVF) showed behavioral and molecular alteration similar to F1_HFD_ animals raised by F0 HFD mothers (Supplementary Fig. 4a–d, e–h). Collectively, our data demonstrated that progenitor’s HFD intergenerationally downregulated BDNF at multi-organ level by epigenetically inhibiting the expression of the neurotrophic factor through a gametic mechanism.

### NEE blocks the multigenerational transmission of HFD effects

**Fig. 5:**
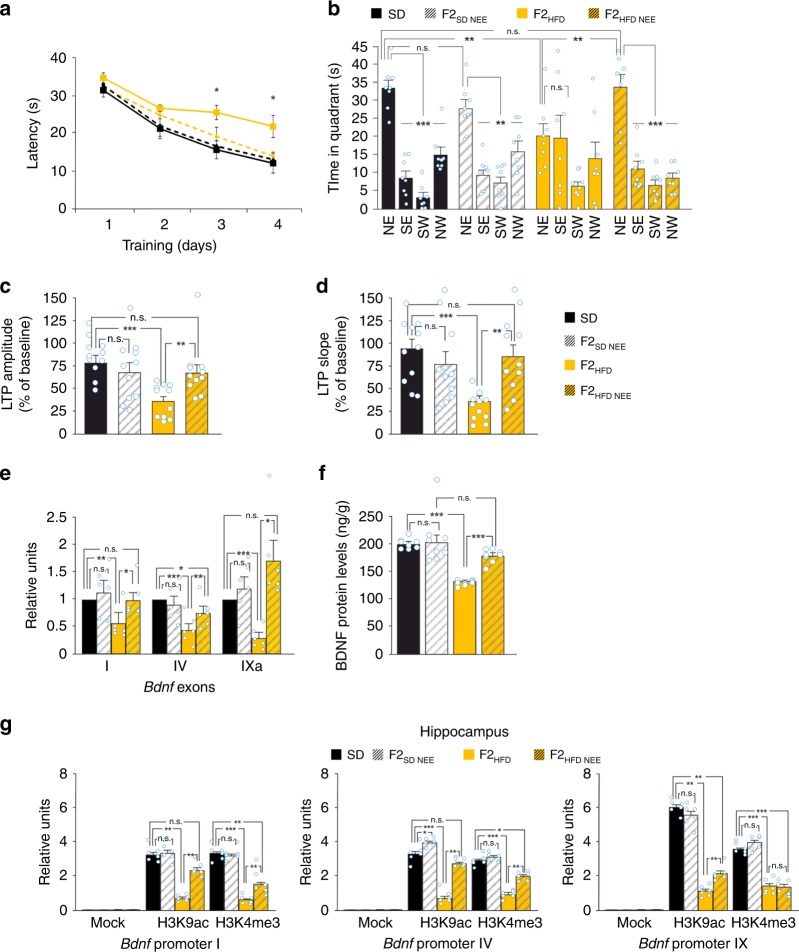
NEE blocks the multigenerational transmission of HFD-dependent cognitive impairment. **a** Latency to reach the hidden platform in the MWM test for SD, F2_SD_ NEE, F2_HFD_, and F2_HFD_ NEE mice (see Fig. 1 for the experimental design; *n* = 8 mice derived from 6 litters for each group; significance is indicated between F2_HFD_ and F2_HFD_ NEE mice; statistics by two-way ANOVA and Bonferroni post hoc). **b** Time spent in the four quadrants during the probe test of MWM by SD, F2_SD_ NEE, F2_HFD_, and F2_HFD_ NEE mice. NE is the target quadrant (*n* = 8 mice derived from 6 litters for each group; statistics by two-way ANOVA and Bonferroni post hoc). **c** Bar graphs showing changes in fEPSP amplitudes and **d** slopes in SD, F2_SD_ NEE, F2_HFD_, and F2_HFD_ NEE mice (*n* = 11 slices from 4 mice of different litters per each group; statistics by two-way ANOVA and Bonferroni post hoc). **e** *Bdnf* exon I, IV, and IXa expression (normalized to actin) in the hippocampus of SD, F2_SD_ NEE, F2_HFD_, and F2_HFD_ NEE mice. Data represent mean values obtained from six mice derived from five litters for each group; experiments were performed in triplicate (statistics by two-way ANOVA and Bonferroni post hoc). **f** BDNF levels in the hippocampus of SD, F2_SD_ NEE, F2_HFD_, and F2_HFD_ NEE mice. ELISA assay was performed in duplicate (*n* = 8 mice derived from 5 litters per group; statistics by two-way ANOVA and Bonferroni post hoc). **g** ChIP assays of H3K9ac and H3K4me3 on the promoters I, IV, and IX of *Bdnf* gene in the hippocampus of SD, F2_SD_ NEE, F2_HFD_, and F2_HFD_ NEE mice. Data represent mean values obtained from 6 mice derived from 4 to 5 litters for each group; qPCR experiments were performed in triplicate (statistics by two-way ANOVA and Bonferroni post hoc). Data are expressed as mean ± SEM. *p < 0.05; **p < 0.01; ***p < 0.001; n.s. not significant

Lifestyle (e.g., stress, social interaction, diet) has been shown to influence cognitive functions^28–30^. In particular, NEE has been proposed to counteract the detrimental effects of HFD on brain health^31^. We investigated the possibility to break the transgenerational transmission of cognitive impairment due to the progenitor’s overnutrition by exposing F1_HFD_ male mice to NEE (F1_HFD_ NEE) for 4 weeks before mating (Fig. 1a). We then studied the second generation (F2_HFD NEE_ mice), sharing with F2_HFD_ mice the same HFD ancestor but being descendant of F1_HFD_ males grown in NEE. The cognitive performance of F2_HFD NEE_ mice was compared with those of F2_HFD_, SD, and F2_SD NEE_ mice born from SD mice exposed to NEE. In SD descendants, paternal exposure to NEE did not significantly change learning, memory, and hippocampal synaptic plasticity compared to controls (time in the target quadrant: *F*_3.07_ = 5.2, F2_SD NEE_ vs. SD, *p* = 0.093; fEPSP amplitude: *F*_2.92_ = 4.78, F2_SD NEE_ vs. SD, *p* = 0.44; two-way ANOVA). Interestingly enough, F2_HFD NEE_ mice showed an almost complete rescue of cognitive functions assessed by the MWM (day 4: *F*_3.07_ = 6.74, F2_HFD NEE_ vs. F2_HFD_, *p* = 0.034; time in the target quadrant: *F*_3.07_ = 4.78, F2_HFD NEE_ vs. F2_HFD_, *p* = 0.0098; *n* = 8; two-way ANOVA; Fig. 5a, b). Accordingly, LTP of F2_HFD NEE_ mice was significantly higher than that observed in F2_HFD_ mice (fEPSP amplitude: 67.6 ± 9.6% vs. 36.7 ± 5.3%, *F*_2.92_ = 5.62, *p* = 0.009; fEPSP slope: 87.5 ± 13.7% vs. 41 ± 5.9%, *F*_2.92_ = 6.54, *p* = 0.005, *n* = 11; two-way ANOVA; Fig. 5c, d).

We also found that *Bdnf* expression in hippocampi of F2_HFD NEE_ mice was significantly higher than that of F2_HFD_ mice at both mRNA (*F*_3.28_ = 9.27 for exon I, *p* = 0.014; *F*_3.28_ = 20.72 for exon IV, *p* = 0.0072; *F*_3.28_ = 7.57 for exon IXa, *p* = 0.019; *n* = 6; two-way ANOVA; Fig. 5e) and protein levels (*F*_3.07_ = 24.45, *p* = 1.28 × 10^−6^, *n* = 8; two-way ANOVA; Fig. 5f). NEE has been demonstrated to regulate *Bdnf* expression via epigenetic changes on *loci* closely related to those we studied^32^. We therefore analyzed H3K9ac and H3K4me3 on *Bdnf* promoters I, IV, and IX in hippocampal extracts of F2_HFD_ NEE mice and found an almost complete rescue of histone epigenetic activation on the regulatory sequences of exons I and IV (promoter I: *F*_3.28_ = 8.09 for H3K9ac, F2_HFD NEE_ vs. F2_HFD_ *p* = 0.0085, SD vs. F2_HFD NEE_ *p* = 0.18, *F*_3.28_ = 26.81 for H3K4me3, F2_HFD NEE_ vs. F2_HFD_ *p* = 0.0093, SD vs. F2_HFD NEE_ *p* = 0.0034; promoter IV: *F*_3.28_ = 18.15 for H3K9ac, F2_HFD NEE_ vs. F2_HFD_ *p* = 0.016, SD vs. F2_HFD NEE_ *p* = 0.73, *F*_3.28_ = 26.2 for H3K4me3, F2_HFD NEE_ vs. F2_HFD_ *p* = 0.0034, SD vs. F2_HFD NEE_ *p* = 0.031; promoter IX: *F*_3.28_ = 35.56 for H3K9ac, F2_HFD NEE_ vs. F2_HFD_ *p* = 0.0015, SD vs. F2_HFD NEE_ *p* = 0.0028, *F*_3.28_ = 23.06 for H3K4me3, F2_HFD NEE_ vs. F2_HFD_ *p* = 0.99, SD vs. F2_HFD NEE_ *p* = 0.00031; *n* = 6; two-way ANOVA; Fig. 5g). To determine whether NEE induced epigenetic changes on the germline of F1_HFD_ mice, we analyzed both H3K9ac and H3K4me3 on *Bdnf* regulatory sequences before and after exposure to NEE. We found significant changes of these epigenetic marks on *Bdnf* promoters I and IV in the germline of F1_HFD_ NEE mice compared to F1_HFD_ animals (promoter I: *F*_3.28_ = 13.29 for H3K9ac, *p* = 0.0034, *F*_3.28_ = 11.96 for H3K4me3, *p* = 0.015; promoter IV: *F*_3.28_ = 11.32 for H3K9ac, *p* = 0.0069, *F*_3.28_ = 9.12 for H3K4me3, *p* = 0.014; *n* = 6; two-way ANOVA; Supplementary Fig. 5a). Accordingly, we detected NEE-dependent rescue of learning and memory in F3_HFD NEE_ mice similar to what observed in F2_HFD NEE_ generation (day 4 of MWM: *F*_3.63_ = 36.69, F3_HFD NEE_ vs. F3_HFD_ *p* = 8.43 × 10^−5^; time in target quadrant: *F*_3.63_ = 28.88, F3_HFD NEE_ vs. F3_HFD_ *p* = 1.66 × 10^−4^; *n* = 9; two-way ANOVA; Supplementary Fig. 5b). Collectively, our findings suggested that both maternal HFD and paternal exposure to NEE multigenerationally influenced cognitive functions of F0 HFD descendants and regulated *Bdnf* expression via common epigenetic mechanisms.

### HFD affects histone acetyl-transferase/histone deacetylase (HAT/HDAC) binding to *Bdnf* promoters in the ovaries

**Fig. 6:**
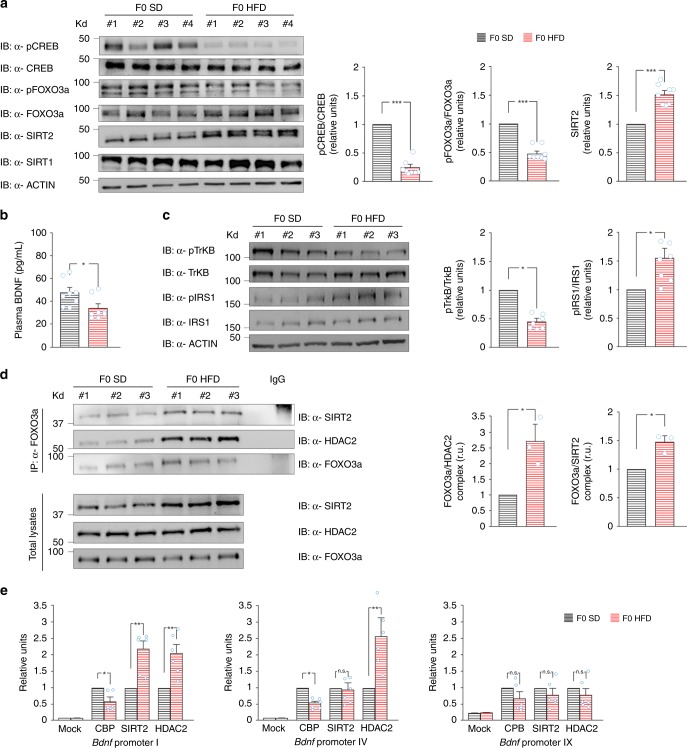
HFD affects HAT/HDAC recruitment on *Bdnf* promoters in ovaries. **a** Immunoblots and bar graphs of both CREB^Ser133^ and FOXO3a^Ser256^ phosphorylation and sirtuin expression in the ovaries from F0 SD and F0 HFD female mice (*n* = 8 per group; statistics by unpaired Student’s *t* test). Samples were harvested from two independent experiments. **b** BDNF plasma levels of F0 SD and F0 HFD female mice measured by ELISA performed in duplicate (*n* = 8 mice per group; statistics by unpaired Student’s *t* test). **c** Immunoblots (left) and bar graphs (right) of both TrkB^Tyr816^ and IRS1^Ser612^ phosphorylation in the ovaries from F0 SD and F0 HFD mothers (*n* = 7 per group; statistics by Mann–Whitney test). Samples were harvested from two independent experiments. **d** Immunoblots (left) and densitometry (right) of FOXO3a interaction with both SIRT2 (top) and HDAC2 (middle) in the ovaries from F0 SD and F0 HFD mice (*n* = 3 per group; statistics by Mann–Whitney test). At the bottom, tissue lysates probed with α-SIRT2, α-HDAC2, and α-FOXO3a. **e** ChIP assays of CBP, SIRT2, and HDAC2 binding on the promoters I, IV, and IX of *Bdnf* gene in the gonads of F0 SD and F0 HFD female mice. Data represent mean values obtained from six mice for each group; qPCR experiments were performed in triplicate (statistics by Mann–Whitney test). Data are expressed as mean ± SEM. Source data are provided as a Source Data file. *p < 0.05; **p < 0.01; ***p < 0.001; n.s. not significant

To understand the molecular events triggering the intergenerational transmission of HFD-dependent cognitive impairment, we investigated the expression and activation of the key nutrient sensors cAMP response element binding (CREB) and forkhead box protein O3a (FOXO3a) in the ovaries of mothers after 4 weeks of HFD. Both transcription factors CREB and FOXO3a were hypophosphorylated (−76.5%, *p* = 6.36 × 10^−5^ and −52.3%, *p* = 0.004, respectively; *n* = 8; unpaired Student’s *t* test) in the female gonads upon HFD, leading to CREB inhibition and FOXO3a activation (Fig. 6a). A common molecular cascade impinging on both CREB and FOXO transcriptional activity is the BDNF/Tropomyosin receptor kinase B (TrkB) signaling^33,34^. Therefore, we measured plasma BDNF levels and TrkB receptor activation in the ovaries of HFD-fed female mice before mating. Strikingly, we found both lower plasma BDNF levels (33.9 ± 3.8 vs. 47.6 ± 4.4 pg mL^−1^, *p* = 0.025; *n* = 8; unpaired Student’s *t* test; Fig. 6b) and significant reduction of ovarian TrkB phosphorylation in insulin-resistant mothers compared to controls (−55.2%, *n* = 7; Fig. 6c). Dephosphorylation of FOXO transcription factors may be due to alteration of insulin signaling in the tissues^35^. Accordingly, we found higher levels of inhibitory phosphorylation of insulin receptor substrate 1 (pIRS1^Ser612^), a marker of insulin resistance, in the ovaries of HFD-fed females (+54.7%, *n* = 7; Fig. 6c). Since FOXO3a dephosphorylation promotes nuclear translocation of this transcription factor and may affect its interaction with chromatin remodelers, we investigated its binding with HDAC2 and SIRT2 in the ovaries. HFD females showed higher levels of protein complexes FOXO3a/SIRT2 and FOXO3a/HDAC2 in the gonads (*n* = 3; Fig. 6d). Finally, we analyzed the binding of chromatin remodelers interacting with CREB and FOXO3a on the regulatory sequences of *Bdnf* gene in the ovaries. We found lower levels of acetyl transferase CREB-binding protein (CBP) on *Bdnf* promoters I and IV (−43 and −46%, respectively; *n* = 6, *p* < 0.05 for each promoter) and higher binding of histone deacetylases HDAC2 and SIRT2 on promoters I and IV in the gonads of F0 HFD female mice (promoter I: SIRT2 +118%, HDAC2 +105%, *p* < 0.01; promoter IV: HDAC2 +156%, Mann–Whitney test *p* < 0.01; Fig. 6e).

### BDNF counteracts the intergenerational effects of HFD

**Fig. 7:**
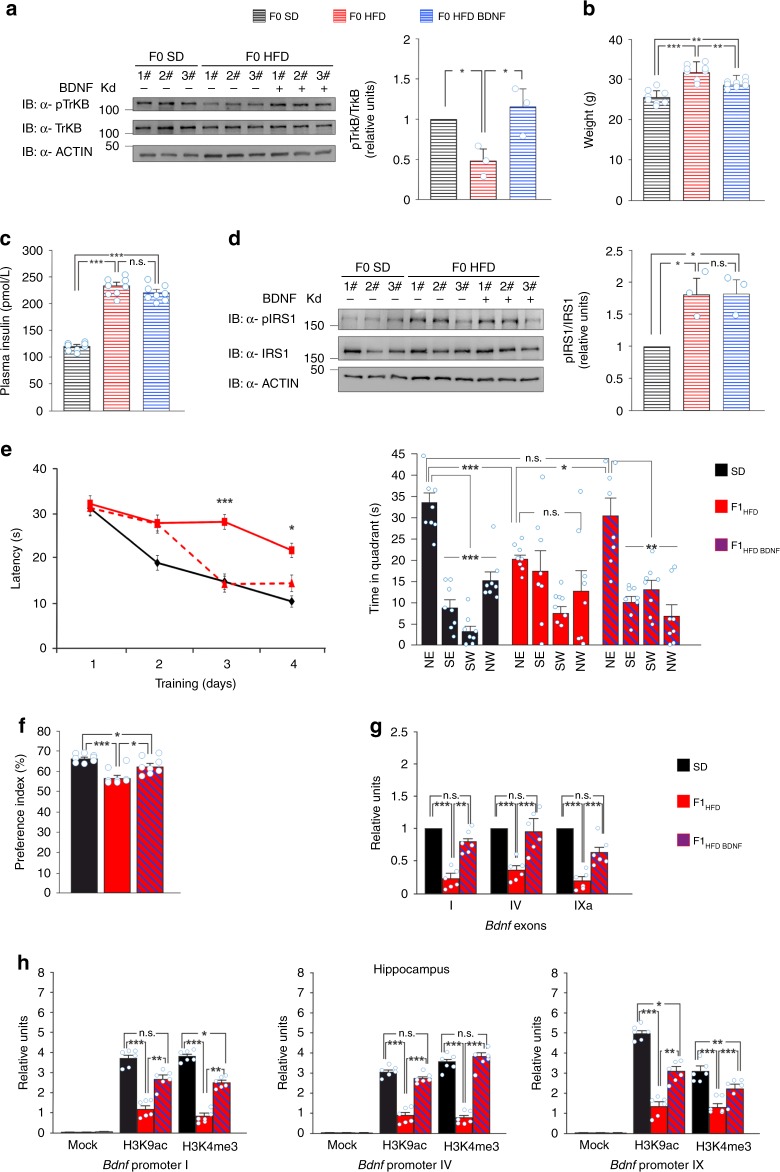
Maternal BDNF administration counteracts the effect of HFD on offspring’s cognitive function. **a** Immunoblots and bar graphs of TrkB^Tyr816^ phosphorylation in the ovaries of females fed with SD or HFD and intraperitoneally injected with vehicle or BDNF (F0 SD, F0 HFD, F0 HFD BDNF; *n* = 3 mice per group; statistics by two-way ANOVA and Bonferroni post hoc). **b** Weight and **c** plasma insulin after 4 weeks of treatment and calorie intake of F0 SD, F0 HFD, and F0 HFD BDNF female mice (*n* = 8 mice per group; statistics by two-way ANOVA and Bonferroni post hoc). **d** Immunoblots and bar graphs of IRS1^Ser612^ phosphorylation in the ovaries of F0 SD, F0 HFD, and F0 HFD BDNF mice (*n* = 3 mice per group; statistics by two-way ANOVA and Bonferroni post hoc). Source data are provided as a Source Data file. **e** Latency (left) and time spent during the probe test (right) in the MWM test for SD, F1_HFD_, and F1_HFD BDNF_ mice (*n* = 8 mice derived from 5 litters for each group; significance is indicated between F1_HFD_ and F1_HFD BDNF_ mice; statistics by two-way ANOVA and Bonferroni post hoc). **f** Preference index of SD, F1_HFD_, and F1_HFD BDNF_ mice in NOR test (*n* = 8 mice derived from 5 litters for each group; statistics by two-way ANOVA and Bonferroni post hoc). **g** *Bdnf* exon I, IV, and IXa expression (normalized to a*ctin*) in the hippocampus of SD, F1_HFD_, and F1_HFD BDNF_ mice. Data represent mean values obtained from six mice derived from four litters for each group; experiments were performed in triplicate (statistics by two-way ANOVA and Bonferroni post hoc). **h** ChIP assays of H3K9ac and H3K4me3 on the promoters I, IV, and IX of *Bdnf* gene in the hippocampus of SD, F1_HFD_, and F1_HFD BDNF_ mice. Data represent mean values obtained from six mice derived from four litters for each group; qPCR experiments were performed in triplicate (statistics by two-way ANOVA and Bonferroni post hoc). Data are expressed as mean ± SEM. *p < 0.05; **p < 0.01; ***p < 0.001; n.s. not significant

To dip inside the critical role of BDNF signaling alteration in HFD-dependent mother to offspring transmission of cognitive impairment, we intraperitoneally (IP) injected HFD-fed female mice with BDNF (3 times per week for 4 weeks) as long as they fed HFD until the breeding (hereinafter named F0 HFD BDNF). As expected, ovarian TrkB^Tyr816^ phosphorylation in F0 HFD BDNF mothers was similar to control females and significantly higher than that observed in F0 HFD dams (*F*_6.94_ = 10.03, F0 HFD BDNF vs. F0 HFD *p* = 0.032, F0 HFD BDNF vs. SD *p* = 0.51; *n* = 3; two-way ANOVA; Fig. 7a). BDNF administration exerted anorectic effects on HFD-fed mice, as indicated by both reduced weight gain and calorie intake of F0 HFD BDNF females (Fig. 7b and Supplementary Fig. 6a). However, administration of the neurotrophic factor did not significantly change the peripheral insulin resistance, as shown by the plasma levels of insulin, glycemia, and HOMA index (Fig. 7c and Supplementary Fig. 6a), nor did it rescue the hyperphosphorylation of ovarian IRS1^Ser612^ (Fig. 7d). More importantly, the offspring of F0 HFD BDNF females (i.e., F1_HFD BDNF_ mice) showed cognitive performances significantly higher than F1_HFD_ animals and comparable to controls when evaluated in MWM (day 4: *F*_3.63_ = 10.79, F1_HFD BDNF_ vs. F1_HFD_ *p* = 0.035, F1_HFD BDNF_ vs. SD *p* = 0.15; time in target quadrant: F1_HFD BDNF_ vs. F1_HFD_ *p* = 0.035, F1_HFD BDNF_ vs. SD *p* = 0.15; *n* = 8; two-way ANOVA; Fig. 7e). Moreover, they showed a greater preference index than F1_HFD_ mice (*F*_3.63_ = 13.92, F1_HFD BDNF_ vs. F1_HFD_ *p* = 0.012, F1_HFD BDNF_ vs. SD *p* = 0.032; *n* = 8; two-way ANOVA; Fig. 7f). We also found an almost complete rescue of *Bdnf* exon I, IV, and IXa expression in the hippocampus of F1_HFD BDNF_ mice (*F*_4.1_ = 40.07 for exon I, F1_HFD BDNF_ vs. F1_HFD_ *p* = 0.0018; *F*_4.1_ = 17.97 for exon IV, F1_HFD BDNF_ vs. F1_HFD_ *p* = 0.00039; *F*_4.1_ = 49.23 for exon IXa, F1_HFD BDNF_ vs. F1_HFD_ *p* = 0.00067; *n* = 6; two-way ANOVA; Fig. 7g). Accordingly, epigenetic marker activation on the regulatory sequences of *Bdnf* gene were higher in the hippocampus of F1_HFD BDNF_ mice compared to that in F1_HFD_ (promoter I: *F*_4.1_ = 14 for H3K9ac, *p* = 0.0051, *F*_4.1_ = 28.75 for H3K4me3, *p* = 0.0013; promoter IV: *F*_4.1_ = 74.19 for H3K9ac, *p* = 0.00011, *F*_4.1_ = 43.49 for H3K4me3, *p* = 0.00045; promoter IX: *F*_4.1_ = 13.71 for H3K9ac, *p* = 0.0074, *F*_4.1_ = 70.99 for H3K4me3, *p* = 6.2 × 10^−5^; *n* = 6; two-way ANOVA; Fig. 7h). Collectively, our findings reveal a critical role of BDNF signaling in the mother to offspring transmission of HFD-dependent cognitive deficits.

### p66Shc deficiency abolishes HFD intergenerational effects

To investigate the causative role of maternal insulin resistance in the intergenerational transmission of HFD-related changes of brain function, we used *p66Shc* knockout (KO) mice. p66Shc is one of the three isoforms of the adaptor protein family ShcA mediating insulin sensitivity in tissues^36^. Ranieri et al. found that *p66Shc* deficiency induced a protective effect in lepOb/Ob mice, an established genetic model of obesity and insulin resistance without affecting (hyper)insulinemia^37^. More importantly, their findings demonstrated that p66Shc interacted with IRS1 and promoted its inhibitory phosphorylation in white fat. Given this background, we hypothesized that *p66Shc* deficiency might preserve the insulin sensitivity in maternal tissues and counteract the intergenerational transmission of cognitive impairment.

**Fig. 8:**
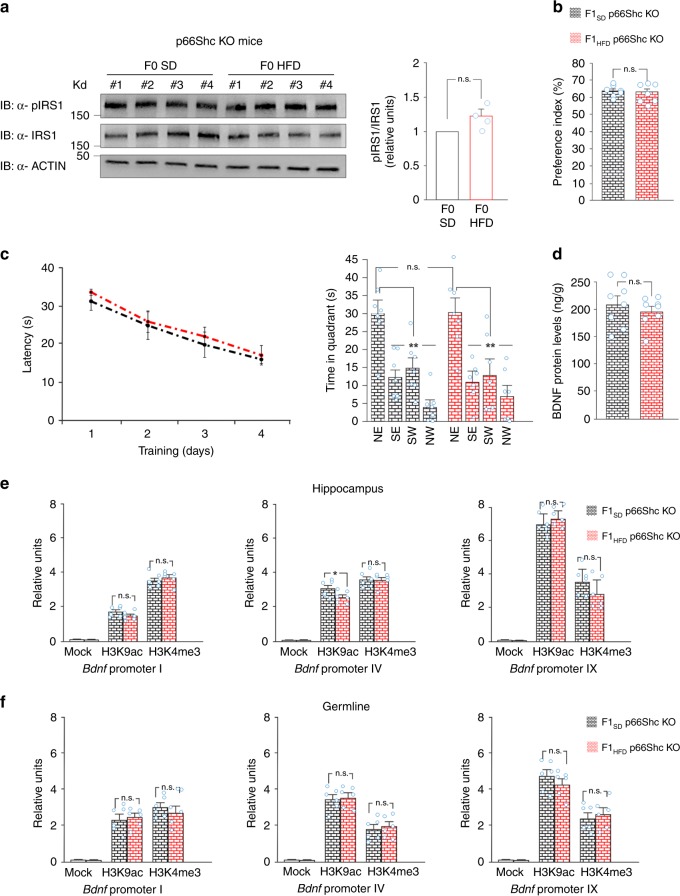
p66Shc deficiency abolishes the maternal HFD-dependent effects on F1 cognitive functions. **a** Immunoblots and bar graphs of IRS1^Ser612^ phosphorylation in the ovaries of *p66Shc* KO females fed with SD or HFD (*n* = 4 mice per group; statistics by unpaired Student’s *t* test). Source data are provided as a Source Data file. **b** Preference index of mice born from SD-fed or HFD-fed *p66Shc* KO (F1_SD_ p66Shc KO and F1_HFD_ p66Shc KO, respectively) (*n* = 7 mice derived from 5 litters per group; statistics by unpaired Student’s *t* test). **c** Latency to reach the platform (left) and time spent in the four quadrants during the probe test (right) in the MWM test for F1_SD_ p66Shc KO and F1_HFD_ p66Shc KO mice (*n* = 7 mice derived from 5 litters for each group; statistics by unpaired Student’s *t* test). **d** BDNF levels in the hippocampus of F1_SD_ and F1_HFD_ p66Shc KO mice. ELISA assay was performed in duplicate (*n* = 8 mice derived from 5 litters per group; statistics by unpaired Student’s *t* test). **e** ChIP assays of H3K9ac and H3K4me3 on the promoters I, IV, and IX of *Bdnf* gene in the hippocampus and **f** germline of F1_SD_ p66Shc KO and F1_HFD_ p66Shc KO mice. Data represent mean values obtained from six mice derived from four litters for each group; (statistics by unpaired Student’s *t* test). Data are expressed as mean ± SEM. *p < 0.05; **p < 0.01; n.s. not significant

First, we confirmed that p66Shc was expressed in the ovaries of wild-type females and deleted in KO mice (Supplementary Fig. 7a). HFD-fed *p66Shc* KO females showed increases of weight and plasma insulin levels resembling the insulin resistance phenotype observed in wild-type mice (Supplementary Fig. 7b, c). Nevertheless, no significant changes of IRS1^Ser612^ phosphorylation were detected in their ovaries after 4 weeks of HFD dietary regimen compared to SD mice (Fig. 8a). Strikingly, the offspring generated from HFD-fed *p66Shc* KO females (hereinafter named F1_HFD_ *p66Shc* KO) showed learning and memory comparable to those of mice born from SD-fed females (Fig. 8b, c). Accordingly, LTP was not significantly different between F1_HFD_ and SD *p66Shc* KO mice (fEPSP amplitude: 55.6 ± 13.1% vs. 46 ± 5.4%, *p* = 0.46; fEPSP slope: 59.7 ± 12.2% vs. 45.2 ± 4.8%, *p* = 0.23, *n* = 10–12 slices for each group; unpaired Student’s *t* test). Remarkably, BDNF levels were comparable in the hippocampus of both experimental groups (Fig. 8d). Finally, we did not find significant changes of epigenetic markers H3K9ac and H3K4me3 on *Bdnf* promoters I, IV, and IX between F1_HFD_ *p66Shc* KO and SD mice in both hippocampus and germline (Fig. 8e, f). Collectively, our data suggest that insulin signaling dysregulation contribute to trigger the HFD-dependent intergenerational effects on cognitive functions.

## Discussion

HFD-induced insulin resistance affects synaptic plasticity, learning, and memory^14^. Nutrient availability also impacts on the developing brain and it is now recognized that early-life dietary experience influences brain function in adult offspring^38^. However, whether metabolic factors may transgenerationally affect the cognitive function and the underlying molecular mechanisms remain largely unknown.

It is now clearly emerging that some epigenetic modifications can be inherited over generations and have a role in mediating the susceptibility to various diseases^39,40^. Here we show that maternal HFD multigenerationally impairs synaptic plasticity, learning, and memory via gametic mechanisms involving epigenetic inhibition of exon-specific *Bdnf* expression in the hippocampus of descendants.

We set up a model of HFD-fed female mice showing a metabolic profile resembling the human insulin resistance (Fig. 1c–e)^41^. Analysis of the offspring revealed significant deficits in LTP at CA3–CA1 synapses (Fig. 2d) and hippocampus-dependent learning and memory tasks (Fig. 2a–c). Surprisingly, in the second and the third generations of HFD descendants we found behavioral and electrophysiological alterations similar to those observed in F1_HFD_ mice (Fig. 3a–h). The unbiased analysis of synaptic plasticity gene expression also revealed altered expression of several targets in the hippocampus of F0 HFD mother’s descendants (Supplementary Fig. 3a) including decreased amounts of neuronal activity-related *Bdnf* exons (I, IV, IXa)^42,43^ (Fig. 4a) and BDNF protein (Fig. 4b). The first set of our data demonstrated that maternal diet multigenerationally affects gene expression, hippocampal plasticity, and cognitive functions similarly to adverse environments and psychological stress^44^.

The intergenerational epigenetic transmission may be the consequence of behavioral and/or germline transfer of a phenotype. The first condition occurs when environmental factors persist across the generations as, for example, in case of the transmission of maternal care behavior^45,46^. In our experimental model, the descendants of HFD mothers did not exhibit significant changes of metabolic profile resembling those observed in their ancestor (compare Fig. 2e–h and Supplementary Fig. 2d–g with Fig. 1b–e). However, we found low BDNF plasma levels in HFD progeny (Supplementary Fig. 3e), which might contribute to the multi-organ downregulation of *Bdnf* observed in HFD descendants (Fig. 4c–e and Supplementary Fig. 3d). Moreover, we cannot exclude that other metabolic changes or unmeasured alterations might occur in our experimental model and influence the phenotype of next generations (e.g., cryptic maternal effects, transfer of parental microbiota to offspring, or effects of seminal fluid on maternal behavior or physiology)^47,48^. CF experiments keep out the involvement of maternal behavior in HFD-induced intergenerational cognitive effects (Supplementary Fig. 4a–d), and IVF data point out the male sperm as vehicle of epigenetic phenotype (Supplementary Fig. 4e–h). A common characteristic of intergenerational phenotype transmitted via the germline is to find an epigenetic mark in both somatic and gametic tissues^49^. We found lower levels of gene activation-related molecular marks such as H3K9ac and H3K4me3 in both germline and hippocampus of HFD mother’s male descendants (Fig. 4c, e). Diet-dependent epigenetic modifications may be then inheritable but they should also be editable in response to environmental factors^39^. Indeed, exposure of F1_HFD_ male mice to NEE counteracted the multigenerational transmission of HFD detrimental effects on brain functions, leading to almost complete rescue of learning, memory, and synaptic plasticity in F2_HFD NEE_ and F3_HFD NEE_ mice (Fig. 5a–d and Supplementary Fig. 5b). Of note, we found a rescue of epigenetic activation markers on the *Bdnf* regulatory sequences in the germline of mice exposed to NEE (i.e., F1_HFD_ NEE; Supplementary Fig. 5a). NEE may affect germ cell epigenome of F1_HFD_ mice by multiple mechanisms, including changes in circulating neuroendocrine hormones and neurotrophins (e.g., BDNF), action of non-coding RNAs, or paternally induced alterations in maternal behavior^50^. The discrepancy between our data and those from previous works investigating the intergenerational effects of NEE in SD condition may be due to the type of parental exposure (maternal vs. paternal), the critical phase of exposure (pre or post weaning), and/or the studied brain area^51–53^.

Our findings suggest that the multigenerational opposite effects of HFD and NEE on learning and memory may be mediated by epigenetic changes targeting the same molecular machinery (Supplementary Fig. 5a). However, how nutrient-related signals can trigger the intergenerational transmission of HFD effects remains largely unexplored.

An intriguing hypothesis is that both systemic HFD-dependent insulin resistance and BDNF deficit are involved in the mother to offspring transmission of HFD-dependent cognitive impairment by changing the chromatin remodelers’ recruitment on *Bdnf* regulatory sequences. In F0 HFD mothers, we found lower plasma levels of BDNF (Fig. 6b) and hypophosphorylation of ovarian TrkB receptor (Fig. 6c), which can account for the reduced CREB activation. Moreover, F0 HFD mother’s gonads constitutively showed higher inhibition of IRS1 (Fig. 6c), a molecular marker of insulin resistance, which leads to increased nuclear translocation of FOXO3a. Both CREB and FOXO3a are able to bind HAT/HDAC enzymes and regulate their recruitment on chromatin. Accordingly, FOXO3a was more bound to both HDAC2 and SIRT2 in the ovaries of F0 HFD mothers (Fig. 6d). More importantly, in HFD gonads the binding of both HDAC enzymes was increased, whereas the recruitment of CBP was inhibited on the *Bdnf* gene *loci* showing reduced H3K9 acetylation in the offspring of HFD mothers (Fig. 6e). We hypothesize that BDNF intergenerationally auto-regulates its expression through a positive feedback mechanism aimed to prime next generation to the environmental conditions (i.e., parental imprinting)^54^. Accordingly, BDNF administration to HFD-fed mothers counteracted the intergenerational transmission of both cognitive impairment and *Bdnf* downregulation (Fig. 7e–h). It is worth mentioning that BDNF administration exerted anorectic effects on HFD-fed mice (Fig. 7b) but without significantly interfering with peripheral insulin resistance (Fig. 7c and Supplementary Fig. 6a) nor counteracting IRS1 inhibition in the ovaries (Fig. 7d). However, the critical role of insulin resistance as trigger of HFD-dependent intergenerational effects on hippocampal plasticity was highlighted by the results we obtained in *p66Shc* KO mouse model. F1_HFD_ *p66Shc* mice did not show any significant changes in learning, memory, and BDNF levels compared to SD mice (Fig. 8b–d).

It is still debated whether the mouse embryo can retain histone modifications acquired during oocyte maturation or they are completely erased during embryo development^55–58^. In addition to BDNF and insulin signaling, alteration of other metabolic signaling pathways affecting chromatin structure, such as phosphorylation of histone H3 by the nutrient sensor AMPK, modulation of flavin adenine dinucleotide-dependent histone demethylase LSD1^59^, or HFD-dependent change of microRNAs^60^, might be involved in the intergenerational modifications of epigenetic marks. Finally, epigenetic changes identified on the *Bdnf* promoters might primarily occur in the developing F1 embryos and lead to multi-organ inhibition of *Bdnf* expression.

**Fig. 9:**
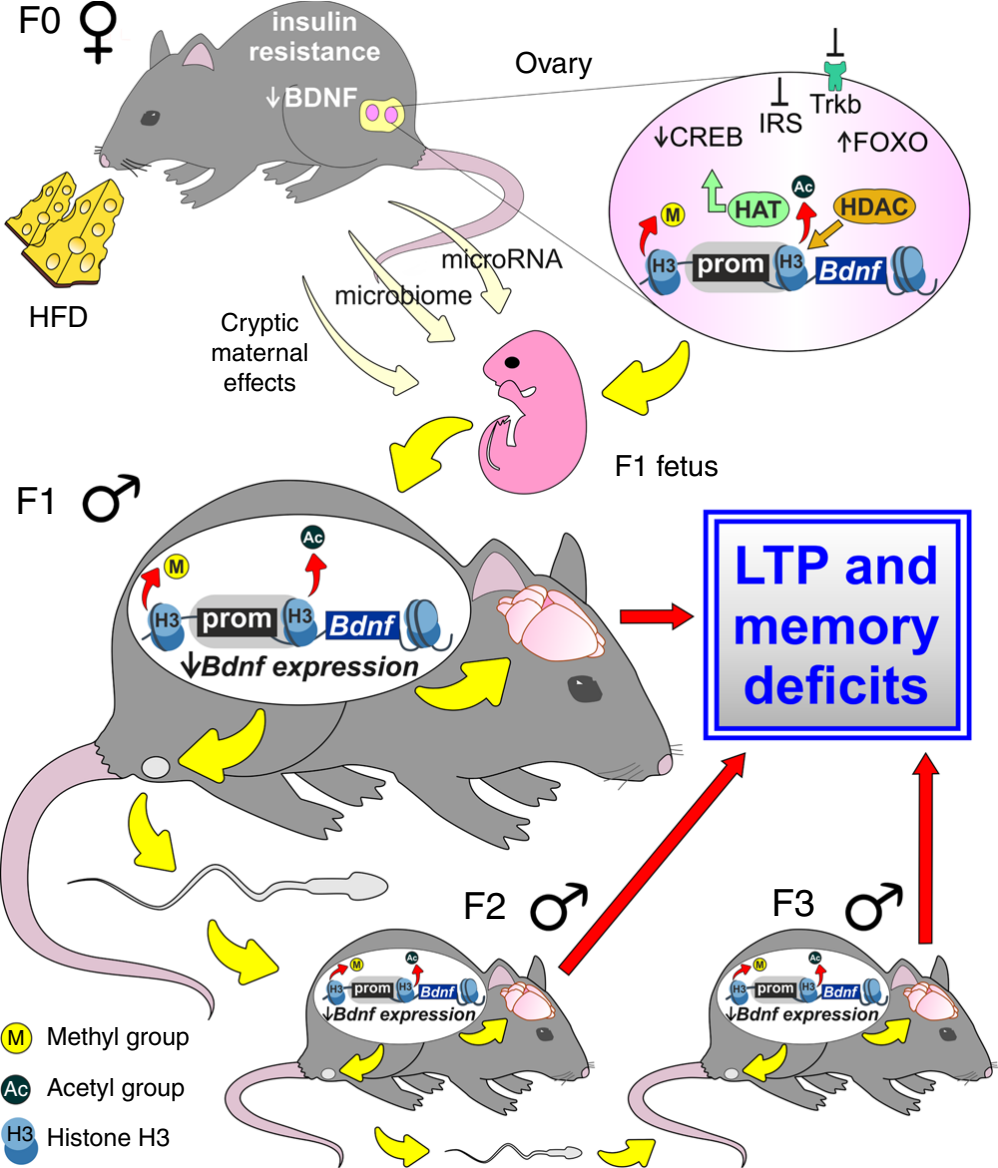
Flow chart of findings. Maternal (F0) HFD causes molecular and behavior changes in the offspring (F1) via gametic mechanisms involving epigenetic inhibition of *Bdnf* expression. Both low BDNF plasma levels and insulin resistance in mothers lead to alterations of BDNF and insulin signaling in the ovaries and change the recruitment of histone acetyl-transferases (HAT) and histone deacetylases (HDAC) on the *Bdnf* promoters. These epigenetic modifications may be transferred from oocytes to embryo. Additional mechanisms including maternal microbiota transfer, microRNA, and cryptic maternal effects may be involved in the HFD-related intergenerational modification of *Bdnf* epigenetic marks. The outcome is a multi-organ inhibition of *Bdnf* expression leading to LTP and memory deficits. The propagation of the same epigenetic changes via male sperm is responsible for the transmission of HFD-dependent brain damage to the next generations (F2 and F3)

Our findings demonstrate a multigenerational effect of maternal high-fat feeding on the cognitive function and reveal epigenetic markers of early-life environmental exposure. More genes critically involved in synaptic plasticity regulation and other epigenetic mechanisms (including DNA methylation or microRNA expression) may play a role in the HFD-dependent multigenerational effects on cognitive function. However, alteration of both BDNF and insulin signaling during the embryo development appear to be primarily responsible for the transmission of brain vulnerability to the next generations (Fig. 9). Importantly, these modifications are transmittable across the generations and represent a sort of molecular switch for the vulnerability to the lifestyle-related diseases^61,62^. Identifying the genes most susceptible to disease-related epigenetic changes and the molecular mechanisms that ensures locus-specific targeting are great challenges for personalized medicine in the near future.

## Methods

### Animals

Female C57BL/6 mice (30 days old), derived from the Animal Facility of Catholic University, were used and randomly assigned to two feeding regimens: (i) SD (control) and (ii) HFD until they were ready for mating and they were weighed weekly. Female mice (F0) were paired for breeding at the end of the fourth week of dietary regimen. Male mice are removed from the female’s cage after 1–2 days of mating and are exposed to HFD only during this time lapse. The same male mouse was paired, at different times, with both a F0 SD female and a F0 HFD female mouse. A subset of F0 female mice was sacrificed prior to mating for blood and tissue collection. F0 pregnant females were usually fed with HFD until the second week of lactation; a subgroup of them switched, instead, to SD after delivery (generating SD and F1_HFD NL_ mice). Male mice were always fed with standard chow. A subset of male C57BL/6 offspring (F1_HFD_) was paired for breeding with control females giving rise to the second generation (F2_HFD_). Similarly, male mice of the second generation were weaned onto standard chow and a subset of them was paired for breeding with control, SD-fed females to produce the third generation (F3_HFD_). Different subsets of male and female pups weaned onto standard chow underwent behavioral testing or were euthanized at 10–12 weeks for electrophysiological analyses or tissue collection. A maximum of two male offspring was taken from each litter for each experimental set to remove any litter effects. The c129/sv F0 female mice harboring the homozygous deletion of p66Shc, derived from the Animal Facility of Catholic University, were fed with SD or HFD and the offspring were generated resembling the experimental model set up for wild-type mice.

### Ethics and animal use statement

All animal procedures were approved by the Ethics Committee of the Catholic University and were fully compliant with Italian (Ministry of Health guidelines, Legislative Decree No. 116/1992) and European Union (Directive No. 86/609/EEC) legislations on animal research. The methods were carried out in strict accordance with the approved guidelines.

### IP glucose tolerance test and HOMA-IR

Animals are fasted for approximately 16 h, and fasted blood glucose levels are determined before a solution of glucose (2 g of glucose kg^−1^ weight) is administered by IP injection (volume of IP glucose injection = 10 μL g^−1^ body weight). Subsequently, the blood glucose level is measured through a glucometer at 15, 30, 60, 90, 120, 150, and 180 min after glucose injection by placing a small drop of blood tail on test strip. HOMA-IR was calculated as fasting plasma insulin (in picomoles L^−1^) × fasting plasma glucose (in millimoles L^−1^)/22.5.

### Diet, housing conditions, and drug administration

Diets were from Mucedola (Italy). Chows were stored at 4 °C and diet in cages was replaced weekly to prevent degradation. Mice were housed in cages 25 cm × 20 cm × 15 cm. For the NEE protocol, larger cages (60 cm × 35 cm × 20 cm) were used containing a small house and a running wheel for voluntary exercise along with a number of toys that were changed three times a week with new ones of different shape and color. A group of F1_SD_ and F1_HFD_ male mice were exposed after weaning to NEE for 4 weeks before mating with control females to obtain F2_SD NEE_ and F2_HFD NEE_ mice. F2_SD NEE_ and F2_HFD NEE_ male mice were then crossed with control females to generate F3_SD NEE_ and F3_HFD NEE_, respectively.

A group of F0 SD-fed and F0 HFD-fed female mice were IP injected with vehicle or 20 ng BDNF 3 times per week for 4 weeks before mating. Different cohorts were sacrificed at the end of treatment for molecular analyses. The animals were housed under a 12-h light–dark cycle at room temperature (RT; 19–22 °C), fed with their respective diets and received water ad libitum. Weight and food consumption were weekly monitored.

### Enzyme-linked immunosorbent assay (ELISA)

Blood samples were collected from the retro-orbital plexus with sterile glass Pasteur pipettes. After centrifugation, plasma was separated and stored at −80 °C until further use. Plasma insulin and plasma/hippocampus BDNF concentrations were determined by using commercially available ELISA kits (Immunological Sciences). The assays were performed according to the manufacturer’s instructions.

### Behavioral experiments

Behavioral tests were carried out from 9 a.m.to 4 p.m. and data were analyzed in blind using an automated video tracking system (Any-Maze™). Recognition memory was evaluated by NOR test. On the first day, animals were familiarized for 10 min to the test arena (45 cm × 45 cm). On the second day (training session), they were allowed to explore two identical objects placed symmetrically in the arena for 10 min. On the third day (test session), a new object replaced one of the old objects. Animals were allowed to explore for 10 min and a preference index, calculated as the ratio between time spent exploring the novel object and time spent exploring both objects, was used to measure recognition memory.

Spatial learning and memory were assessed using the MWM test. A circular plastic pool (127 cm in diameter) filled with water colored with nontoxic white paint to obscure the location of an hidden platform was used as experimental apparatus. The pool was ideally separated into four equal quadrants (NE, corresponding to the target quadrant, SE, NW, and SW) and the platform (10 cm × 10 cm) was placed at the center of the target quadrant. Visual cues were placed on the walls around the pool to orient the mice. Animals were trained for 4 days, six times a day and the probe test was administered 24 h after the last training day. Starting positions were varied daily and latencies to reach the platform were recorded. In the probe test, the platform was removed and time spent in each quadrant was measured (60 s of test duration).

Locomotor activity was assessed using the Open Field test. Briefly, the apparatus consisted of a square arena (45 cm × 45 cm) with no visible extra-maze cues aside for the video recording camera. Animal were introduced in the center of the arena and were allowed to explore for 10 min while being monitored with a video-tracking system. Total distance traveled was measured and reported minute by minute.

### Ex vivo electrophysiology on hippocampal slices

fEPSPs were elicited in the CA1 area of hippocampus by placing a bipolar concentric stimulating electrode (FHC) in the Schaffer collateral pathway. The electrode was connected to a constant current isolated stimulator (Digitimer). A low impedance glass pipette (1–2 MΩ) was filled with artificial cerebrospinal fluid and placed immediately below the CA1 stratum pyramidale. Recordings were performed in current clamp I = 0 mode, using a Multiclamp 700 A/Digidata 1440 A system (Molecular Devices). First, the input–output relationship was constructed and the stimulus intensity resulting in 30% of maximal response amplitude was found. After achieving a stable baseline response, LTP was induced by using the high-frequency stimulation protocol (1 train of stimuli at 100 Hz, lasting 500 ms, repeated four times with an inter-train interval of 20 s). After LTP induction, fEPSP amplitude and slope were monitored for at least 60 min and data were analyzed^63^.

### Tissue and germ cell collection

Epididymides were removed and separated from fat. Radial slits were made in each of the cauda epididymides. Later, epididymides were placed in microcentrifuge tubes containing 1× phosphate-buffered saline (PBS) and shook on an orbital shaker for 10 min to facilitate the swim out of the sperm. The epididymal tissues were allowed to settle for 15 min and the sperm suspension was then separated and pelleted at 16,000 × *g* in a new microcentrifuge tube. The pellet was frozen at −80 °C until RNA extraction. Whole ovaries were collected from the ovarian bursa and transferred in M2 medium (Sigma). Ovaries were mechanically punctured using a 30-gauge syringe needle in medium consisting of 5% heat-inactivated fetal bovine serum in PBS. Isolated follicles were washed with media to eliminate debris. For chromatin immunoprecipitation (ChIP) analyses, mice were anesthetized with a cocktail of ketamine (80 mg kg^−1^, intramuscular (i.m.)) and medetomidine (1 mg kg^−1^, i.m.) and transcardially perfused with an oxygenated Ringer’s solution (pH: 7.3), followed by 4% freshly depolymerized paraformaldehyde in 0.1 M PBS (pH:7.4). The brain, epididymides, and ovaries were isolated and post-fixed overnight at 4 °C and then transferred to a solution of 30% sucrose in PBS for 2 days. Coronal brain sections (45-μm thick) containing hippocampi were then cut with a vibratome (VT1000S) and floated in ice-cold PBS.

Hippocampi were isolated under optic microscope and minced through a 10-mL syringe with decreasing needle size (18–22 gauge). To separate germ cells and follicles, both epididymides and ovaries were isolated and handled as above mentioned.

### Real-time PCR

Quantitative RT-PCR amplifications were performed using SYBR GREEN qPCR Master Mix (Fisher Molecular Biology) on AB7500 instrument (Life Technologies) according to the manufacturer’s instructions. The thermal cycling profile featured a pre-incubation step of 94 °C for 10 min, followed by 40 cycles of denaturation (94 °C, 15 s), annealing (55 °C, 30 s), and elongation (72 °C, 20 s). Melting curves were subsequently generated (94 °C for 15 s, 50 °C for 30 s, slow heating to 94 °C in increments of 0.5 °C).

Melting curve analyses confirmed that only single products had been amplified. The primer sequences are shown in Supplementary Table 2. All data were normalized by reference to the amplification levels of the actin gene; a reference dye was included in the SYBR master mix. The thresholds calculated by the software were used to calculate specific mRNA expression levels using the cycle-at-threshold (Ct) method, and all results are expressed as fold changes (compared to control) for each transcript, employing the 2−ΔΔCt approach.

For PCR array experiments, an RT2 Profiler Custom PCR Array (PAMM-126Z) was used to simultaneously examine the mRNA levels of 89 genes, including 5 housekeeping genes in 96-well plates according to the protocol of the manufacturer (Qiagen). Each reaction included 40 ng of total RNA and the proper negative controls (no reverse transcription, no template). RNA of all samples was analyzed in triplicate, and data were normalized for glyceraldehyde 3-phosphate dehydrogenase levels by the ΔΔCt method. All results are shown in Supplementary Table 1.

### Single-cell droplet digital PCR (ddPCR)

BDNF gene expression in CA1 hippocampal neurons from brain slices was assessed by single-cell ddPCR. Briefly, whole-cell recording pipettes (3–4 MΩ) were presterilized and filled with a Rnase-free intracellular solution containing (in mM): 123 K-gluconate, 12 KCl, 10 HEPES, 0.2 EGTA, 4 Mg2ATP, 0.3 Na3GTP, 10 phosphocreatine, 1 U μL^−1^ recombinant Rnase inhibitor (Ambion), pH 7.25–7.30 (osmolarity 300 mOsm). The intraneuronal contents (∼1–2 μL) were collected into the tip of the patch pipette by applying negative pressure and transferred in RNase/DNase-free tubes. In addition, each experiment included at least one negative control consisting of a recording pipette used without achieving the whole-cell configuration. The negative controls were processed in the same manner as the rest of the samples to determine the amount of contamination during sample collection and amplification. Final volume was brought up to 11–12 μL by adding Single Cell DNase I/Single Cell Lysis solution of Ambion Single Cell-to-CT kit (Life Technologies, Grand Island, NY). Cell lysates were incubated at RT for 5 min and subjected to cDNA synthesis according to the manufacturer’s instruction (Ambion Single Cell-to-CT Kit). Preamplification reaction, performed with BDNF and mAldolase primer mix, according to the manufacturer’s protocol (SsoAdvanced^TM^ PreAmp Supermix), was diluted 1:5 or 1:10 and used for quantification by ddPCR on QX200 Droplet Digital PCR System (Bio-Rad, Hercules, CA, USA) at 59 °C using EvaGreen ddPCR Supermix (Bio-Rad), 200 nM primer concentration, and 2 μL of diluted cDNA^64^. Six single hippocampal neurons per experimental group have been analyzed in duplicate in three different experiments. The primer sequences are shown in Supplementary Table 2.

### Chromatin immunoprecipitation

Hippocampi or germ cells were resuspended in 200 μL lysis buffer containing 1% sodium dodecyl sulfate (SDS), 50 mM Tris-HCl pH 8.0, and 10 mM EDTA and sonicated on ice with six 10-s pulses with a 20-s interpulse interval. Sample debris was removed by centrifugation and supernatants were precleared with protein-G Sepharose 4B beads (Sigma-Aldrich) for 1 h at 4 °C. Two micrograms of specific antibody or control IgG were added overnight at 4 °C. Immune complexes were collected by incubation with protein-G Sepharose 4B beads for 2 h at 4 °C. After seven sequential washes, immune complexes were eluted from beads by vortexing in elution buffer (1% SDS and NaHCO_3_ 0.1 M; pH 8.0). NaCl was added (final concentration 0.33 M), and cross-linking was reversed by incubation overnight at 65 °C. DNA fragments were purified by using the PCR DNA Fragments Purification Kit (Geneaid)^65^. The primer sequences are shown in Supplementary Table 2.

PCR conditions and cycle numbers were determined empirically and each PCR reaction was performed in triplicate. Data are expressed as the percentage of input calculated by the Adjusted input value method according to the manufacturer’s instructions (ThermoFisher Scientific ChIP Analysis). To calculate the Adjusted input, the Ct value of input was subtracted by 6.644 (i.e., log2 of 100). Next, the percentage of input of samples was calculated using the formula: 100 × 2^(Adjusted input − Ct(ChIP). The percentage of input of IgG samples was calculated using the formula 100 × 2^(Adjusted input − Ct(IgG).

### Co-immunoprecipitation

Tissues were lysed in IP buffer (KCl 50 mM, Tris-HCl 50 mM pH 8, EDTA 10 mM, and 1% Nonidet P-40) and part of the lysate was used for total input. The lysates were precleared for 30 min with empty protein G-sepharose 4B beads (Sigma Aldrich) before being incubated with 1–2 μg of specific antibody or IgG (negative control) and fresh protein G matrix. After 6 h of incubation at 4 °C with rotating mixer, protein G bound immune complexes were collected by centrifugation (22,000 × *g*, 1 min) and washed six times with IP buffer. Beads were finally resuspended in 1 × Laemmli buffer and boiled for 5 min. Eluted proteins were resolved by SDS-polyacrylamide gel electrophoresis (PAGE) and immunoblotting.

### Western blotting

Tissues (hippocampi or ovaries) were lysed in ice-cold lysis buffer (NaCl 150 mM, Tris-HCl 50 mM pH 7.4, EDTA 2 mM) containing 1% Triton X-100, 0.1% SDS, 1 × protease inhibitor cocktail (Sigma-Aldrich), 1 mM sodium orthovanadate (Sigma-Aldrich), and 1 mM sodium fluoride (Sigma-Aldrich). Cells were incubated for 10 min on ice with occasional vortexing and spun down at 22,000 × *g*, 4 °C. Supernatant was quantified for protein content (DC Protein Assay; Bio-Rad). Equal amounts of protein were diluted in Laemmli buffer, boiled, and resolved by SDS-PAGE. The primary antibodies (available in Supplementary Table 3) were incubated overnight and revealed with horseradish peroxidase-conjugated secondary antibodies (Cell Signaling Technology Inc., Danvers, MA). Antibody against phospho-TrkB Tyr816 was a kind gift of Moses V. Chao^66^. Expression was evaluated and documented by using UVItec Cambridge Alliance. Images shown were cropped for presentation with no manipulations. All uncropped blots are included in the Source Data file.

### CF and IVF

For CF experiments, breeding pairs made by male SD mice and female F0 SD or F0 HFD mice were simultaneously set up. After the birth of both SD and F1_HFD_ litters, the whole litters were removed and put with two different F0 SD-fed mothers. At weaning, pups were separated based on sex and tested both at a molecular and behavioral level.

IVF was performed in collaboration with the European Mouse Mutant Archive in Monterotondo (Rome, Italy). Briefly, the sperm from SD or F1_HFD_ mice was extracted from cauda epididymides and vasa deferentia of male mice and preserved in cryoprotective medium (18% raffinose 3% skim milk and 477 mM monothioglycerol). The pooled sperm was loaded into French straws and placed onto a polystyrene raft floating on LN2 before being plunged into LN2 and stored in a LN2 tank until used^67^. Cryopreserved sperm was thawed by moving the straw from the cassette in the LN2 tank into a water bath at 37 °C for 30 s. SD females were previously superovulated by an IP injection of 5 IU pregnant mare serum gonadotropin (Intervet, Milan, Italy) followed by 5 IU human chorionic gonadotropin (hCG; Intervet) 48 h later. At 12–14 h post-hCG injection, females were euthanized by cervical dislocation and their oviducts were removed aseptically. After an overnight incubation of ovocytes with sperm of SD or F1_HFD_ mice, two-cell embryos were collected and transferred into recipient psuedopregnant females. All females were always fed with SD.

### Statistical analysis

Sample sizes were chosen with adequate power (0.8) according to results of prior pilot datasets or studies, including our own, which used similar methods or paradigms. Sample estimation and statistical analyses were performed using the SigmaPlot 14.0 software. Data were first tested for equal variance and normality (Shapiro–Wilk test) and the appropriate statistical tests were chosen. The statistical tests used (i.e., Student’s *t* test, Mann–Whitney test, one-way ANOVA, two-way ANOVA) are indicated in the main text and in the corresponding figure legends for each experiment. Post hoc multiple comparisons were performed with Bonferroni correction. All statistical tests were two tailed and the level of significance was set at 0.05. Results are shown as mean ± SEM.

### Reporting summary

Further information on research design is available in the Nature Research Reporting Summary linked to this article.

## Supplementary information

Supplementary Information

Peer Review

Reporting Summary

Source Data

## Data Availability

The datasets generated and/or analyzed during the current study are available from the corresponding author on reasonable request. The source data underlying Figs. 6a, c, d, 7a, d, and 8a and Supplementary Fig. 7a are provided as a Source Data file.
