# Supplementary material for: Maternal insulin resistance multigenerationally impairs synaptic plasticity and memory via gametic mechanisms
Source: Nat Commun. 2019 Oct 22;10:4799. doi: 10.1038/s41467-019-12793-3 (PMC6805915; doi:10.1038/s41467-019-12793-3)
Supplement: Supplementary file 1 — Supplementary Information [file 41467_2019_12793_MOESM1_ESM.pdf]

Supplementary Information

Maternal insulin resistance multigenerationally impairs synaptic plasticity and memory via gametic mechanisms

Fusco et al.

a

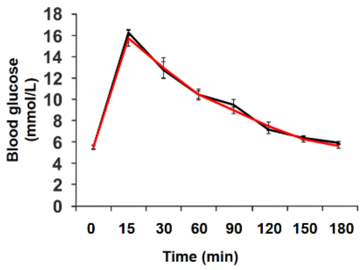

Supplementary Figure 1. HFD offspring do not show changes in insulin sensitivity. (a) Blood glucose levels of SD and $F1_{HFD}$ mice after intraperitoneal glucose tolerance test (IPGTT) (n = 12 mice from 8 litters per group; statistics by unpaired Student's *t*-test). Data are expressed as mean ± SEM.

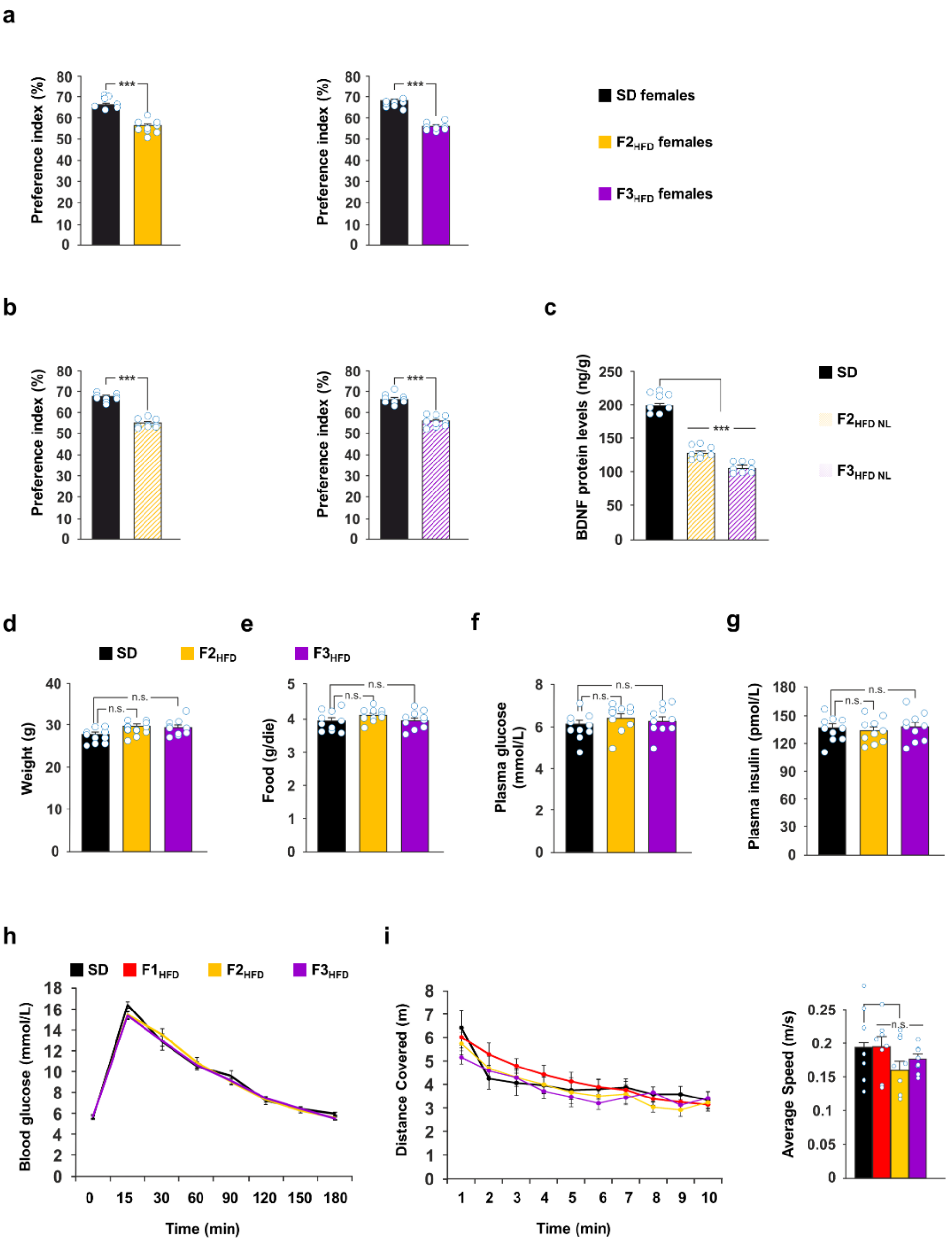

Supplementary Figure 2. Transgenerational HFD effects are not sex-specific. (a) Preference index in NOR paradigm for $F2_{HFD}$ (left) and $F3_{HFD}$ (right) female descendants (n = 9 mice from 6 litters for each group; statistics by unpaired Student's *t*-test). **(b)** Preference index of $F2_{HFD}$ NL (left) and $F3_{HFD}$ NL (right) male mice descended from a progenitor fed with HFD from four weeks before mating until delivery (n = 9 mice from 5 litters for each group; statistics by unpaired Student's *t*-test). **(c)** BDNF levels in the hippocampus of $F2_{HFD}$ NL and $F3_{HFD}$ NL male mice. ELISA assay was performed in duplicate (n = 8 mice from 6 litters per group; statistics by unpaired Student's *t*-test). **(d)** Weight, **(e)** food consumption, **(f)** fasting glucose plasma levels, **(g)** fasting insulin plasma levels and **(h)** IPGTT of SD, $F2_{HFD}$ and $F3_{HFD}$ mice (n = 12 mice from 8 litters per group; statistics by unpaired Student's *t*-test). **(i)** Locomotor activity of SD, $F1_{HFD}$, $F2_{HFD}$ and $F3_{HFD}$ mice quantified by Open Field test (left, n = 10 mice derived from 6 litters for each group; statistics by one-way ANOVA and Bonferroni post hoc) and swimming speed in the 1st day of the MWM (right, n = 8 mice derived from 5-6 litters for each group; statistics by one-way ANOVA and Bonferroni post hoc). Data are expressed as mean ± SEM. *** $p < 0.001$; n.s. not significant.

a

Up-Down Regulation (fold changes)			
GENE	$F1_{HFD}$	$F2_{HFD}$	$F3_{HFD}$
Adam10	1,006	1,4163	-4,0559
**Bdnf**	**-4,7987**	**-2,8565**	**-4,0233**
Camk2g	-3,368	1,4152	1,4113
Crem	1,4228	2,0163	1,4197
Egr2	1,6987	1,6943	2,0167
Ephb2	-2,3807	1,1905	-1,0014
Gnai1	1,0067	-4,7996	1,0039
Grin2d	-2,0112	1,1935	1,1896
Grm3	2,0162	2,0129	1,0061
Junb	2,3935	1,1962	1,6848
Kif17	1,6835	2,3765	1,4144
Ngf	-1,6741	-2,3704	-1,4121
Ngfr	1,6946	2,0117	1,4193
Nr4a1	2,0023	1,9976	1,6847
Ppp1ca	2,0131	1,6829	1,4151

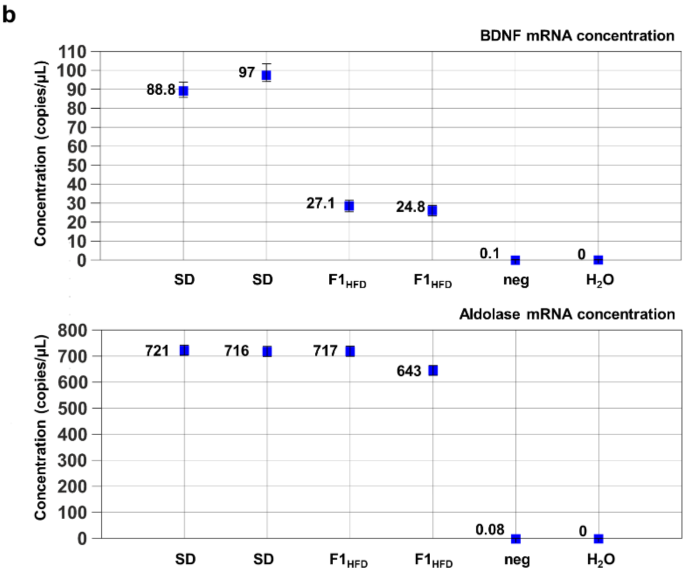

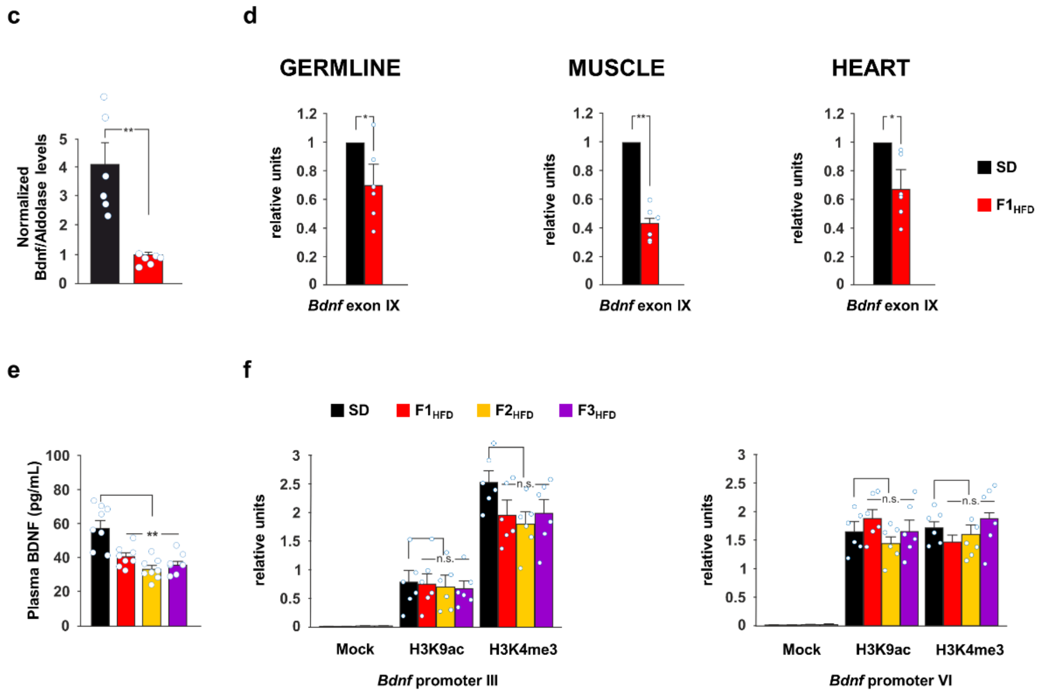

Supplementary Figure 3. Downregulation of BDNF expression in neurons and other cell types. (a) Up-down fold expression changes of synaptic plasticity-related genes more markedly affected in the hippocampus of $F1_{HFD}$, $F2_{HFD}$ and $F3_{HFD}$ mice. **(b)** Representative *Bdnf* transcript concentration (copies µL^-1) in CA1 hippocampal neurons of SD and $F1_{HFD}$ mice. Aldolase was used as control. **(c)** ddPCR data were normalized and plotted as mean ± SEM (n = 6 neurons from 3 mice of different litters per each group). Each sample was analyzed in duplicate (statistics by unpaired Student's *t*-test). **(d)** germline, gastrocnemius muscle and heart Bdnf coding exon IX expression in SD and $F1_{HFD}$ mice (n = 6 mice derived from 4 litters per each group). Gene expression was normalized to actin; experiments were performed in triplicate (statistics by unpaired Student's t-test). **(e)** BDNF plasma levels of SD and HFD descendant male mice measured by ELISA performed in duplicate (n = 8 mice derived from 4 litters per group; statistics by one-way ANOVA and Bonferroni post hoc) **(f)** ChIP assays of H3K9ac and H3K4me3 on the promoters III and VI of *Bdnf* gene in the hippocampus of SD and HFD descendant male mice. Data represent mean values obtained from 6 mice derived from 5 litters for each group; qPCR experiments were performed in triplicate (statistics by one-way ANOVA and Bonferroni post hoc). Data are expressed as mean ± SEM. * $p < 0.05$; ** $p < 0.01$; n.s. not significant.

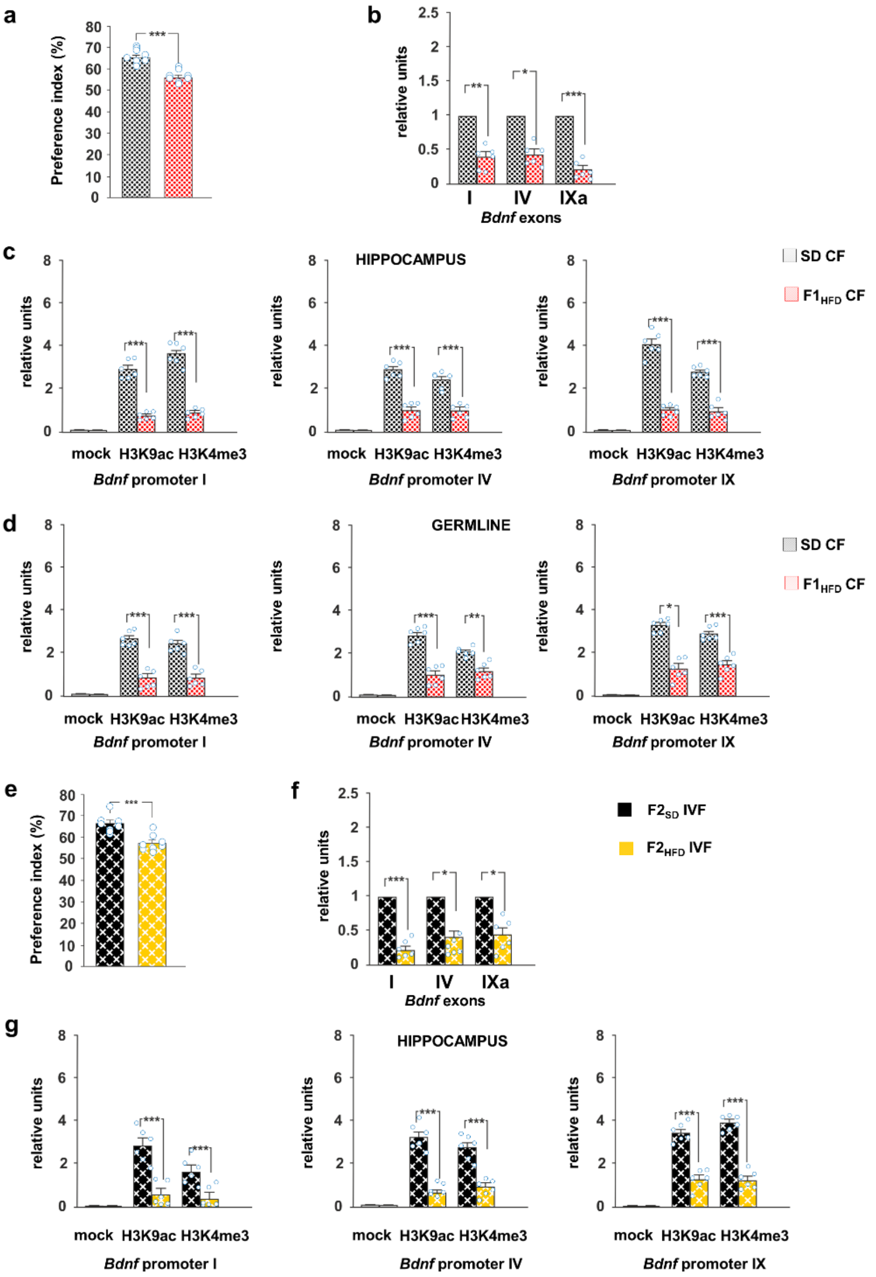

Supplementary Figure 4. HFD intergenerational effects are not mediated by maternal behavior. (a) Preference index of SD and $F1_{HFD}$ mice fostered by F0 SD-fed females (SD CF and $F1_{HFD}$ CF, respectively; n = 9 mice from 6 litters for each group; statistics by unpaired Student's *t*-test). **(b)** Expression of Bdnf exon I, IV and IXa in the hippocampus of SD CF and $F1_{HFD}$ CF mice. Data represent mean values obtained from 6 mice derived from 5 litters for each group; experiments were performed in triplicate (statistics by unpaired Student's *t*-test). **(c)** ChIP assays of H3K9ac and H3K4me3 on the promoters I, IV and IX of *Bdnf* gene in the hippocampus and **(d)** germline of SD CF and $F1_{HFD}$ CF mice. Data represent mean values obtained from 6 mice derived from 4 litters for each group; qPCR experiments were performed in triplicate (statistics by two-way unpaired Student's *t*-test). **(e)** Preference index of mice born through in vitro fertilization (IVF) of oocytes with sperm of SD or $F1_{HFD}$ mice (SD IVF and $F1_{HFD}$ IVF, respectively; n = 8 mice from 4 litters for each group; statistics by unpaired Student's *t*-test). **(f)** Expression of Bdnf exon I, IV and IXa in the hippocampus of SD IVF and $F1_{HFD}$ IVF animals. Data represent mean values obtained from 6 mice derived from 5 litters for each group; experiments were performed in triplicate (statistics by unpaired Student's *t*-test). **(g)** ChIP assays of H3K9ac and H3K4me3 on the promoters I, IV and IX of *Bdnf* gene in the hippocampus of SD IVF and $F1_{HFD}$ IVF mice. Data represent mean values obtained from 6 mice derived from 4 litters for each group; qPCR experiments were performed in triplicate (statistics by unpaired Student's *t*-test). Data are expressed as mean ± SEM. * $p < 0.05$; ** $p < 0.01$; *** $p < 0.001$.

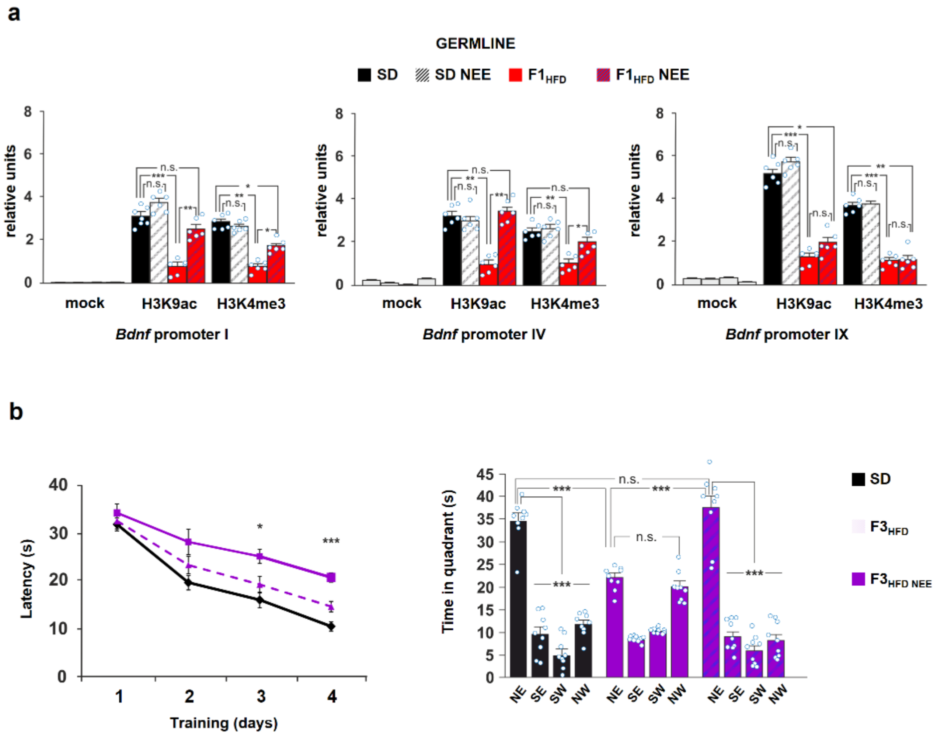

Supplementary Figure 5. NEE induces transgenerational effects on learning and memory. (a) ChIP assays of H3K9ac and H3K4me3 on the promoters I, IV and IX of *Bdnf* gene in the germline of SD, SD NEE, $F1_{HFD}$ and $F1_{HFD}$ NEE male mice. Data represent mean values obtained from 6 mice derived from 4 litters for each group; qPCR experiments were performed in triplicate (statistics by two-way ANOVA and Bonferroni post hoc). **(b)** Latency to reach the hidden platform (left) and time spent in the four quadrants during the probe test (right) in the MWM for SD, $F3_{HFD}$ and $F3_{HFD}$ NEE male mice (n = 9 mice from 6 litters for each group; statistics by two-way ANOVA and Bonferroni post hoc). For latency, significance is indicated between $F3_{HFD}$ and $F3_{HFD}$ NEE mice. Data are expressed as mean ± SEM. * $p < 0.05$; ** $p < 0.01$; *** $p < 0.001$; n.s. not significant.

a

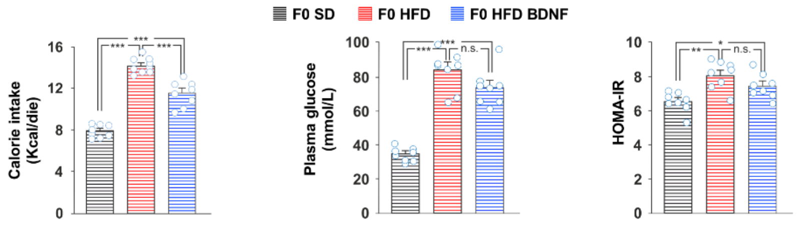

Supplementary Figure 6. BDNF administration does not revert the maternal insulin resistance. (a) Calorie intake (Kcal/die), plasma glucose levels and HOMA index of F0 SD, F0 HFD, and F0 HFD BDNF female mice (n = 8 mice per group; statistics by two-way ANOVA and Bonferroni post hoc). Data are expressed as mean ± SEM. * $p < 0.05$; ** $p < 0.01$; *** $p < 0.001$; n.s. not significant

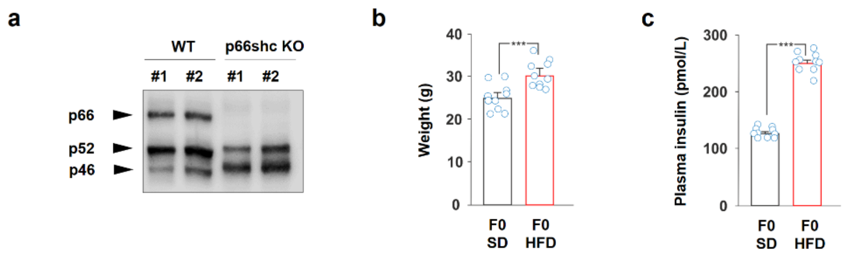

Supplementary Figure 7. Characterization of p66Shc KO model. (a) Immunoblot of p66Sch expression in ovaries of wild-type and *p66Shc* KO female mice. **(b)** Weight and **(c)** fasting insulin plasma levels of SD-fed (black bar) and HFD-fed (red bar) *p66Shc* KO mothers after four weeks of dietary regimen (n = 10 mice per each group; statistics by unpaired Student's *t*-test). Data are expressed as mean ± SEM. Source data are provided as a Source Data file. *** $p < 0.001$

Supplementary Table 1. GENE REGULATION

F1, F2, F3 fold changes of genes analyzed in the PCR array (PAMM-126Z).

Refseq	Symbol	Description	F1 fold change	F2 fold change	F3 fold change
NM_007399	Adam10	A disintegrin and metallopeptidase domain 10	1,006	1,4163	-4,0559
NM_009622	Adcy1	Adenylate cyclase 1	1,6868	1,6814	1,6825
NM_009623	Adcy8	Adenylate cyclase 8	1,003	1,0036	1,1825
NM_009652	Akt1	Thymoma viral proto-oncogene 1	1,6804	1,4168	1,4115
NM_018790	Arc	Activity regulated cytoskeletal-associated protein	1,4193	1,4177	1,4168
NM_007540	Bdnf	Brain derived neurotrophic factor	-4,7987	-2,8565	-4,0233
NM_177407	Camk2a	Calcium/calmodulin-dependent protein kinase II alpha	-1,4094	-1,1903	-1,1864
NM_178597	Camk2g	Calcium/calmodulin-dependent protein kinase II gamma	-3,368	1,4152	1,4113
NM_007664	Cdh2	Cadherin 2	1,6969	1,1964	1,1913
NM_009883	Cebpb	CCAAT/enhancer binding protein (C/EBP), beta	1,0037	1,0031	1,1918
NM_007679	Cebpd	CCAAT/enhancer binding protein (C/EBP), delta	1,0008	-1,9898	1,0023
NM_007726	Cnr1	Cannabinoid receptor 1 (brain)	1,1986	1,004	-1,1889
NM_133828	Creb1	CAMP responsive element binding protein 1	1,001	-1,001	-1,001
NM_013498	Crem	CAMP responsive element modulator	1,4228	2,0163	1,4197
NM_007864	Dlg4	Discs, large homolog 4 (Drosophila)	1,0068	1,1908	1,0055
NM_007913	Egr1	Early growth response 1	1,6867	1,6865	1,6867
NM_010118	Egr2	Early growth response 2	1,6987	1,6943	2,0167
NM_018781	Egr3	Early growth response 3	1,1934	1,0074	-1,4031
NM_020596	Egr4	Early growth response 4	-1,0005	1,6814	1,1867
NM_010142	Ephb2	Eph receptor B2	-2,3807	1,1905	-1,0014
NM_010234	Fos	FBJ osteosarcoma oncogene	1,4119	1,9954	1,6853
NM_176942	Gabra5	Gamma-aminobutyric acid (GABA) A receptor, subunit alpha 5	-1,4032	-1,4028	1,0058
NM_010305	Gnai1	Guanine nucleotide binding protein (G protein), alpha inhibiting 1	1,0067	-4,7996	1,0039
NM_008165	Gria1	Glutamate receptor, ionotropic, AMPA1 (alpha 1)	-1,4062	-1,4146	-1,1881
NM_013540	Gria2	Glutamate receptor, ionotropic, AMPA2 (alpha 2)	1,0029	1,0083	1,0068
NM_016886	Gria3	Glutamate receptor, ionotropic, AMPA3 (alpha 3)	1,0062	1,1903	-1,0015
NM_019691	Gria4	Glutamate receptor, ionotropic, AMPA4 (alpha 4)	1,1923	1,1917	1,191
NM_008169	Grin1	Glutamate receptor, ionotropic, NMDA1 (zeta 1)	-1,0033	1,1926	1,001
NM_008170	Grin2a	Glutamate receptor, ionotropic, NMDA2A (epsilon 1)	1,2	1,0066	1,0019
NM_008171	Grin2b	Glutamate receptor, ionotropic, NMDA2B (epsilon 2)	-1,0008	-1,0003	-1,0004
NM_010350	Grin2c	Glutamate receptor, ionotropic, NMDA2C (epsilon 3)	1,4193	1,4166	1,0008
NM_008172	Grin2d	Glutamate receptor, ionotropic, NMDA2D (epsilon 4)	-2,0112	1,1935	1,1896
NM_133442	Grip1	Glutamate receptor interacting protein 1	1,6782	1,1913	1,0028

NM_016976	Grm1	Glutamate receptor, metabotropic 1	1,0073	1,1941	1,0018
NM_001160353	Grm2	Glutamate receptor, metabotropic 2	-1,4047	-1,0013	1,0016
NM_181850	Grm3	Glutamate receptor, metabotropic 3	2,0162	2,0129	1,0061
NM_001013385	Grm4	Glutamate receptor, metabotropic 4	1,7014	1,4298	1,1954
NM_001081414	Grm5	Glutamate receptor, metabotropic 5	1,716	1,1935	1,1985
NM_177328	Grm7	Glutamate receptor, metabotropic 7	1,1969	1,1922	1,4224
NM_008174	Grm8	Glutamate receptor, metabotropic 8	1,6834	1,1962	1,1902
NM_152134	Homer1	Homer homolog 1 (Drosophila)	1,6902	1,4205	1,1914
NM_010512	Igf1	Insulin-like growth factor 1	1,1887	-1,0053	-1,1871
NM_008380	Inhba	Inhibin beta-A	1,1937	-1,1875	-1,4142
NM_010591	Jun	Jun oncogene	-1,186	1,0007	1,1837
NM_008416	Junb	Jun-B oncogene	2,3935	1,1962	1,6848
NM_010623	Kif17	Kinesin family member 17	1,6835	2,3765	1,4144
NM_013692	Klf10	Kruppel-like factor 10	1,6921	1,6875	-1,183
NM_011949	Mapk1	Mitogen-activated protein kinase 1	1,4207	1,689	1,1933
NM_013599	Mmp9	Matrix metallopeptidase 9	1,0051	1,4177	1,6825
NM_010875	Ncam1	Neural cell adhesion molecule 1	1,423	1,4223	1,1909
NM_008689	Nfkb1	Nuclear factor of kappa light polypeptide gene enhancer in B-cells 1, p105	1,1966	-1,1924	1,0042
NM_010908	Nfkbib	Nuclear factor of kappa light polypeptide gene enhancer in B-cells inhibitor, beta	1,223	1,6874	1,685
NM_013609	Ngf	Nerve growth factor	-1,6741	-2,3704	-1,4121
NM_033217	Ngfr	Nerve growth factor receptor (TNFR superfamily, member 16)	1,6946	2,0117	1,4193
NM_008712	Nos1	Nitric oxide synthase 1, neuronal	1,4143	1,6863	1,6719
NM_016789	Nptx2	Neuronal pentraxin 2	-1,4064	-1,3956	-1,3996
NM_010444	Nr4a1	Nuclear receptor subfamily 4, group A, member 1	2,0023	1,9976	1,6847
NM_008742	Ntf3	Neurotrophin 3	1,0049	-1,4109	1,0034
NM_198190	Ntf5	Neurotrophin 5	1,1714	1,3441	1,3863
NM_008745	Ntrk2	Neurotrophic tyrosine kinase, receptor, type 2	1,4286	1,6832	1,0027
NM_021543	Pcdh8	Protocadherin 8	-1,674	-1,6741	-1,4016
NM_008837	Pick1	Protein interacting with C kinase 1	1,4145	1,1869	1,0032
NM_008842	Pim1	Proviral integration site 1	1,1945	1,1902	1,69
NM_008872	Plat	Plasminogen activator, tissue	1,0031	-1,1904	1,0022
NM_021280	Plcg1	Phospholipase C, gamma 1	1,4123	1,4082	1,6621
NM_031868	Ppp1ca	Protein phosphatase 1, catalytic subunit, alpha isoform	2,0131	1,6829	1,4151
NM_013636	Ppp1cc	Protein phosphatase 1, catalytic subunit, gamma isoform	1,4292	1,0101	1,0095
NM_026731	Ppp1r14a	Protein phosphatase 1, regulatory (inhibitor) subunit 14A	1,4226	1,0003	-1,0024

NM_019411	Ppp2ca	Protein phosphatase 2 (formerly 2A), catalytic subunit, alpha isoform	1,1876	1,0009	-1,0014
NM_008913	Ppp3ca	Protein phosphatase 3, catalytic subunit, alpha isoform	1,1934	-1,1864	1,0007
NM_011101	Prkca	Protein kinase C, alpha	-1,1834	-1,1842	-1,1892
NM_011102	Prkcg	Protein kinase C, gamma	-1,1852	1,0021	-1,1913
NM_011160	Prkg1	Protein kinase, cGMP-dependent, type I	1,0024	1,19	1,4128
NM_009001	Rab3a	RAB3A, member RAS oncogene family	-1,1779	1,0025	1,0025
NM_009045	Rela	V-rel reticuloendotheliosis viral oncogene homolog A (avian)	1,1951	1,0049	1,4172
NM_011261	Reln	Reelin	1,4291	1,1961	1,1974
NM_009061	Rgs2	Regulator of G-protein signaling 2	1,9974	1,6785	1,0021
NM_053075	Rheb	Ras homolog enriched in brain	1,1974	-1,1839	1,0046
NM_019812	Sirt1	Sirtuin 1 (silent mating type information regulation 2, homolog) 1	1,0064	-1,1858	-1,1855
NM_020493	Srf	Serum response factor	1,0011	1,1931	1,0017
NM_177340	Synpo	Synaptopodin	1,0088	1,0024	1,0046
NM_011593	Timp1	Tissue inhibitor of metalloproteinase 1	1,2095	1,6688	1,6862
NM_013693	Tnf	Tumor necrosis factor	1,1946	1,6874	1,685
NM_011739	Ywhaq	Tyrosine 3-monooxygenase/tryptophan 5-monooxygenase activation protein	1,0003	1,0022	-1,1893

Blue: statistically significant downregulated genes
Red: statistically significant upregulated genes

Supplementary Table 2. PRIMERS

Primer sequences used for mRNA analyses.

Gene	Primer sequence	
Bdnf exon I	FW	5'-TGAGAGTTGAAGCTTTGCGG-3'
	RV	5'-ATTGTGGCTTTGCTGTCCTG-3'
Bdnf exon II	FW	5'-TGGAAGAAACCGTCTAGAGC-3'
	RV	5'- CTCGGTGTGAGCCGAACC -3'
Bdnf exon III	FW	5'-TATCATCCCTCCCCGAGAG-3'
	RV	5'-CTCTTCGATCTAGAAAGGACC -3'
Bdnf exon IV	FW	5'-AGCATGAAATCTCCCAGCCT-3'
	RV	5'-CGGTCCCCAAGGTTCTAGAC-3'
Bdnf exon V	FW	5'-GTGTAGTTTCATTGTGTGTTCG-3'
	RV	5'-GCAATGTGCTCTAGAGTAGG-3'
Bdnf exon VI	FW	5'-TGGCTGTCGCACGGTTCC-3'
	RV	5'-GTTGTCACGCTTCTGGTCC-3'
Bdnf exon VII	FW	5'-AAAGGGTCTGCGGAACTCC-3'
	RV	5'-CTCTTCCTGTTTCAGCATCC-3'
Bdnf exon VIII	FW	5'-GTCGCTGCGCCTCAGTGG-3'
	RV	5'-CACCAAGCTTTAAAAACTGAGG-3'
Bdnf exon IXa	FW	5'-CAAAGCTGCTAAAGCGGGAGG-3'
	RV	5'- GATCCACTCCAGCTGCAGG -3'
Bdnf exon IX	FW	5'-TGGCTGACACTTTTGAGC-3'
	RV	5'-GTTTGCGGCATCCAGGT-3'
Gapdh	FW	5'-TCCATGACAACTTTGGCATT-3'
	RV	5'-GTTGCTGTTGAAGTCGCAGG-3'
BDNF exon IX (ddPCR)	FW	5'-GGCAGGTTCGAGAGGTCTGA -3'
	RV	5'-TCGATGACGTGCTCAAAAGTG-3'
Aldolase (ddPCR)	FW	5'-CGCTGCCAGTATGTTACTGAGAA-3'
	RV	5'-TGGTCGCTCAGAGCCTTGT-3'

Primer sequences used for ChIP analyses.

Gene		Primer sequence
BDNF promoter I	FW	5’-TCGATTCACGCAGTTGTTCC-3’
	RV	5’-GCACCAGCCGGCTACTGC-3’
BDNF promoter IV	FW	5’-CATGCAATGCCCTGGAACG-3’
	RV	5’-GAGAGCAGTCCTCTCCTCG-3’
BDNF promoter IX	FW	5’-GAAGCTGAGCTCTCTGTGG-3’
	RV	5’-GGTCTCATGAAGAAACTTAAGG-3’

Abbreviations: FW, forward; RV, reverse

Supplementary Table 3. ANTIBODIES

Primary Antibody	Host	Catalogue reference
α-TrkB (80E3)	Rabbit	Cell Signaling #4603
α-pTrkB Tyr^{816}	Rabbit	See Methods
α-FoxO3a	Rabbit	Cell Signaling #2497
α-phospho FoxO3a Ser^{253}	Rabbit	Cell Signaling #9466
α-Irs-1	Rabbit	Cell Signaling #2382
α-phospho Irs-1 Ser^{612}	Rabbit	Cell Signaling #2386
α-beta Actin	Rabbit	Abcam ab8227
α-Sirt1	Rabbit	Millipore #07-131
α-Sirt2 (D4S6J)	Rabbit	Cell Signaling #12672
α-phospho Creb Ser^{133}	Rabbit	Cell Signaling #9198
α-Creb	Mouse	ThermoFisher #MA1-083
α-Hdac2	Mouse	Abcam ab12169
α-Cbp	Rabbit	Abcam ab2832
α-Tri-Methyl-Histone H3 Lys^{4}	Rabbit	Abcam ab8580
α-Histone H3 (acetyl K9)	Rabbit	Abcam ab4441
α-p66Shc	Mouse	BD Bioscience 610878
