# Supplementary material for: Maternal insulin resistance multigenerationally impairs synaptic plasticity and memory via gametic mechanisms
Source: Nat Commun. 2019 Oct 22;10:4799. doi: 10.1038/s41467-019-12793-3 (PMC6805915; doi:10.1038/s41467-019-12793-3)
Supplement: Supplementary file 3 — Reporting Summary [file 41467_2019_12793_MOESM3_ESM.pdf]

nature research

Corresponding author(s): Claudio Grassi and Salvatore Fusco

Last updated by author(s): Sep 17, 2019

Reporting Summary

Nature Research wishes to improve the reproducibility of the work that we publish. This form provides structure for consistency and transparency in reporting. For further information on Nature Research policies, see Authors & Referees and the Editorial Policy Checklist .

Statistics

For all statistical analyses, confirm that the following items are present in the figure legend, table legend, main text, or Methods section.

n/a	Confirmed	
☐	☒	The exact sample size (*n*) for each experimental group/condition, given as a discrete number and unit of measurement
☐	☒	A statement on whether measurements were taken from distinct samples or whether the same sample was measured repeatedly
☐	☒	The statistical test(s) used AND whether they are one- or two-sided<br>*Only common tests should be described solely by name; describe more complex techniques in the Methods section.*
☐	☒	A description of all covariates tested
☐	☒	A description of any assumptions or corrections, such as tests of normality and adjustment for multiple comparisons
☐	☒	A full description of the statistical parameters including central tendency (e.g. means) or other basic estimates (e.g. regression coefficient) AND variation (e.g. standard deviation) or associated estimates of uncertainty (e.g. confidence intervals)
☐	☒	For null hypothesis testing, the test statistic (e.g. *F*, *t*, *r*) with confidence intervals, effect sizes, degrees of freedom and *P* value noted<br>*Give P values as exact values whenever suitable.*
☒	☐	For Bayesian analysis, information on the choice of priors and Markov chain Monte Carlo settings
☒	☐	For hierarchical and complex designs, identification of the appropriate level for tests and full reporting of outcomes
☒	☐	Estimates of effect sizes (e.g. Cohen's *d*, Pearson's *r*), indicating how they were calculated

Our web collection on statistics for biologists contains articles on many of the points above.

Software and code

Policy information about availability of computer code

Data collection	No software was used
Data analysis	No software was used

For manuscripts utilizing custom algorithms or software that are central to the research but not yet described in published literature, software must be made available to editors/reviewers. We strongly encourage code deposition in a community repository (e.g. GitHub). See the Nature Research guidelines for submitting code & software for further information.

Data

Policy information about availability of data

All manuscripts must include a data availability statement. This statement should provide the following information, where applicable:

- Accession codes, unique identifiers, or web links for publicly available datasets
- A list of figures that have associated raw data
- A description of any restrictions on data availability

Provide your data availability statement here.

Field-specific reporting

Please select the one below that is the best fit for your research. If you are not sure, read the appropriate sections before making your selection.

☒ Life sciences ☐ Behavioural & social sciences ☐ Ecological, evolutionary & environmental sciences

For a reference copy of the document with all sections, see nature.com/documents/nr-reporting-summary-flat.pdf

Life sciences study design

All studies must disclose on these points even when the disclosure is negative.

Sample size	Sample sizes were chosen with adequate power (0.8) according to results of prior pilot data sets or studies, including our own, that used similar methods or paradigms. Sample estimation and statistical analysis were performed using SigmaPlot 14 software. Data were first tested for equal variance and normality (Shapiro-Wilk test) and the appropriate statistical tests were chosen as indicated in the section "Statistical analysis" of Methods.
Data exclusions	No animals or data have been excluded from the analysis
Replication	All experiments have been replicated at least three times
Randomization	Female mice from the same litter were randomly assigned to different experimental sets and used as progenitors. A maximum of two male offspring was taken from each litter for each experimental set to remove any litter effects. All information are included in the section "Animals" of Methods.
Blinding	All behavioral tests were performed and analyzed in blind using an automated video tracking system as indicated in the section "Behavioral experiments" of Methods

Reporting for specific materials, systems and methods

We require information from authors about some types of materials, experimental systems and methods used in many studies. Here, indicate whether each material, system or method listed is relevant to your study. If you are not sure if a list item applies to your research, read the appropriate section before selecting a response.

Materials & experimental systems

n/a	Involved in the study	
☐	☒	Antibodies
☒	☐	Eukaryotic cell lines
☒	☐	Palaeontology
☐	☒	Animals and other organisms
☒	☐	Human research participants
☒	☐	Clinical data

Methods

n/a	Involved in the study	
☒	☐	ChIP-seq
☒	☐	Flow cytometry
☒	☐	MRI-based neuroimaging

Antibodies

Antibodies used	A table with all antibodies and catalog numbers has been provided (Supplementary Table 3).
Validation	Each antibody has been validated by the manufacturers. Data are available on the company website or available from the corresponding author on reasonable request.

Animals and other organisms

Policy information about studies involving animals; ARRIVE guidelines recommended for reporting animal research

Laboratory animals	Male and female C57/Bl6 mice (30-60 days old); c129/sv female and male mice harboring the homozygous deletion of p66Shcp (30-60 days old)
Wild animals	N/A
Field-collected samples	N/A
Ethics oversight	All animal procedures were approved by the Ethics Committee of the Università Cattolica Medical School and and were fully compliant with Italian (Ministry of Health guidelines, Legislative Decree No. 116/1992) and European Union (Directive No. 86/609/EEC) legislations on animal research, as indicated in Methods ("Ethics and Animal use Statement" section).

Note that full information on the approval of the study protocol must also be provided in the manuscript.
