# Supplementary figures and images for: Maternal insulin resistance multigenerationally impairs synaptic plasticity and memory via gametic mechanisms
Source: Nat Commun. 2019 Oct 22;10:4799. doi: 10.1038/s41467-019-12793-3 (PMC6805915; doi:10.1038/s41467-019-12793-3)

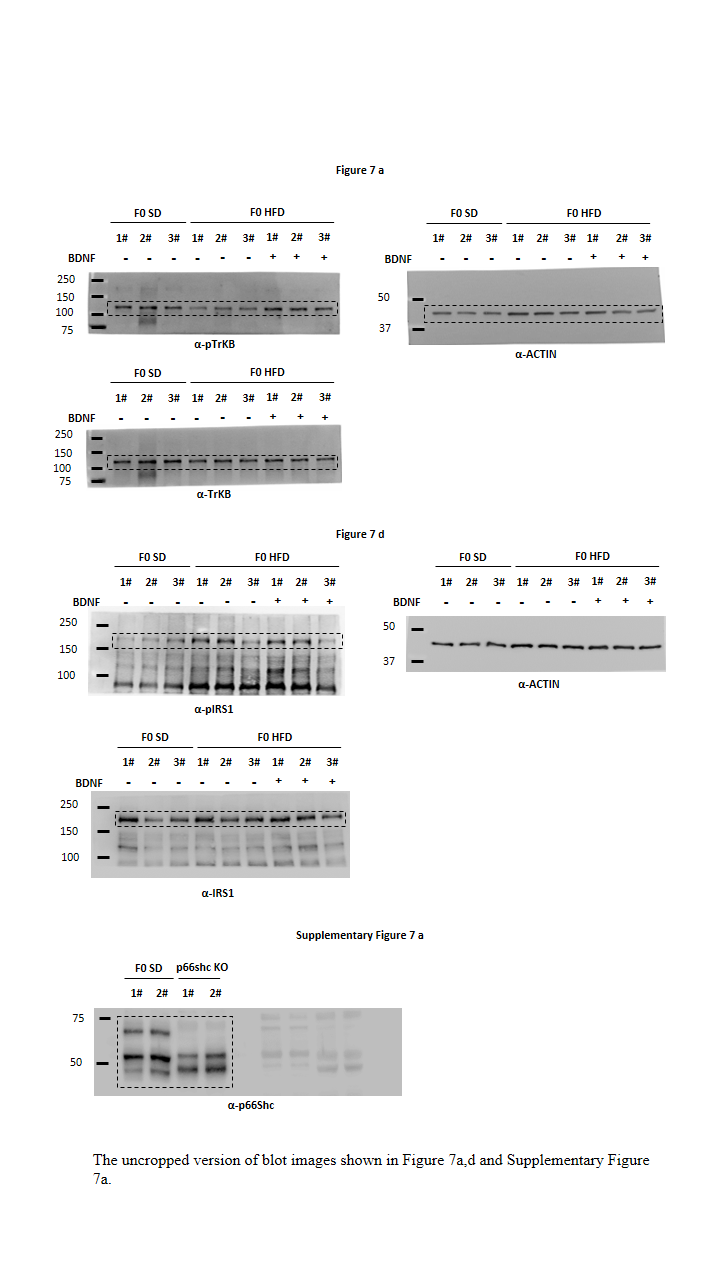

Supplement: Supplementary file 4 — Source Data [file 41467_2019_12793_MOESM4_ESM.zip › 172509_3_supp_4085000_pxzd0h (1).tif]

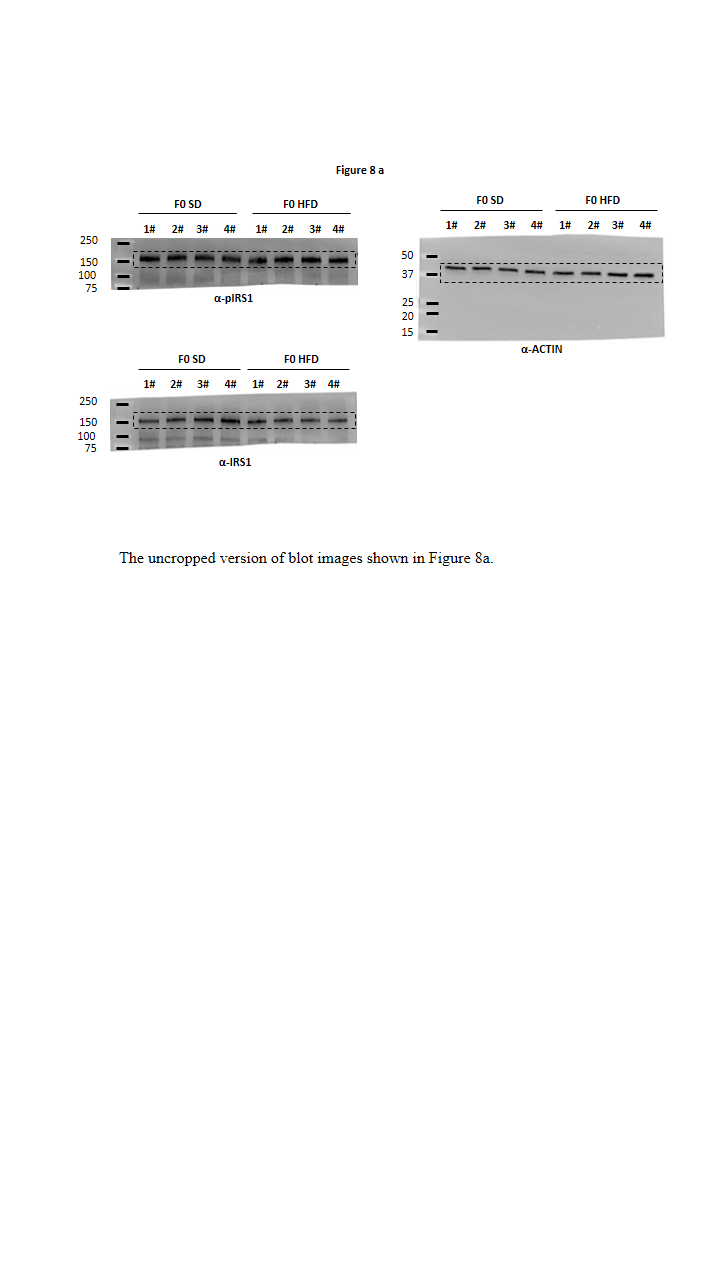

Supplement: Supplementary file 4 — Source Data [file 41467_2019_12793_MOESM4_ESM.zip › 172509_3_supp_4085001_pxz9z7 (1).tif]

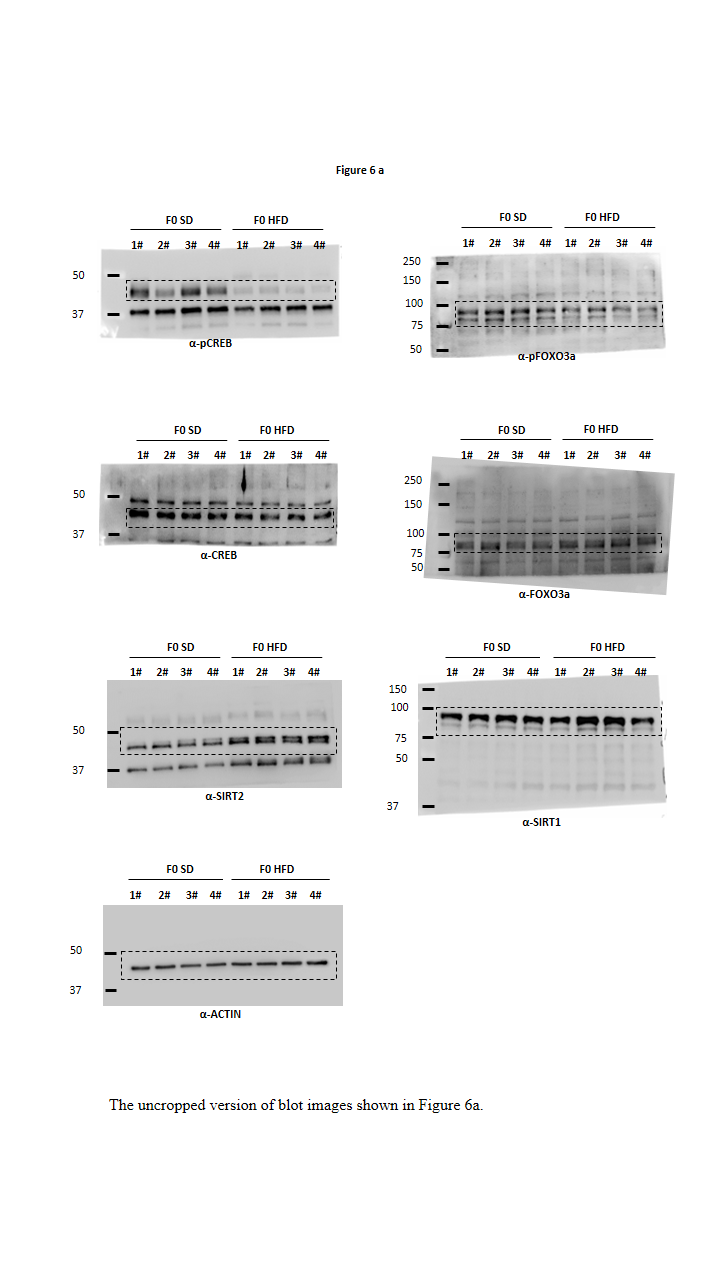

Supplement: Supplementary file 4 — Source Data [file 41467_2019_12793_MOESM4_ESM.zip › 172509_3_supp_4085002_pxz9z7 (1).tif]

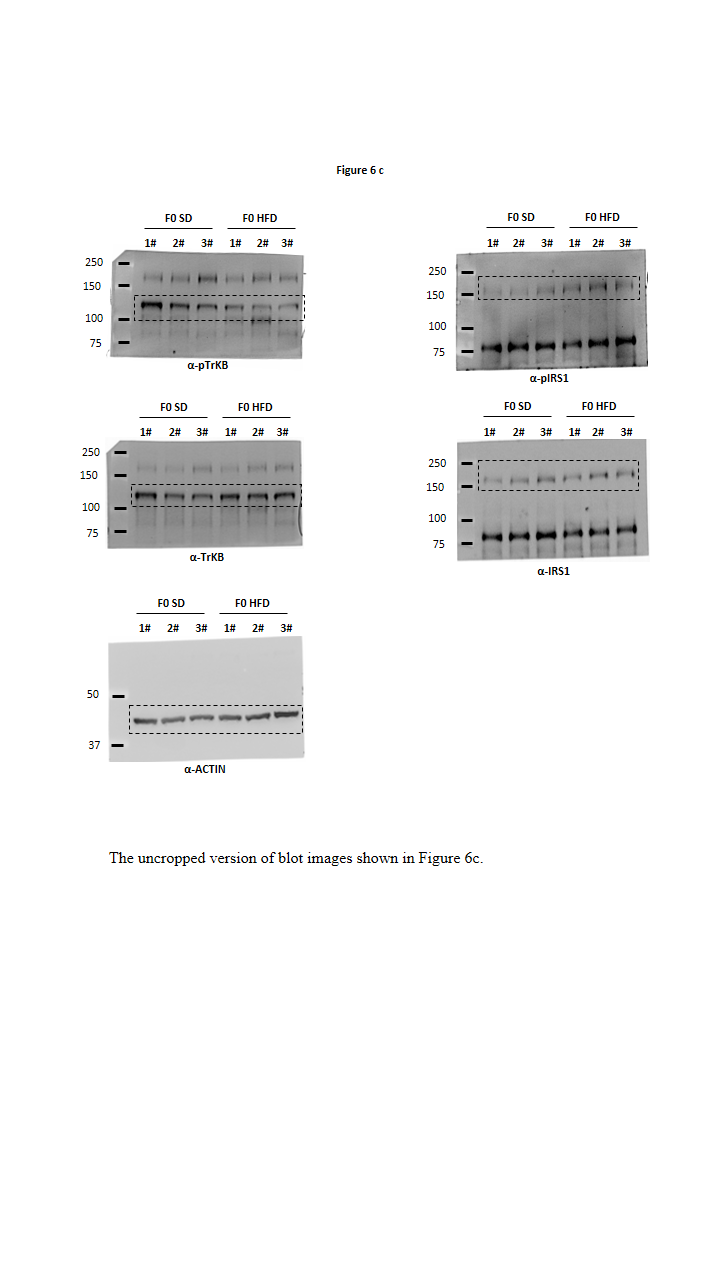

Supplement: Supplementary file 4 — Source Data [file 41467_2019_12793_MOESM4_ESM.zip › 172509_3_supp_4085003_pxz9z7 (1).tif]

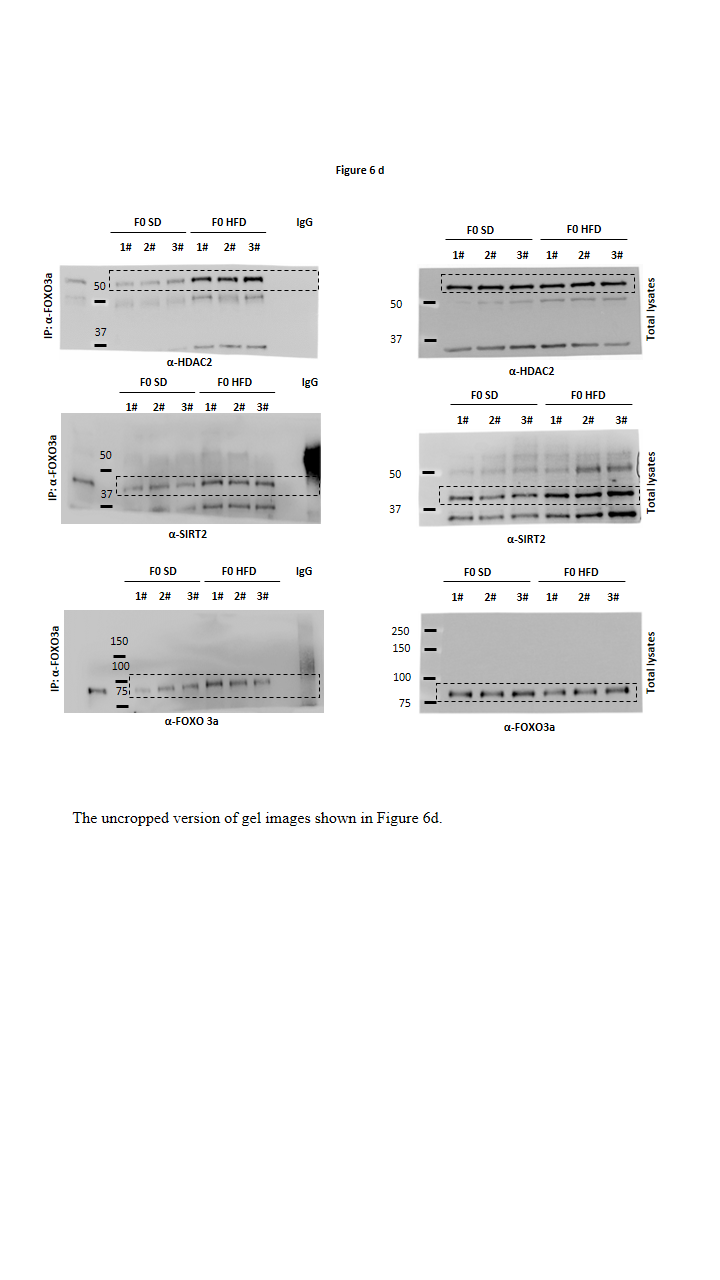

Supplement: Supplementary file 4 — Source Data [file 41467_2019_12793_MOESM4_ESM.zip › 172509_3_supp_4085004_pxz9z7 (1).tif]
