# Supplementary material for: Maternal insulin resistance multigenerationally impairs synaptic plasticity and memory via gametic mechanisms
Source: Nat Commun. 2019 Oct 22;10:4799. doi: 10.1038/s41467-019-12793-3 (PMC6805915; doi:10.1038/s41467-019-12793-3)
Supplement: Supplementary file 2 — Peer Review [file 41467_2019_12793_MOESM2_ESM.pdf]

Reviewers' comments:

Reviewer #1 (Remarks to the Author):

The studies presented in this manuscript demonstrate a multigenerational increase in memory impairment in offspring of female mice with HFD-induced insulin resistance prior to and during pregnancy and attempt to elucidate the molecular mechanisms underlying these deficits. They show the memory impairment is tightly correlated with decreases in expression and specific promoter activity of BDNF both within the hippocampus and germline and can be reversed by a novel enriched environment.

The key findings are:
1) Both male and female offspring of female mice fed HFD peripartum show impaired hippocampal learning and memory as measured by various parameters of Morris Water Maze and novel object recognition, and correlated LTP in hippocampal slices.
2) These impairments were NOT associated with changes in basic metabolic parameters of these mice, suggesting they are not a result of alterations in metabolic homeostasis.
3) Similar impairments persist in F2 and F3 generations from male progenitors.
4) RT-PCR of hippocampal extracts for a number of genes regulating synaptic plasticity showed specific and persistent decreases in expression of Bdnf exons I, IV, and IX, with associated decreases in BDNF protein levels and decreased enrichment of H3K9ac and H3K4me3 at each isoform's promoter in both hippocampus and germline.
5) In F2 male mice, authors show that exposure to a novel enriched environment can partially revert hippocampal learning deficit and BDNF protein levels, with associated reversion H3K9ac/H3K4me3 enrichment at Bdnf promoters.
6) Peripartum exposure to HFD is associated with alterations in insulin signaling (IRS1), and nutrient sensing protein phosphorylation (CREB/FOXO3a, and SIRT) in gonads of HFD-exposed females, which in turn is associated with increased FOXO3a association with both the histone acetylase SIRT2 and the histone deacetylase HDAC2 and altered recruitment of these histone modifiers to the Bdnf locus.

The data presented are very clean and well-executed, and provide the novel observation that HFD-induced memory impairments can be transmitted transgenerationally. The data the authors provide re: mechanism, namely germline modification of histones at the Bdnf locus via alterations in Foxo3a-mediated histone modifiers is descriptive and correlative and does not provide any definitive testing of the mechanisms they hypothesize. Importantly, the authors do not provide a definitive experimental perturbation that demonstrates that any of their observations re: mechanism are causal for the transgenerational phenotype they observe.

Major concerns:
1) The title of their manuscript suggests the transgenerational phenotypes are due to changes in germline alterations in histone acetylation. This conclusion rests on a single set of data (ChIP-PCR on germline cells in F1-F3 males) which needs better controls (see below). In order to definitively prove this, the authors would need to either specifically induce (without HFD) or prevent these changes and examine phenotypes. Although this is certainly a high bar, it is necessary to support their stated conclusions. For instance, authors posit that insulin resistance in the ovary initiates the changes at Bdnf locus. Ovary-specific IRS1 knockout would be predicted to phenocopy and would demonstrate causation rather than correlation. Similarly there would be a number of ways to genetically preserve insulin sensitivity in ovary in the presence of HFD. This would show the necessity of ovarian insulin resistance in transgenerational effects on memory. On an associated note, given that H3K4me3 and H3K9ac enrichment are generally correlated with transcriptional expression, it is unclear if these alterations are a cause or effect of altered expression (the authors do not present any transcriptional data from germline to go with their ChIP-seq, Is this because Bdnf is not expressed in germline?)
2) It is completely unclear how the NEE data fit into the narrative of germline inheritance of Bdnf promoter alterations. It has already been established by many other groups that an enriched

environment can increase Bdnf expression in hippocampus with associated alterations in the epigenome (see PMID 20232397 for one of many examples). So the reversion of some of the observed changes caused by HFD are not surprising and do not relate to germline transmission in a substantive way (i.e, the changes could be additive). At least, the authors should effects of NEE on germline changes and subsequent effects on memory in the next generation in order to really fit. Whatever result they get would be of interest to the field.
3) All ChIP-seq data shows decreased enrichment at the Bdnf locus at multiple promoter sites. The authors should provide some negative controls at other sites (ideally other Bdnf promoters where expression does not change) as controls to ensure that there isn't a normalization or technical issue that leads to these results.

Minor concerns:
1) Authors show that weight, glucose, insulin, and food intake are unchanged in SD vs HFD offspring in order to suggest that observed phenotypes/histone changes are not a consequence of some metabolic phenotype. Although this is important, a) these are extremely gross estimates of metabolic health. ITT or GTTs at minimum to show insulin sensitivity are more sensitive measures of metabolic health than the measures they chose. and b) other unmeasured alterations (paternal behaviors, sperm carriage of epigenetic memory (see Oliver Rando's work) could still be the cause. I think it's important for authors to acknowledge such possibilities in their interpretation of data and their conclusions, even if they cannot definitively prove their mechanism as I cover inmajor concern #1.
2) Should show in Fig. 5, LTP correlates for completion, rescue of qPCR (increase in protein levels could be nontranscriptional),
3) In NEE experiments (Fig. 5), it would be ideal to show NEE effects on SD exposed offspring as well. If NEE shows similar changes from a different baseline, the interpretation would be very different than if in SD offspring, there are no further increases in histone enrichment/Bdnf expression.
4) When paired for breeding, was male exposed to HFD during pairing, and if so for how long? Are male mice removed after preganancy? Please make this clear in methods.
5) Fig 4a: Unclear what the point of the heat map is, especially if not going to cluster genes or label them.

Suggestion:
Given changes that they observe in HDAC2 and SIRT2 association with Foxo3a, performing ChIP-seq for decreases in H3K4me3 or H3K9ac to a) look for enrichment of Foxo3a motifs and b) examine for other potential epigenetic targets of exposure to HFD would both buttress their mechanistic hypothesis and provide further targets for study by the authors as well as others interested in how HFD may change the epigenome.

Reviewer #2 (Remarks to the Author):

Fusco et al. report the impact of maternal high fat diet (before and during pregnancy) on the hippocampal learning/memory and LTP of F1 offspring and that these phenotypes are transmitted to the F2 and F3 generations through the paternal line. Maternal high fat diet has previously been reported to affect the cognitive performance of the F1 offspring, but Fusco et al. in the current manuscript extend this line of investigation to the F2 and F3 generations. The behavioral and LTP data in F1, F2 and F3 are convincing and the presence of the phenotypes in the F3 generation and lack of metabolic alterations in the F1 indicate a genuine transgenerational transmission of the cognitive phenotype. The authors tend to connect insulin resistance in mothers to the offspring phenotype, but insulin resistance, as a cause for the phenotypes, has not been studied; thus only the high fat diet can be linked to the phenotypes.
The authors also demonstrate that enriched environment of F1 males prevents the development of the cognitive deficit in F2. They refer to this effect as a reversal of the transgenerational transmission, but

they have not continued breeding up to F3 and F4; thus this effect is intergenerational only.
In an attempt to explain the hippocampal phenotypes (memory and LTP), the authors profiled neuroplasticity-related genes from hippocampal extracts. This approach is not ideal because neurons and glial cells and neuronal subtypes were not separated and only a limited number of genes were profiled. Current technologies are available for generating cell type specific transcriptomes. Nevertheless, maternal high fat induced reduction in BDNF expression across the 3 generations. This reduction was accompanied by reduced transcription from BDNF promoters I, IV, and IXa, as well as reduction in active promoter-specific histone marks at these promoters. The data consistently show changes across generations, but no attempt was made to causally link the epigenetic and expression changes to the behavioral phenotype.
Finally, the authors detect similar expression and epigenetic changes in the ovaries of F0 mothers on high fat diet. Germ cells were not profiled, and the signal is likely coming from non-germ cells. It is not clear, and the authors do not speculate on, how these ovarian changes can be linked to the transgenerational inheritance of the cognitive deficit. This experiment does not add a lot to the work and does not provide a mechanistic understanding of transgenerational transmission.
Overall, this paper reports a large amount of (nice) behavioral, expression, and epigenetic data, but without demonstrating causality or providing a mechanistic insight into transgenerational transmission of behavior. Recent work in several laboratories elucidated somatic and gametic mechanisms of multigenerational transmission of behavior and it would be important to demonstrate if the transmission described in this manuscript is related to some of these mechanism or represents a novel mechanism.

Reviewer #3 (Remarks to the Author):

Fusco et al. report that high fat diet (HFD) in female mice impairs learning and memory and LTP in the offspring F1, F2 and F3 generation. This observation is linked to decreased hippocampal BNDF signaling. The data is interesting but considering that by now there are numerous studies showing that environmental factors impact on memory function in a transgenerational manner the key observation is only another report of an interesting phenomena that adds to this field but represents no major progress in mechanistic understanding. As such, the question regarding the underlying mechanism becomes more important in my view. The study does not employ the state-of the art tools already available in the field and the link to BDNF remains correlative. At present I feel the data is too preliminary and not competitive for a publication in Nature Communications.

Major issues:
1.
The authors describe an interesting phenotype that HFD impairs memory in subsequent generations. The link to BNDF signaling via the epigenetic regulation of the BNDF gene is correlative. The rescue experiments using novelty environmental enrichment in the F2 generation are not conclusive. Enrichment improves memory consolidation in general, an effect that would be obvious if the authors would have shown an SD-NEE control group that is currently missing.
Other treatments that improve memory will work similarly. One way to address this question in part could be to specifically increase BNDF levels in the F0 generation and then study the behavioral and molecular phenotypes in F1, F2 and F3 - and repeat this experiment starting with manipulating F1.

2.
Related to point 1 it is surprising that HFD only affect oocytes in F0. How is the signal transmitted alter only via the male offspring. The data suggest via sperm but I understand that the data shown in Fig 4e refers to germline and there is no description in the methods how “gonads” were isolated. It would be helpful to show that the differences occur in isolated sperm free of other cell types.

3.
A number of recent studies in the field now routinely use in vitro fertilization (IVF) or similar techniques such a injection of fertilized oocytes. The authors cannot rule out that the effect of HFD is mediated via changes in maternal behavior. Cross fostering and IVF using for example sperm from F1 is therefore essential.

4.
Why were male descendants only analyzed in the NOR task and not for LTP and water maze. NOR is the least sensitive of the employed test and more subtle changes may only be detectable by LTP and spatial reference memory assessment?

5.
It would be important if the 10 animals/group used for the experiments depicted in Fig 2a-c and the slice used for electrophysiological analysis in Fig 2D from example stem from 10 different litters. If not, I think the data is statistically not sound. With other words the number of litters should be considered as independent "n" and not the total number of offspring.

6.
Recent data suggest that novelty enriched environment induces transgenerational effects and improves memory in offspring via RNA-dependent mechanisms. It is thus likely that the BNDF regulation seen in this study is an epi-phenomenon. The authors should comment on this. Moreover, would enrichment of F2 also improve memory in F3?

7.
The authors conduct time consuming experiments and then limit themselves to the analysis of a few selected genes via qPCR array and eventually link their finding to BNDF, "certainly a low hanging fruit". While the idea to study gene-expression as a molecular correlate and starting point to understand the transgenerational effects in the study in timely, the approach is limited. At least RNA-seq should be performed to obtain a genome-wide view on HDF induced changes in gonads (sperm and oocytes) of F0 and brains of F1 and at least F2.

8.
The authors suggest that increased recruitment of HDAC2 to the BDNF promoter in gonads of HFD mother leads to lower BNDF expression in the brain of offspring. How is this phenomena than transmitted to F2 and F3? Is sperm in F1 and F2 affected by a similar mechanisms…and fi so how is this mediated?

9
Is any tissue that expresses BNDF affected similarly or is the effect specific to gonads and hippocampus?

10.
Related to point 10 assuming that BNDF signaling – in addition to other processes – may be affected at the body wide level: While weight, food intake and plasma glucose levels are similar in F1 generation the authors should confirm that motorbehavior and other processes that may affect memory function are not altered in the F1-F3 generation.

Manuscript NCOMMS-18-18649A

POINT-BY-POINT RESPONSE TO THE REFEREES' COMMENTS

RESPONSE TO REVIEWER #1

The studies presented in this manuscript demonstrate a multigenerational increase in memory impairment in offspring of female mice with HFD-induced insulin resistance prior to and during pregnancy and attempt to elucidate the molecular mechanisms underlying these deficits. They show the memory impairment is tightly correlated with decreases in expression and specific promoter activity of BDNF both within the hippocampus and germline and can be reversed by a novel enriched environment.

The key findings are:

1) Both male and female offspring of female mice fed HFD peripartum show impaired hippocampal learning and memory as measured by various parameters of Morris Water Maze and novel object recognition, and correlated LTP in hippocampal slices.

2) These impairments were NOT associated with changes in basic metabolic parameters of these mice, suggesting they are not a result of alterations in metabolic homeostasis.

3) Similar impairments persist in F2 and F3 generations from male progenitors.

4) RT-PCR of hippocampal extracts for a number of genes regulating synaptic plasticity showed specific and persistent decreases in expression of Bdnf exons I, IV, and IX, with associated decreases in BDNF protein levels and decreased enrichment of H3K9ac and H3K4me3 at each isoform's promoter in both hippocampus and germline.

5) In F2 male mice, authors show that exposure to a novel enriched environment can partially revert hippocampal learning deficit and BDNF protein levels, with associated reversion H3K9ac/H3K4me3 enrichment at Bdnf promoters.

6) Peripartum exposure to HFD is associated with alterations in insulin signaling (IRS1), and nutrient sensing protein phosphorylation (CREB/FOXO3a, and SIRT) in gonads of HFD-exposed females, which in turn is associated with increased FOXO3a association with both the histone acetylase SIRT2 and the histone deacetylase HDAC2 and altered recruitment of these histone modifiers to the Bdnf locus.

The data presented are very clean and well-executed, and provide the novel observation that HFD-induced memory impairments can be transmitted transgenerationally. The data the authors provide re: mechanism, namely germline modification of histones at the Bdnf locus via alterations in Foxo3a-mediated histone modifiers is descriptive and correlative and does not provide any definitive testing of the mechanisms they hypothesize. Importantly, the authors do not provide a definitive experimental perturbation that demonstrates that any of their observations re: mechanism are causal for the transgenerational phenotype they observe.

>> (Response) **We are very grateful to the referee for his/her comments and suggestions that were very useful to improve our manuscript.**

Major concerns:

1) The title of their manuscript suggests the transgenerational phenotypes are due to changes in germline alterations in histone acetylation. This conclusion rests on a single set of data (ChIP-PCR on germline cells in F1-F3 males) which needs better controls (see below). In order to definitively prove this, the authors would need to either specifically induce (without HFD) or prevent these changes and examine phenotypes. Although this is certainly a high bar, it is necessary to support their stated conclusions. For instance, authors posit that insulin resistance in the ovary initiates the changes at Bdnf locus. Ovary-specific IRS1 knockout would be predicted to phenocopy and would demonstrate causation rather than correlation. Similarly there would be a number of ways to genetically preserve insulin sensitivity in ovary in the presence of HFD. This would show the necessity of ovarian insulin resistance in transgenerational effects on memory.

>> (Response) **We followed the reviewer's suggestion to genetically preserve insulin sensitivity in ovary in the presence of HFD by taking advantage of the p66Shc knock-out mouse. p66Shc is one of the three isoforms of the adaptor protein family ShcA, which is critically involved in the insulin signaling cascade (Ranieri et al., 2010 and 2013). Deletion of p66shc has been demonstrated to be protective in experimental models of diabetes-dependent nephropathy and cardiomyopathy (Menini et al., 2006; Rota et al., 2006; Sun et al., 2010). In addition, Ranieri et al. found that p66 deficiency induced a significant protective effect in lepOb/Ob mice, an established genetic model of obesity and insulin resistance. Strikingly, p66Shc inactivation improved insulin signaling in white fat without affecting (hyper)insulinaemia and independently of body weight. More importantly, the same study demonstrated that p66Shc interacted with IRS1 and promoted its inhibitory phosphorylation on serine 307. Given these premises, we hypothesized that p66Shc deficiency might counteract the HFD-induced IRS1 hyperphoshorylation in the ovaries and abolish the insulin resistance-dependent epigenetic changes on *Bdnf* promoters of HFD descendants. Our hypothesis was confirmed by the following results: i) upon HFD dietary regimen F0 p66Shc KO females showed metabolic alterations resembling the insulin resistance phenotype observed in wild-type mice (Supplementary Fig. 6b,c and Fig. 1b-e); ii) however, no significant changes of $IRS1^{Ser307}$ phosphorylation were found in their ovaries after four weeks of HFD compared to F0 SD p66Shc KO (Fig. 8a). Remarkably, the offspring generated from HFD-fed p66Shc KO females showed cognitive performances comparable to those of mice born from SD-fed mice in both NOR and Morris water maze tasks (Fig. 8b,c). Accordingly, LTP recordings at the CA3–CA1 synapses were not significantly different between $F1_{HFD}$ and SD p66Shc KO mice (fEPSP amplitude: 55.6±13.1% vs. 46±5.4%, p = 0.46; fEPSP slope: 59.7±12.2% vs. 45.2±4.8%, p = 0.23, n = 10-12 slices for each group). Of note, epigenetic activation of *Bdnf* promoters I, IV and IX was not significantly different between $F1_{HFD}$ and SD mice in both hippocampus and germline of p66Shc KO animals (Fig. 8e,f). Finally, BDNF levels did not significantly change in the hippocampus of $F1_{HFD}$ p66Shc KO mice (Fig. 8d).**

On an associated note, given thatH3K4me3 and H3K9ac enrichment are generally correlated with transcriptional expression, it is unclear if these alterations are a cause or effect of altered expression (the authors do not present any transcriptional data from germline to go with their ChIP-seq, Is this because Bdnf is not expressed in germline?)

>> (Response) **Bdnf expression was analyzed in the germline of $F1_{HFD}$ mice and it was significantly reduced (Supplementary Fig. 3c) as observed in other tissues (Supplementary Fig. 3d).**

2) It is completely unclear how the NEE data fit into the narrative of germline inheritance of Bdnf promoter alterations. It has already been established by many other groups that an enriched environment can increase Bdnf expression in hippocampus with associated alterations in the epigenome (see PMID 20232397 for one of many examples). So the reversion of some of the observed changes caused by HFD are not surprising and do not relate to germline transmission in a substantive way (i.e, the changes could be additive). At least, the

authors should effects of NEE on germline changes and subsequent effects on memory in the next generation in order to really fit. Whatever result they get would be of interest to the field.

>> (Response) **There was probably a misunderstanding. As shown in Fig. 1, $F1_{HFD}$ male mice (born from HFD-fed mothers) were exposed to NEE, and then bred with control females to generate $F2_{HFD\ NEE}$ mice. Therefore, the mice we tested (i.e., $F2_{HFD\ NEE}$ mice) were not directly exposed to either HFD or NEE. Consequently, the outcomes of both HFD and NEE on cognitive functions have to be considered as inter/multigenerational effects. For more details, see also our response to concern #1 of Reviewer #3.**

Moreover, behavioral analysis performed on the next generation ($F3_{HFD\ NEE}$) still showed better cognitive performances than $F3_{HFD}$ mice (Supplementary Fig. 5b). These novel findings suggest that the rescuing effects of NEE against HFD-induced damage is transgenerationally transmitted.

Finally, ChIP analyses carried out on the germline of HFD descendants revealed that NEE almost completely restored epigenetic activation of *Bdnf* promoters in both mice directly exposed ($F1_{HFD}$ NEE) and descendants ($F2_{HFD\ NEE}$) (Supplementary Fig. 5a and Fig. 5g), suggesting a link between the NEE effects and the gametic transmission of the phenotype.

3) All ChIP-seq data shows decreased enrichment at the Bdnf locus at multiple promoter sites. The authors should provide some negative controls at other sites (ideally other Bdnf promoters where expression does not change) as controls to ensure that there isn't a normalization or technical issue that leads to these results.

>> (Response) **ChIP analysis performed on the promoters III and VI showed no significant modifications of H3K9ac and H3K4me3 on these regulatory sequences (Supplementary Fig. 3e), according to the exon-specific expression changes of *Bdnf* shown in Fig. 4b.**

<u>**Minor concerns**</u>:

1) Authors show that weight, glucose, insulin, and food intake are unchanged in SD vs HFD offspring in order to suggest that observed phenotypes/histone changes are not a consequence of some metabolic phenotype. Although this is important, a) these are extremely gross estimates of metabolic health. ITT or GTTs at minimum to show insulin sensitivity are more sensitive measures of metabolic health than the measures they chose. …and b) other unmeasured alterations (paternal behaviors, sperm carriage of epigenetic memory (see Oliver Rando's work) could still be the cause. I think it's important for authors to acknowledge such possibilities in their interpretation of data and their conclusions, even if they cannot definitively prove their mechanism as I cover in major concern #1.

>> (Response) **We performed intraperitoneal GTT in all generations of HFD descendants. Our results confirmed no differences regarding insulin sensitivity in HFD progeny (Supplementary Fig. 1a and Supplementary Fig. 2h). We do not exclude that other metabolic changes or unmeasured alterations might occur in our experimental model though several precautions we used (e.g. males were removed from the female's cage 1-2 days after mating) and, most importantly, the results of new IVF and cross fostering experiments (see response to comment #3 of Reviewer #3) allowed us to rule out some of these variables. Nonetheless, according to the reviewer's suggestion, we integrated our discussion by mentioning other mechanisms potentially involved in the transgenerational transmission of the observed phenotype (see page 22, lines 482-487 and page 24, lines 533-537).**

2) Should show in Fig. 5, LTP correlates for completion, rescue of qPCR (increase in protein levels could be nontranscriptional).

>> (Response) **Figure 5 has been implemented with LTP and qPCR data. Our findings indicate that NEE restores hippocampal synaptic plasticity similarly to learning and memory (Fig. 5c,d), and the rescue of BDNF expression likely occurs at transcriptional level (Fig. 5e).**

3) In NEE experiments (Fig. 5), it would be ideal to show NEE effects on SD exposed offspring as well. If NEE shows similar changes from a different baseline, the interpretation would be very different than if in SD offspring, there are no further increases in histone enrichment/Bdnf expression.

>> (Response) **The revised Fig. 5 has been integrated with the results of behavioral, electrophysiological and molecular analyses performed on the descendants from mice fed with standard diet and exposed to novel enriched environment ($F2_{SD\ NEE}$ mice). Behavioral, electrophysiological and molecular analyses performed on $F2_{SD\ NEE}$ mice revealed no significant changes compared to SD mice, thus indicating that in our experimental model NEE intergenerational effects only occur in HFD experimental condition.**

4) When paired for breeding, was male exposed to HFD during pairing, and if so for how long? Are male mice removed after preganancy? Please make this clear in methods.

>> (Response) **Male mice were removed from the female's cage 1-2 days after mating and were exposed to HFD only during this time lapse. This information has been added to "Methods" (see page 26, lines 578-580).**

5) Fig 4a: Unclear what the point of the heat map is, especially if not going to cluster genes or label them.

>> (Response) **Based on this reviewer's comment we removed the heat map from Fig. 4 and we only left the table with genes whose expression was significantly changed in the hippocampus of HFD descendants. A whole table with the description of all analyzed genes and the fold induction results is shown in the Supplementary Table 1.**

Suggestion:

Given changes that they observe in HDAC2 and SIRT2 association with Foxo3a, performing ChIP-seq for decreases in H3K4me3 or H3K9ac to a) look for enrichment of Foxo3a motifs and b) examine for other potential epigenetic targets of exposure to HFD would both buttress their mechanistic hypothesis and provide further targets for study by the authors as well as others interested in how HFD may change the epigenome.

>> (Response) **We thank the reviewer for the very intriguing suggestion. We agree that a broad spectrum analysis of HFD effects on epigenome might be very interesting and we definitely planned to perform it in an independent follow-up study.**

RESPONSE TO REVIEWER #2

Fusco et al. report the impact of maternal high fat diet (before and during pregnancy) on the hippocampal learning/memory and LTP of F1 offspring and that these phenotypes are transmitted to the F2 and F3 generations through the paternal line. Maternal high fat diet has previously been reported to affect the cognitive performance of the F1 offspring, but Fusco et al. in the current manuscript extend this line of investigation to the F2 and F3 generations. The behavioral and LTP data in F1, F2 and F3 are convincing and the presence of the phenotypes in the F3 generation and lack of metabolic alterations in the F1 indicate a genuine transgenerational transmission of the cognitive phenotype. The authors tend to connect insulin resistance in mothers to the offspring phenotype, but insulin resistance, as a cause for the phenotypes, has not been studied; thus only the high fat diet can be linked to the phenotypes.

>> (Response) **We wish to thank this Referee for kind words of appreciation of our work. In the revised manuscript we added data obtained from p66Shc KO mice indicating that ovarian insulin resistance plays a critical role in producing the phenotype (Fig. 8). The results of these new experiments are summarized in our response to major concern #1 of Reviewer #1.**

The authors also demonstrate that enriched environment of F1 males prevents the development of the cognitive deficit in F2. They refer to this effect as a reversal of the transgenerational transmission, but they have not continued breeding up to F3 and F4; thus this effect is intergenerational only.

>> (Response) **In the revised manuscript we added data obtained from behavioral analyses performed in $F3_{HFD\ NEE}$ mice (Suppl. Fig. 5b). Considering that exposure to NEE is limited to $F1_{HFD}$ mice and it occurs before the conception of $F2_{HFD\ NEE}$ mice, the presence of the phenotype in the next two generations (i.e., $F2_{HFD\ NEE}$ and $F3_{HFD\ NEE}$ mice) points to transgenerational inheritance (Klengel et al., 2016).**

In an attempt to explain the hippocampal phenotypes (memory and LTP), the authors profiled neuroplasticity-related genes from hippocampal extracts. This approach is not ideal because neurons and glial cells and neuronal subtypes were not separated and only a limited number of genes were profiled. Current technologies are available for generating cell type specific transcriptomes.

>> (Response) **To investigate neuron-specific expression of *Bdnf* exons, we performed qPCR analyses on single-cell samples collected from CA1 neurons in acute hippocampal brain slices of SD and $F1_{HFD}$ mice by whole-cell patch-clamp technique. Data obtained by digital PCR confirmed lower levels of *Bdnf* expression in hippocampal neurons of $F1_{HFD}$ mice (Supplementary Fig. 3a,b). However, we plan to perform a broader spectrum analysis of gene expression in hippocampal neurons of HFD descendants as independent follow-up study.**

Nevertheless, maternal high fat induced reduction in BDNF expression across the 3 generations. This reduction was accompanied by reduced transcription from BDNF promoters I, IV, and IXa, as well as reduction in active promoter-specific histone marks at these promoters. The data consistently show changes across generations, but no attempt was made to causally link the epigenetic and expression changes to the behavioral phenotype.

>> (Response) **The authors acknowledge that the manuscript does not provide *direct* experimental evidence causally linking alterations identified in HFD descendants to the observed phenotype. Nonetheless, the results of several new experiments contained in the revised manuscript confirmed that**

epigenetic inhibition of *Bdnf* expression correlated with the transmission of cognitive impairment (e.g., cross fostering and *in vitro* fertilization (IVF) experiments, Supplementary Fig. 4; see also response to concern #3 of Reviewer #3), whereas experimental models counteracting the multigenerational inheritance of cognitive deficits consistently showed a rescue of neurotrophic factor expression (NEE, maternal BDNF injection and p66Shc KO, Figs. 5, 7 and 8, respectively). However, we do not exclude that expression of other genes critically affecting synaptic plasticity might be differentially regulated or that other epigenetic mechanisms (e.g. microRNAs, DNA methylation) could play a role in the transgenerational transmission of the phenotype. We included these speculations in the Discussion of the revised manuscript (see page 25, lines 549-553).

Finally, the authors detect similar expression and epigenetic changes in the ovaries of F0 mothers on high fat diet. Germ cells were not profiled, and the signal is likely coming from non-germ cells. It is not clear, and the authors do not speculate on, how these ovarian changes can be linked to the transgenerational inheritance of the cognitive deficit. This experiment does not add a lot to the work and does not provide a mechanistic understanding of transgenerational transmission.

>> (Response) **Molecular analyses of F0 mothers were performed on mechanically isolated ovarian follicles. A description of the procedure has been added to Methods (page 29, lines 665-668). We agree with the reviewer that the molecular changes we found may arise from both germ and non-germ cells. However, epigenetic changes of germ cells are the only modifications potentially transmittable to the next generations. Otherwise, we should hypothesize a non-gametic transfer involving maternal behavior or other mechanisms (e.g. humoral factors). Novel data included in the revised manuscript about cross fostering and IVF experiments added a significant layer to the interpretation of our experimental model. We also included some of these considerations in the revised manuscript. In particular, we speculate that alteration of both insulin receptor and TrkB signaling in the ovary may trigger epigenetic changes potentially stable during oocyte growth and after fertilization. Consequently, the acquisition of epigenetic marks in the germline of embryo would lead to the transgenerational transmission of the Bdnf downregulation (page 24, lines 530-539).**

Overall, this paper reports a large amount of (nice) behavioral, expression, and epigenetic data, but without demonstrating causality or providing a mechanistic insight into transgenerational transmission of behavior. Recent work in several laboratories elucidated somatic and gametic mechanisms of multigenerational transmission of behavior and it would be important to demonstrate if the transmission described in this manuscript is related to some of these mechanism or represents a novel mechanism.

>> (Response) **Novel findings collected from the p66Shc KO mouse model (Fig. 8) revealed the causative role of ovarian insulin resistance in the HFD-dependent transgenerational transmission of both cognitive deficits and Bdnf inhibition. Several works demonstrated that non-gametic inheritance may occur via different mechanisms including parental hormones, cytokines or microorganisms (Howerton and Bale, 2012; Toth, 2015). However, the results of both IVF and cross-fostering experiments included in the revised manuscript (Supplementary Fig. 4) suggest that the transgenerational inheritance of our experimental phenotype is transmitted through a gametic mechanism.**

RESPONSE TO REVIEWER #3

Fusco et al. report that high fat diet (HFD) in female mice impairs learning and memory and LTP in the offspring F1, F2 and F3 generation. This observation is linked to decreased hippocampal BNDF signaling. The data is interesting but considering that by now there are numerous studies showing that environmental factors impact on memory function in a transgenerational manner the key observation is only another report of an interesting phenomena that adds to this field but represents no major progress in mechanistic understanding. As such, the question regarding the underlying mechanism becomes more important in my view. The study does not employ the state-of the art tools already available in the field and the link to BDNF remains correlative. At present I feel the data is too preliminary and not competitive for a publication in Nature Communications.

>> (Response) **The revised manuscript contains the results of many new experiments designed to address the Reviewers' concerns. Our new findings hopefully provide mechanistic insight into the transgenerational transmission of HFD-dependent cognitive impairment.**

<u>Major issues</u>:

1. The authors describe an interesting phenotype that HFD impairs memory in subsequent generations. The link to BNDF signaling via the epigenetic regulation of the BNDF gene is correlative. The rescue experiments using novelty environmental enrichment in the F2 generation are not conclusive. Enrichment improves memory consolidation in general, an effect that would be obvious if the authors would have shown an SD-NEE control group that is currently missing.

>> (Response) **First, we would like to draw the reviewer's attention on the experimental model described in Fig. 1. Specifically, $F1_{HFD}$ male mice (born from F0 HFD-fed females) were exposed to NEE, then bred with control females to generate $F2_{HFD\ NEE}$ mice. Therefore, the mice we tested (i.e., $F2_{HFD\ NEE}$ mice) <u>had not been directly exposed to NEE</u>. We also performed behavioral, electrophysiological and molecular analyses of descendants from mice fed with standard diet and exposed to novel enriched environment (named $F2_{SD\ NEE}$). The results of these new experiments, shown in the revised Fig. 5, indicate that, in standard diet condition, paternal exposure to NEE did not significantly change hippocampal synaptic plasticity, learning and memory of descendants. Conversely, when the progenitor (F0) fed HFD, paternal ($F1_{HFD}$) exposure to NEE broke the transgenerational transmission of cognitive impairment to the next generations ($F2_{HFD\ NEE}$ and $F3_{HFD\ NEE}$; Fig. 5a,b and Supplementary Fig. 5b) by restoring epigenetic activation of *Bdnf* promoters in the germline of mice exposed to NEE ($F1_{HFD}$ NEE; Supplementary Fig. 5a).**

Other treatments that improve memory will work similarly. One way to address this question in part could be to specifically increase BNDF levels in the F0 generation and then study the behavioral and molecular phenotypes in F1, F2 and F3 - and repeat this experiment starting with manipulating F1.

>> (Response) **Following the Reviewer's suggestion we intraperitoneally injected F0 HFD-fed female mice with BDNF (3 times per week for four weeks) as long as they fed HFD until the breeding (hereinafter named F0 HFD BDNF). This protocol of BDNF administration was designed to restore the BDNF/TrkB signaling in ovaries (Fig. 7a) without interfering with embryo development after conception. The offspring of F0 HFD BDNF females (i.e., $F1_{HFD\ BDNF}$ mice) showed learning and memory significantly greater than those of $F1_{HFD}$ mice (Fig. 7c,d). Accordingly, both epigenetic activation and expression of *Bdnf* were almost completely restored in the hippocampus of $F1_{HFD\ BDNF}$ mice (Fig. 7e,f).**

However, it is worth mentioning that BDNF administration exerted anorectic effects on HFD-fed mice, as indicated by both reduced calorie intake and weight gain of F0 HFD BDNF females (Fig. 7b). Nevertheless, ovaries of F0 HFD BDNF female mice showed higher levels of IRS1^{S307} phosphorylation than control mice, suggesting that HFD dietary regimen had affected ovarian insulin signaling. Our interpretation of these findings is that both the direct effects of BDNF on ovaries and attenuation of maternal insulin resistance may contribute to the lack of intergenerational effects on cognitive functions in this experimental model. These considerations have been included in the "Discussion" of the revised manuscript at page 24, lines 529-532.

2. Related to point 1 it is surprising that HFD only affect oocytes in F0. How is the signal transmitted alter only via the male offspring. The data suggest via sperm but I understand that the data shown in Fig 4e refers to germline and there is no description in the methods how "gonads" were isolated. It would be helpful to show that the differences occur in isolated sperm free of other cell types.

>> (Response) **We apologize for missing information in our previous submission. ChIP experiments were performed on mouse sperm (free of other cell types) obtained from the cauda epididymis. A description of the procedure has been added to "Methods" (page 29, lines 661-665).**

3. A number of recent studies in the field now routinely use in vitro fertilization (IVF) or similar techniques such a injection of fertilized oocytes. The authors cannot rule out that the effect of HFD is mediated via changes in maternal behavior. **Cross fostering** and **IVF** using for example sperm from F1 is therefore essential.

>> (Response) **We thank the reviewer for this very useful comment that prompted us to perform new experiments whose results strengthened our conclusions. To exclude changes in maternal behavior, we performed cross fostering experiments by removing $F1_{SD}$ and $F1_{HFD}$ pups and transferring them to other lactating dams (fed with SD). $F1_{HFD}$ mice fostered by control females (hereinafter named $F1_{HFD}$ CF) still showed memory impairment and lower *Bdnf* expression in their hippocampi (Supplementary Fig. 4a,b). Accordingly, *Bdnf* gene was epigenetically inhibited in both hippocampus and germline of $F1_{HFD}$ CF mice (Supplementary Fig. 4c,d).**

In addition, to investigate if gametic or non-gametic mechanisms underlie the multigenerational transmission of phenotype, we performed IVF experiments by fertilizing germ cells obtained from control females with sperm collected from novel cohorts of $F1_{SD}$ and $F1_{HFD}$ mice. Mice generated by IVF with sperm collected from $F1_{HFD}$ mice (hereinafter named $F2_{HFD}$ IVF) showed both cognitive deficit and molecular changes resembling those observed in HFD descendants. (Supplementary Fig. 4e-g). Collectively, these experimental models rule out the contribution of maternal behavior and corroborate our contention that gametic mechanisms underlie transmission of the phenotype.

4. Why were male descendants only analyzed in the NOR task and not for LTP and water maze. NOR is the least sensitive of the employed test and more subtle changes may only be detectable by LTP and spatial reference memory assessment?

>> (Response) **Probably, there was a misunderstanding. All HFD descendants were studied by NOR, Morris water maze (MWM) and LTP analysis (Fig. 2 and Fig. 3). $F2_{HFD\ NEE}$ mice were originally tested by MWM and data from LTP experiments have been added to the revised manuscript (Fig. 5). We limited the behavioral analysis to NOR only for supplementary data performed on both female HFD descendants and male HFD descendants obtained from mothers whose HFD was stopped during lactation (Supplementary Fig. 2a,b). However, in both latter groups cognitive impairment was also detectable by the less sensitive NOR test.**

5. It would be important if the 10 animals/group used for the experiments depicted in Fig 2a-c and the slice used for electrophysiological analysis in Fig 2D from example stem from 10 different litters. If not, I think the data is statistically not sound. With other words the number of litters should be considered as independent "n" and not the total number of offspring.

>> (Response) **First, we want to clarify that mice used for behavioral tests were different from those studied for electrophysiological and molecular analyses. Moreover, the number of litters from which the analyzed animals were derived has been now indicated in the figure legends of the revised manuscript. Despite our study has a "between litter experimental design", statistical analyses were performed by considering as independent "n" the number of mice (obtained from different litters) for behavioral and molecular analyses, and the number of slices for electrophysiological analyses, as reported in previous works (Tozuka et al., Faseb J 2009; Suter et al., Faseb J 2009; Yang et al., Journal of Molecular Endocrinology 2012). To minimize variability among litters, we adopted several precautions: i) female mice belonging to the same litter were randomly assigned to the two experimental groups , i.e., SD and HFD; ii) the same male mouse was paired, at different times, with both a F0 SD female and a F0 HFD female mouse; iii) for each experimental set, the analyzed mice deriving from the same litter were never more than two; iv) litters with less than five or more than eight pups were excluded from the study. Finally, we want to emphasize that cross fostering should reduce the "litter effects", and $F1_{HFD}$ mice fostered by control females ($F1_{HFD}$ CF mice) still showed behavioral and molecular changes similar to those observed in $F1_{HFD}$ mice (Supplementary Fig. 4a-d).**

6. Recent data suggest that novelty enriched environment induces transgenerational effects and improves memory in offspring via RNA-dependent mechanisms. It is thus likely that the BNDF regulation seen in this study is an epi-phenomenon. The authors should comment on this. Moreover, would enrichment of F2 also improve memory in F3?

>> (Response) **Following the suggestions of both Reviewers 1 and 3, we implemented our experimental design by also analyzing descendants from mice fed with SD and exposed to NEE. Our new data indicate that paternal exposure to NEE did not modify synaptic plasticity, learning and memory of descendants in standard diet condition but it almost completely restored cognitive performances, LTP recordings and *Bdnf* expression only if the progenitor fed HFD (Fig. 5). Our new data also show that NEE changed epigenetic markers on *Bdnf* promoters only in the germline of mice born to HFD-fed mothers (Supplementary Fig. 5a). Finally, as requested, we performed behavioral evaluation of $F3_{HFD\ NEE}$ mice, indicating that NEE rescue was transgenerationally transmitted (see revised Supplementary Fig. 5b).**

7. The authors conduct time consuming experiments and then limit themselves to the analysis of a few selected genes via qPCR array and eventually link their finding to BNDF, "certainly a low hanging fruit". While the idea to study gene-expression as a molecular correlate and starting point to understand the transgenerational effects in the study in timely, the approach is limited. At least RNA-seq should be performed to obtain a genome-wide view on HDF induced changes in gonads (sperm and oocytes) of F0 and brains of F1 and at least F2.

>> (Response) **We agree that a broader spectrum analysis of transcriptional HFD effects on both brain and germline would be very interesting and we planned to perform these time- and money-consuming experiments in an independent follow-up study.**

8. The authors suggest that increased recruitment of HDAC2 to the BDNF promoter in gonads of HFD mother leads to lower BNDF expression in the brain of offspring. How is this phenomena than transmitted to F2 and F3? Is sperm in F1 and F2 affected by a similar mechanisms…and fi so how is this mediated?

>> (Response) **Additional experiments performed in p66Shc KO mice confirmed that maternal ovarian insulin resistance plays a critical role triggering the epigenetic alterations we found in both hippocampus and germline of next generations (Fig. 8; for more details, see response to concern #1 of Reviewer #1). We hypothesize that epigenetic changes occurring in F0 germ cells affect the descendants' epigenome since fertilization though we cannot exclude the possibility that the epigenetic marks identified on the *Bdnf* promoters primarily appear in the developing $F1_{HFD}$ embryos (see revised discussion at page 24, lines 530-539). The results of IVF experiments further confirm a gametic mechanism underlying the transgenerational transmission of epigenetic changes and related functional alterations.**

9. Is any tissue that expresses BNDF affected similarly or is the effect specific to gonads and hippocampus?

>> (Response) **RNA analysis performed in heart and muscle of F1 HFD mice showed a reduction of *Bdnf* expression similar to that observed in their hippocampus (Supplementary Fig. 3d), suggesting that the epigenetic regulation of neurotrophin expression is not limited to the brain.**

10. Related to point 10 assuming that BNDF signaling – in addition to other processes – may be affected at the body wide level: While weight, food intake and plasma glucose levels are similar in F1 generation the authors should confirm that motor behavior and other processes that may affect memory function are not altered in the F1-F3 generation.

>> (Response) **Locomotor activity and swimming speed have been investigated in F1-F2-F3 generations of HFD descendants. Our findings indicated no significant changes of motor behavior compared to controls (Supplementary Fig. 2i).**

Reviewers' comments:

Reviewer #1 (Remarks to the Author):

Overall, the authors did a fantastic and thorough job in addressing reviewer concerns. The addition of proper SD + NEE controls, cross-fostering and in vitro fertilization experiment clarify and strengthen their previous data significantly, although it is a bit unclear why SD + NEE doesn't have an effect on BDNF/cognitive performance, as other groups have shown this (at least in rats). This discrepancy should be acknowledged in the discussion.

I have issues with a couple of points that are still a bit unclear to me and a couple notes on wording their conclusions:

1) The peritoneal BDNF administration is confounded by the decreased food intake and resultant lack of weight gain. If this data is to be included (I am not sure they should be given this important confound, and the lack of connection to ovarian IR), the authors should also report glucose, insulin, HOMA-IR as they do for figures 1 and 2 as this may help to delineate if the primary signal might be weight gain, insulin resistance, glucose, etc. In that vein, the authors don't in any way link the BDNF levels and ovarian insulin resistance that they seem to conclude drives the intergenerational changes observed. They should at least show whether the BDNF injections alter insulin sensitivity in ovary as they have done with pIRS1/IRS1 ratios in figure 8. Consistency of what is being shown figure to figure both demonstrates authors are not picking and choosing only the data that fits their hypothesis and provides a better "through" logic and connectivity that supports a unified mechanism rather than what they have currently (which is that BDNF can rescue, as can reversal of ovarian IR, without any attempt to connect the two)
2) These data along with other corroborating data of BDNF expression levels being decreased in multiple tissues in offspring bring up the possibility that low plasma BDNF might be a mechanism for continued transmissions to successive generations. Have authors showed plasma BDNF levels in the F1-F3 generation? If they are lower, this is crucial data to include and perhaps provides a unifying mechanism (together with whether NEE changes BDNF levels). If not, it's a bit hard to follow how everything fits together.
3) The p66Shc data is very nice, though because it is a whole body knockout, it makes conclusions drawn re: the import of insulin resistance in the ovary versus other cell/tissue types impossible to discern. I believe the authors statement that the "critical role of ovarian insulin resistance as trigger of HFD-dependent transgenerational effects on hippocampal plasticity was highlighted by the results we obtained in p66Shc KO mouse model" is not substantiated and should be altered to acknowledge this. The authors could use recombinase lines to fiddle with insulin sensitivity in the ovary (there are floxed IRS1 an IR alleles with which they could do so) to be able to conclude this, but is outside the scope of this paper.
4) Similarly, their final conclusion: "However, alteration of both BDNF and insulin signaling in maternal gonads represent key molecular events triggering the epigenetic transmission of brain vulnerability to the next generations" is again overstated. BDNF signaling in other tissues (in the embryo itself, for instance) might drive this in the BCNF administration experiment, and insulin signaling in non-ovarian tssue could underlie p66Shc data as above. Again, no experiments to do, but more measured conclusions are required by the data presented.

Reviewer #2 (Remarks to the Author):

The revised submission of Fusco et al. reports on the impact of maternal high fat diet (before and during pregnancy/lactation) on the hippocampal learning/memory and LTP of the F1 offspring. The novel finding is that the initial maternal effect is transmitted to the F2 and F3 generations through the

paternal line. The authors posit that both the memory and LTP deficits are explained by reduced hippocampal BDNF expression (across the three generations), reduced transcription from BDNF promoters I, IV, and IXa, and by a reduction in active promoter-specific histone marks at these promoters. The main criticism was the correlative nature of the data and that other mechanisms, such as small RNAs in sperm/seminal fluid, may explain the transmission. To rectify this issue, the authors include new data showing that the BDNF gene is epigenetically marked in the male F1-F3 offspring (as a result of HFD mother) not only in the hippocampus, but also in male germline, as well as in muscle and heart at the above mentioned promoters. It seems that only exon IX expression is reduced in the male germline, as shown by a supplementary fig. There is no comment on whether the other promoters are silent in the germline or if they show no reduction. Even if negative, expression data are important and should be presented in a main figure. It is also unclear from the text if germline/gonadal tissue means sperm or more heterogeneous gonadal tissue. However, a more fundamental issue is that the authors interpret these data as proof for epigenetic transgenerational transmission. Because epigenetic marks are typically erased during early development, a more realistic interpretation is that the BDNF changes in sperm, like in somatic tissues, are secondary to a truly transgenerational mechanism (that can be linked to sperm/seminal fluid). Although a new IVF experiment strengthen the notion of a transgenerational transmission in the male line, it does not implicate BDNF histone changes as the underlying mechanism. It would be essential to demonstrate that the germline epigenetic changes are not erased and are fully maintained through early development and are disseminated across somatic tissues during differentiation. In addition, interference with the germline maintenance of the maternally induced changes should reverse the phenotype.

The section on how HFD in mothers leads, via a BDNF signaling mechanism, to the chromatin and expression changes at the BDNF gene itself in the ovary is interesting, but it still does not address the fundamental question of the transmission mechanism. Rather, this experiment explores a potential mechanism for the initiation of the maternal effect in the ovary. This can be viewed as an intergenerational effect, independent of the consecutive male line specific transgenerational effect. In some respect, the ovary-related study is more original than the male line transmission, but is premature at this stage because many confounds are not controlled for. Indeed, none of the manipulations is ovary specific and therefore could be due to systemic effects.

Overall, this manuscript tries to combine two separate studies to one cohesive story but the two lines of work do not strengthen each other. The male line specific transgenerational work is more mature but the conclusion of a BDNF chromatin/expression based mechanism is not supported by the data. The ovary-related work is more original but is less mature and needs additional data to support it.

Minor

It is confusing that crossfostered offspring is labeled no lactation (NL) for the offspring of F0 mothers while the crossfostered F2 offspring is called F2 CF.

The abstract is difficult to read and understand and does not entirely reflect the work.

Why are the authors use independent t-tests in the behavioral tests while two way ANOVAS in the chromatin/expression studies for data with two independent variables?

Manuscript NCOMMS-18-18649

POINT-BY-POINT RESPONSE TO THE REFEREES' COMMENTS

RESPONSE TO REVIEWER #1

Reviewer: Overall, the authors did a fantastic and thorough job in addressing reviewer concerns. The addition of proper SD + NEE controls, cross-fostering and in vitro fertilization experiment clarify and strengthen their previous data significantly, although it is a bit unclear why SD + NEE doesn't have an effect on BDNF/cognitive performance, as other groups have shown this (at least in rats). This discrepancy should be acknowledged in the discussion.

>> (Authors' Response) **We are very grateful to the referee for his/her comments and appreciation. Following their suggestion, we included in the discussion some comments on the results obtained from the offspring of SD NEE mice (pages 23-24, lines 503-506). However, we want to stress that in our experimental model exposure to NEE did improve cognitive functions and increased Bdnf expression in the hippocampus of animals environmentally enriched (data not shown), thereby confirming the efficacy of our NEE experimental protocol.**

Reviewer: I have issues with a couple of points that are still a bit unclear to me and a couple notes on wording their conclusions. The peritoneal BDNF administration is confounded by the decreased food intake and resultant lack of weight gain. If this data is to be included (I am not sure they should be given this important confound, and the lack of connection to ovarian IR), the authors should also report glucose, insulin, HOMA-IR as they do for figures 1 and 2 as this may help to delineate if the primary signal might be weight gain, insulin resistance, glucose, etc. In that vein, the authors don't in any way link the BDNF levels and ovarian insulin resistance that they seem to conclude drives the intergenerational changes observed. They should at least show whether the BDNF injections alter insulin sensitivity in ovary as they have done with pIRS1/IRS1 ratios in figure 8. Consistency of what is being shown figure to figure both demonstrates authors are not picking and choosing only the data that fits their hypothesis and provides a better "through" logic and connectivity that supports a unified mechanism rather than what they have currently (which is that BDNF can rescue, as can reversal of ovarian IR, without any attempt to connect the two)

>> (Authors' Response) **The manuscript revision addressed this reviewer's request for "consistency of what is being shown figure to figure". Specifically, to further investigate the link between BDNF levels and ovarian insulin resistance we performed metabolic analyses on plasma collected from HFD mothers injected with BDNF. Our findings indicate that BDNF administration did not significantly affect the HFD-dependent peripheral insulin resistance (see revised Fig. 7c and Supplementary Figure 6a). More importantly, it did not significantly revert the inhibitory hyperphosphorylation of IRS in the ovaries (Fig. 7d). Our data suggest that both BDNF deficit and altered insulin signaling contribute to the intergenerational transmission of HFD-dependent cognitive impairment, as mentioned in the Results (pages 17-18, lines 380-382 and page 21, lines 439-441).**

Reviewer: These data along with other corroborating data of BDNF expression levels being decreased in multiple tissues in offspring bring up the possibility that low plasma BDNF might be a mechanism for continued transmissions to successive generations. Have authors showed plasma BDNF levels in the F1-F3 generation? If they are lower, this is crucial data to include and perhaps provides a unifying mechanism (together with whether NEE changes BDNF levels). If not, it's a bit hard to follow how everything fits together.

>> (Authors' Response) **According to the multiple organ downregulation of *Bdnf* expression in HFD descendants, we found lower levels of this neurotrophic factor in the plasma of F1-F3 HFD progeny (Supplementary Fig. 3e). Moreover, lower levels of circulating BDNF may contribute to the epigenetic inhibition of *Bdnf* gene in both gametic and somatic tissues of HFD male descendants, as reported in the discussion (page 23, lines 482-484).**

Reviewer (3): The p66Shc data is very nice, though because it is a whole body knockout, it makes conclusions drawn re: the import of insulin resistance in the ovary versus other cell/tissue types impossible to discern. I believe the authors statement that the "critical role of ovarian insulin resistance as trigger of HFD-dependent transgenerational effects on hippocampal plasticity was highlighted by the results we obtained in p66Shc KO mouse model" is not substantiated and should be altered to acknowledge this. The authors could use recombinase lines to fiddle with insulin sensitivity in the ovary (there are floxed IRS1 an IR alleles with which they could do so) to be able to conclude this, but is outside the scope of this paper.

>> (Authors' Response) **We agree with the reviewer that data obtained in the *p66Shc* KO mice do not allow to discern the role of insulin resistance in the ovary versus other cell/tissue types. Accordingly, our statement has been reformulated (see page 25, lines 533-535).**

Reviewer (4): Similarly, their final conclusion: "However, alteration of both BDNF and insulin signaling in maternal gonads represent key molecular events triggering the epigenetic transmission of brain vulnerability to the next generations" is again overstated. BDNF signaling in other tissues (in the embryo itself, for instance) might drive this in the BCNF administration experiment, and insulin signaling in non-ovarian tssue could underlie p66Shc data as above. Again, no experiments to do, but more measured conclusions are required by the data presented.

>> (Authors' Response) **Our conclusions have been reformulated on the basis of the reviewer's suggestion (pages 17-18, lines 380-382 and page 21, lines 439-441; page 25 lines 538-539).**

RESPONSE TO REVIEWER #2

Reviewer: The revised submission of Fusco et al. reports on the impact of maternal high fat diet (before and during pregnancy/lactation) on the hippocampal learning/memory and LTP of the F1 offspring. The novel finding is that the initial maternal effect is transmitted to the F2 and F3 generations through the paternal line. The authors posit that both the memory and LTP deficits are explained by reduced hippocampal BDNF expression (across the three generations), reduced transcription from BDNF promoters I, IV, and IXa, and by a reduction in active promoter-specific histone marks at these promoters. The main criticism was the correlative nature of the data and that other mechanisms, such as small RNAs in sperm/seminal fluid, may explain the transmission.

>> (Authors' Response) **The authors agree that other epigenetic and/or non-epigenetic mechanisms (including small RNAs in sperm/seminal fluid) may be causally linked to the cognitive impairment observed in HFD descendants and involved in the transgenerational transmission of the phenotype across the HFD progeny. This matter has been discussed in the revised manuscript (page 23, lines 482-487; page 25 lines 541-547).**

Reviewer: To rectify this issue, the authors include new data showing that the BDNF gene is epigenetically marked in the male F1-F3 offspring (as a result of HFD mother) not only in the hippocampus, but also in male germline, as well as in muscle and heart at the above mentioned promoters. It seems that only exon IX expression is reduced in the male germline, as shown by a supplementary fig. There is no comment on whether the other promoters are silent in the germline or if they show no reduction. Even if negative, expression data are important and should be presented in a main figure.

>> (Authors' Response) **In the male germline, we had limited our analyses to expression of exon IX because it is the only coding exon, which reflects the BDNF expression in the tissues at protein level. Moreover, we showed that *Bdnf* promoters I, IV and IX were silenced in the germline of HFD descendants (Fig. 4c). However, following the reviewer's suggestion, we performed RNA expression analysis of all BDNF exons in the germline of HFD progeny. Exons II-V and VII-VIII were not detectable; the revised Figure 4d shows the expression of exons I, VI and XIa.**

Reviewer: It is also unclear from the text if germline/gonadal tissue means sperm or more heterogeneous gonadal tissue.

>> (Authors' Response) **Molecular analyses of male germline were performed on sperm as indicated in Methods at page 30, lines 662-666 and lines 672-677.**

Reviewer: However, a more fundamental issue is that the authors interpret these data as proof for epigenetic transgenerational transmission. Because epigenetic marks are typically erased during early development, a more realistic interpretation is that the BDNF changes in sperm, like in somatic tissues, are secondary to a truly transgenerational mechanism (that can be linked to sperm/seminal fluid). Although a new IVF experiment strengthen the notion of a transgenerational transmission in the male line, it does not implicate BDNF histone changes as the underlying mechanism. It would be essential to demonstrate that the germline epigenetic changes are not erased and are fully maintained through early development and are disseminated across somatic tissues during differentiation. In addition, interference with the germline maintenance of the maternally induced changes should reverse the phenotype.

>> (Authors' Response) **Our finding that F1, F2 and F3 generations exhibited the same epigenetic changes on the *Bdnf* promoters in both hippocampus and germline (Fig. 4c,e) is compatible with the hypothesis that these epigenetic changes are maintained through early development. Nevertheless we agree with the reviewer that several mechanisms other than the observed Bdnf epigenetic changes may underlie or contribute to both the cognitive phenotype and its transmission. Therefore discussion was revised accordingly (see page 23, lines 482-487; page 25, lines 541-547).**

Reviewer: The section on how HFD in mothers leads, via a BDNF signaling mechanism, to the chromatin and expression changes at the BDNF gene itself in the ovary is interesting, but it still does not address the fundamental question of the transmission mechanism. Rather, this experiment explores a potential mechanism for the initiation of the maternal effect in the ovary. This can be viewed as an intergenerational effect, independent of the consecutive male line specific transgenerational effect. In some respect, the ovary-related study is more original than the male line transmission, but is premature at this stage because many confounds are not controlled for. Indeed, none of the manipulations is ovary specific and therefore could be due to systemic effects.
Overall, this manuscript tries to combine two separate studies to one cohesive story but the two lines of work do not strengthen each other. The male line specific transgenerational work is more mature but the conclusion of a BDNF chromatin/expression based mechanism is not supported by the data. The ovary-related work is more original but is less mature and needs additional data to support it.

>> (Authors' Response) **We revised the manuscript based on the reviewer's concerns, and defined as "intergenerational" the mother to offspring transmission of the HFD-dependent damage. With regard to the male line specific transgenerational effect, the results of new experiments demonstrated a decrease of BDNF plasma levels in all HFD descendants, which adds a novel layer to the epigenetic regulation of *Bdnf* gene in the male germline (Supplementary Fig. 3e). In addition, we discussed other epigenetic and non-epigenetic mechanisms potentially involved in the transgenerational effects of HFD on cognitive function (page 23, lines 482-484; page 23, lines 500-503; page 25, lines 541-547). Finally, we modified our conclusions about p66Shc experiments stressing that this experimental model is not ovary-specific and, therefore, systemic effects might underly the observed responses (page 19, lines 405-406; page 21 lines 439-441; page 25, lines 533-535 and 538-539).**

Minor

Reviewer: It is confusing that crossfostered offspring is labeled no lactation (NL) for the offspring of F0 mothers while the crossfostered F2 offspring is called F2 CF.

>> (Authors' Response) **Probably, there was a misunderstanding. Data shown in Supplementary Figure 2b-c were not obtained from crossfostered mice. $F2_{HFD\ NL}$ and $F3_{HFD\ NL}$ mice descended from a progenitor F0 female fed with HFD until the birth of pups. After the delivery, we changed the maternal dietary regimen from high fat diet to standard one but leaving the offspring with its own mother. We tried to better clarify this experimental protocol in the revised Results (page 8, lines 171-178). Conversely, data shown in Supplementary Figure 4a-d (i.e., $F1_{HFD}$ CF) were collected from mice born from HFD mothers and fostered by different SD-fed female mice.**

Reviewer: The abstract is difficult to read and understand and does not entirely reflect the work.

>> (Authors' Response) **The abstract was revised to address the reviewer's concern.**

Reviewer: Why are the authors use independent t-tests in the behavioral tests while two way ANOVAS in the chromatin/expression studies for data with two independent variables?

>> (Authors' Response) **We used unpaired Student's *t*-test for behavioral analyses shown in Figures 2 and 3 where SD mice were compared with either F1 HFD or F2 HFD or F3 HFD mice in different experimental trials. For RT PCR and ChIP studies shown in Figure 4, samples from SD mice were compared with those of all HFD descendants in the same experiments, and statistics was performed by one-way ANOVA and Bonferroni post hoc. Finally, for experiments with two independent variables (e.g., Figure 5 and 7, Supplementary Figure 5) we used two way ANOVA.**

RESPONSE TO REVIEWER #3

Reviewer: the reviewer states: The link to BNDF signaling via the epigenetic regulation of the BNDF gene is correlative. The rescue experiments using novelty environmental enrichment in the F2 generation are not conclusive. Enrichment improves memory consolidation in general,…..The authors misunderstood this question, as they argue for a transgenerational reversal effect in general, but the question was about the role of BDNF in the (transgenerational) phenotype as well as in its reversal. It is plausible that a BDNF unrelated mechanism disrupts the "transgenerational" mark to be expressed or transmitted further down in the

lineage. So the author's response is not adequate, but they eventually address the question in the next section, see below.

>> (Authors' Response) **We agree with the reviewer that our findings do not unequivocally demonstrate that the intergenerational effects of NEE are due to the epigenetic regulation of BDNF, and we revised the discussion, accordingly (see page 23, lines 500-503).**

Reviewer: The authors performed the suggested experiments and show that F0 maternal BDNF administration reverses the various maternal HFD-induced phenotypes in the F1. However, this experiment was not continued to F2 and F3, so the authors can claim an intergenerational, but not transgenerational effects. This differentiation is significant because in their model the initial maternal effect on the F1 is transmitted, from the F1 to F2-F3 via the male germline. However, given the time required for an F1-F3 experiment, my view is that the authors made a reasonable effort to address the question and found a reversal effect of BDNF in F1 that is potentially (but not necessarily as they seem to claim) carried over to F2-F3.

>> (Authors' Response) **We are grateful to the reviewer for his/her words of appreciation toward our revision work. In the revised manuscript all the effects observed only in F1 have been reported as "intergenerational".**

Reviewer: Sperm isolation is now described, but the reviewer also raised the question of how an F0 ovarian, presumably epigenetic, change by HDF is transmitted to the F1 sperm. This issue is not addressed in the revised manuscript. I also pointed out this in my review because F0 germline epigenetic marks are typically erased in the F1 early embryo, preventing their direct transmission to the F1 sperm.

>> (Authors' Response) **Whether the epigenetic marks can be passed on from mammalian gametes to the next generation is a long-standing question that still remains unanswered (see page 25, lines 539-541). However, in the revised manuscript we discussed different mechanisms potentially involved in the intergenerational transmission of HFD effects on cognitive function, including the possibility that brain damage primarily occurs in the developing F1 embryos and lead to multi-organ inhibition of Bdnf expression (page 25, lines 541-547).**

Reviewer: The authors addressed the maternal care issue by crossfostering. The reviewer also mentioned IVF that is not for testing the possible effect of maternal care. Rather, as the authors point out correctly, it is designed to differentiate between germline and somatic transmission. Their experiment indicates a gametic mechanism of transmission. Overall, the authors responded to this point.

>> (Authors' Response) **We thank the reviewer for his/her comments and appreciation.**

Reviewer: The authors should have used litter as n, but employed different strategies to minimize litter effect. Crossfostering does not address the issue as this controls only for postnatal effect, while here the maternal effect could be gestational.

>> (Authors' Response) **The authors are grateful to the reviewer for his/her appreciation toward the experimental strategies used to minimize the litter effect.**

Reviewer: The authors should comment on the possibility of RNA dependent mechanism. My view is that an RNA mediated mechanism is not just an epiphenomenon, but is not germline either. The reason is that sperm small RNAs can derive from non-germline source via exosomes. So, I prefer to use gametic that includes nuclear epigenetic (genuine germline) as well as acquired small RNAs.

>> (Authors' Response) **The revised discussion includes comments about a potential RNA-dependent mechanism underlying the transgenerational transmission of the cognitive impairment (page 25, lines 543-544 and 549-551). The concept that that "gametic" mechanisms are responsible for the observed effects has been introduced in the title and throughout the text (see page 2, lines 39-40; page 11, line 250; page 12 line 256).**

Reviewer: I agree with the reviewer, but the hypothesis here was testing BDNF that can be done without RNA-Seq.

>> (Authors' Response) **We thank the reviewer for considering our approach appropriate.**

Reviewer: As I mentioned in my criticism, this is the most puzzling part of the paper (how a maternal gonadal epigenetic change survives reprogramming in the embryo). I think the authors do not effectively address this issue.

>> (Authors' Response) **It is still debated if the mouse embryo can retain the histone modifications that were acquired during oocyte maturation or they are completely erased during embryo development (Perez and Lehner, Nat Cell Biol 2019). However, it is clear that epigenetic transmission of behavioral traits may occur through different routes including a combination of behavioral reinforcement (*e.g.* maternal behavior affecting offspring), direct somatic programming (*e.g. in utero* exposure to maternal environment), and both transient and stable programming of the germline (Youngson and Whitelaw, Annu Rev Genomics Hum Genet, 2008; Franklin et al., Biol Psychiatry 2010; Kim et al., Crim Behav Mental Health 2009; Vigé et al., Pediatr Res, 2008). In addition, each generation may add secondary programming influences such as the presence of somatic phenotypes (e.g. changes of neuroendocrine signals). Actually, our data do not provide direct evidence that maternal gonadal epigenetic changes survive embryo reprogramming. Nonetheless, the germline-based intergenerational alteration of cognitive function we found in F1-F3 were paralleled by the same epigenetic marks in all generations thus, at least, leaving open the possibility that these changes are not erased. However, as already mentioned in our previous answer, in the revised discussion we acknowledged that different mechanisms may be involved in the intergenerational transmission of HFD effects on cognitive function (page 25, lines 538-547).**

REVIEWERS' COMMENTS:

Reviewer #1 (Remarks to the Author):

First, I want to apologize profusely to the editors and authors for the tardiness of this review, especially given that in the end, I have no constructive comments or criticisms.

The authors again did a fantastic and thorough job in addressing my and other reviewer concerns, so kudos to them. The addition of the serum BDNF levels and numerous revisions limiting their conclusions serve to clarify greatly the strength and import of their work by specifying which of their findings are correlative and which are driving mechanisms.

As I reread the manuscript, I hope the authors found that their manuscript has been enhanced measurably by the review process. I have no further suggestions or comments at this time.

Reviewer #2 (Remarks to the Author):

1) The revised text and additional data improved the result section. However, it seems that the authors avoid presenting a coherent picture or model that connects the numerous pieces of data presented in the result section. The impact of the paper would significantly increase by the presentation of an easy to understand flow chart or summary fig. This chart should outline 1) how HFD in mothers may lead to the molecular and behavior changes in F1 males and 2) how the F1 molecular and behavioral changes propagate across F2 and F3. The understanding of this reviewer is that the authors link HFD in the mother via IRS signaling, CREB, and HAT/HDAC to BDNF regulation; but they also mention a parallel signal via FOXO. They discuss that both pathways are required for the behavioral phenotype, but the phenotype can be rescued by targeting each pathway individually. Further confusion is that BDNF rescues the cognitive defect but it does not alter insulin resistance. But then, the authors show that p66Shh KO rescues the cognitive phenotype. I am concerned that without a clear and logical summary figure and associated interpretation, this work will not be understood by most readers and importantly, will not be remembered and cited.
2)The discussion in its present form reiterates the data with the interpretation of individual findings, and it is way too long. Instead, discussion should be the interpretation of ALL data together with a summary figure, as suggested above.

Manuscript NCOMMS-18-18649

POINT-BY-POINT RESPONSE TO THE REFEREES' COMMENTS

RESPONSE TO REVIEWER #1

Reviewer: First, I want to apologize profusely to the editors and authors for the tardiness of this review, especially given that in the end, I have no constructive comments or criticisms.

The authors again did a fantastic and thorough job in addressing my and other reviewer concerns, so kudos to them. The addition of the serum BDNF levels and numerous revisions limiting their conclusions serve to clarify greatly the strength and import of their work by specifying which of their findings are correlative and which are driving mechanisms.

As I reread the manuscript, I hope the authors found that their manuscript has been enhanced measurably by the review process. I have no further suggestions or comments at this time.

>> (Authors' Response) **We are very grateful to the referee for his/her kind word of appreciation and the very useful comments that helped us to improve our work.**

RESPONSE TO REVIEWER #2

Reviewer:

1) The revised text and additional data improved the result section. However, it seems that the authors avoid presenting a coherent picture or model that connects the numerous pieces of data presented in the result section. The impact of the paper would significantly increase by the presentation of an easy to understand flow chart or summary fig. This chart should outline 1) how HFD in mothers may lead to the molecular and behavior changes in F1 males and 2) how the F1 molecular and behavioral changes propagate across F2 and F3. The understanding of this reviewer is that the authors link HFD in the mother via IRS signaling, CREB, and HAT/HDAC to BDNF regulation; but they also mention a parallel signal via FOXO. They discuss that both pathways are required for the behavioral phenotype, but the phenotype can be rescued by targeting each pathway individually. Further confusion is that BDNF rescues the cognitive defect but it does not alter insulin resistance. But then, the authors show that p66Shh KO rescues the cognitive phenotype. I am concerned that without a clear and logical summary figure and associated interpretation, this work will not be understood by most readers and importantly, will not be remembered and cited.

>> (Authors' Response) **We added a novel picture (Figure 9) describing the flow chart of our findings.**

2) The discussion in its present form reiterates the data with the interpretation of individual findings, and it is way too long. Instead, discussion should be the interpretation of ALL data together with a summary figure, as suggested above.

>> (Authors' Response) **We modified the discussion according to the Reviewer's suggestions.**
